# NMAsurv: An R Shiny application for network meta-analysis based on survival data

**DOI:** 10.1017/rsm.2025.10020

**Published:** 2025-07-10

**Authors:** Taihang Shao, Mingye Zhao, Fenghao Shi, Mingjun Rui, Wenxi Tang

**Affiliations:** 1 Center for Pharmacoeconomics and Outcome Research, China Pharmaceutical University, Nanjing, China; 2 JC School of Public Health and Primary Care, Faculty of Medicine, The Chinese University of Hong Kong, Shatin, China; 3 International Research Center for Medicinal Administration, Peking University, Beijing, China; 4 School of Pharmacy, Faculty of Medicine, The Chinese University of Hong Kong, Shatin, China

**Keywords:** network meta-analysis, non-proportional hazards, parameter estimation, R Shiny, survival data

## Abstract

Network meta-analysis (NMA) is becoming increasingly important, especially in the field of medicine, as it allows for comparisons across multiple trials with different interventions. For time-to-event data, that is, survival data, traditional NMA based on the proportional hazards (PH) assumption simply synthesizes reported hazard ratios (HRs). Novel methods for NMA based on the non-PH assumption have been proposed and implemented using R software. However, these methods often involve complex methodologies and require advanced programming skills, creating a barrier for many researchers. Therefore, we developed an R Shiny tool, NMAsurv (https://psurvivala.shinyapps.io/NMAsurv/). NMAsurv allows users with little or zero background in R to conduct survival-data-based NMA effortlessly. The tool supports various functions such as drawing network plots, testing the PH assumption, and building NMA models. Users can input either reconstructed pseudo-individual participant data or aggregated data. NMAsurv offers a user-friendly interface for extracting parameter estimations from various NMA models, including fractional polynomial, piecewise exponential models, parametric survival models, Cox PH model, and generalized gamma model. Additionally, it enables users to effortlessly create survival and HR plots. All operations can be performed by an intuitive “point-and-click” interface. In this study, we introduce all the functionalities and features of NMAsurv and demonstrate its application using a real-world NMA example.

## Highlights

### What is already known?

- Network meta-analysis (NMA) based on survival data is becoming increasingly important, while the traditional Cox proportional hazards (PH) model, which relies on the PH assumption, cannot handle some novel evidence.
- More flexible, non-PH assumption-based models have been proposed, but they are limitedly used probably due to their complex methodology and programming.

### What is new?

- NMAsurv is presented as the first tool for handling time-to-event data using non-PH assumption-based NMA models.
- NMAsurv offers several useful functionalities, including network plot generation, PH assumption testing, and batch export of results, to facilitate NMA studies.
- NMAsurv provides direct parameter estimations and generates survival plots and hazard ratio plots, enabling users to obtain comprehensive evidence synthesis results efficiently.

### Potential impact for RSM readers

- NMAsurv features an intuitive “point-and-click” interface, allowing users with little or no background in R to follow the provided user manual to conduct an NMA based on survival data and generate comprehensive results step-by-step without installing any software.

## 1 Introduction

Evidence synthesis is an important component of health technology assessment, through which decision-makers can combine data from multiple trials to get a pooled estimate of clinical efficacy.^1^ Pairwise meta-analysis is suitable when comparing two interventions. However, if more than two interventions are of interest, and the available trials do not individually compare all interventions, then network meta-analysis (NMA) should be considered.^2^ NMA can be an extension of pairwise meta-analysis, which can estimate the comparative effectiveness of multiple interventions simultaneously through building a connected network.^3^

The efficacy of novel anti-cancer interventions is frequently measured using time-to-event (TTE) outcomes, such as progression-free survival (PFS) and overall survival (OS), which are often presented through Kaplan–Meier (KM) curves. Typically, the relative treatment effect between two interventions is expressed as a hazard ratio (HR) or restricted mean survival time (RMST).^4^^–^ ^6^ NMA based on TTE outcomes can be constructed directly through trial-specific reported HRs, which are usually derived from the Cox proportional hazards (PH) model.^7^ However, the PH assumption may sometimes be violated because different trials may exhibit variations in follow-up times or compare interventions with different mechanisms of action.^6^^,^ ^8^^,^ ^9^ In this case, continuing to use the Cox PH model may lead to biased estimates.

To address this issue, alternative NMA models that relax the PH assumption have been developed.^1^^,^ ^7^^,^ ^10^ Nonetheless, these methods have seen limited adoption.^11^ One possible barrier is that researchers are not familiar with the detailed methodology of these models, which can be essential to their correct application.^7^ Another challenge is the difficulty in implementing these methods, as they often require advanced programming skills. While user-friendly web-based tools or software for traditional NMA based on binary or continuous outcomes exist,^12^^–^ ^15^ tools specially designed for NMA based on TTE outcomes are not yet available.

To break the barriers of using flexible NMA methods to process the TTE outcomes, we created an R Shiny framework-based interactive application: NMAsurv. In this technical note, we first give a brief review of currently existing NMA methods and introduce the methodology of the methods included in this app in Section 2. In Section 3, we describe the structure and functionality of NMAsurv. In Section 4, an illustrative example showing how to use NMAsurv is provided. Finally, in Section 5, we discuss the advantages and limitations of NMAsurv, as well as potential future extensions. A user manual for NMAsurv can be found in Supplementary Material 1.

## 2 Methods

### 2.1 NMA for TTE outcomes

Typically, when conducting NMA based on survival data, researchers often lack access to IPD (Individual Patient Data) for all included trials. In cases where IPD is available for one or more trials, alternative population-adjusted indirect comparison methods, such as simulated treatment comparison (STC) and matching-adjusted indirect comparison (MAIC), can be employed for two-study scenarios. Multilevel network meta-regression can be used for large treatment networks.^12^^–^ ^15^ These methods are well developed, and some user-friendly tools are also available.^16^^–^ ^18^ However, it is more common for researchers to lack access to IPD. In such cases, techniques can be used to digitize published OS and PFS KM curves and reconstruct pseudo-IPD.^19^^–^ ^21^ In NMAsurv, we only considered reconstructed pseudo-IPD as our data input.

### 2.2 A brief review of the NMA models

We conducted a systematic review in PubMed to identify published articles containing NMA models based on survival data. The detailed search strategy was provided in Supplementary Material 2. Additionally, we reviewed previously published reviews^1^^,^ ^7^^,^ ^10^^,^ ^22^^,^ ^29^^,^ ^30^ and guidelines^31^^,^ ^32^ on NMA for TTE results. After screening, we finally identified the following eight methods: the Cox PH model, fractional polynomial (FP) model, piecewise exponential (PWE) model, parametric survival model (PSM), Royston–Parmar (RP) model, RMST, generalized gamma model, and cure models. A brief introduction and related citations for these models can be found in Table 1. Furthermore, researchers can add treatment-by-covariates interactions in NMA to evaluate multivariate treatment effects.^33^ We do not include treatment-by-covariates interactions in our discussion as it requires access to the true IPD with covariates.

**Table 1:** A brief introduction of included models

Models	Brief introduction	Citation
Cox proportional hazards model	Rely on the PH assumption; HR is considered constant all the time.	^7^
Fractional polynomial model	Rely on the non-PH assumption; can be fitted in one-step or two-step process; can be constructed based on both AD or IPD.	^7^^,^ ^22^^,^ ^23^
Piecewise exponential model	Rely on the non-PH assumption, but in each time interval, the HR is considered constant; can be fitted in one-step or two-step process; can be constructed based on both AD or IPD.	^7^^,^ ^10^^,^ ^22^
Parametric survival model	Rely on the non-PH assumption; distributions can be considered including Weibull, Gompertz, log-logistic, and log-normal; can be fitted in one-step or two-step process; can be constructed based on AD or IPD.	^24^^,^ ^25^^,^ ^26^
Royston–Parmar model	Rely on the non-PH assumption; typically consider one to three internal knots; can be fitted in one-step process; can be constructed based on IPD.	^7^^,^ ^27^
Restricted mean survival time	Rely on the non-PH assumption; should select a time point that is less than or equal to the minimum value of the largest observed survival time across all trials; the results cannot be extrapolated; can be fitted in two-step process; can be constructed based on IPD.	^7^^,^ ^28^
Generalized gamma model	Rely on the non-PH assumption; can be fitted in two-step process; can be constructed based on IPD.	^7^
Cure model	Rely on the non-PH assumption; include mixture and non-mixture models; can be fitted in one-step process; can be constructed based on IPD.	^10^

Abbreviations: AD, aggregated data; HR, hazard ratio; IPD, individual participant data; PH, proportional hazards.

### 2.3 Methodology of included NMA models

Based on the review in Section 2.2, we finally included five methods in this app: the Cox PH model, FP model, PWE model, PSM, and generalized gamma model. Cure models were excluded from this study since the current practice is not based on R. The RP model and the RMST model were excluded because the existing algorithms do not support “point-and-click” operations. Further discussion on these excluded methods can be found in Section 5. A summary of the five implemented methods along with their respective data and model assumptions is provided in Table 2.

**Table 2:** A summary of the five implemented methods and their data/model assumptions

Data availability/					Generalized gamma
approaches	FP model	PWE model	PSM	Cox PH model	model
One-step IPD	#	#			
Two-step IPD			$		
One-step AD					
Two-step AD					
Frequentist			*		
Bayesian					
FE model					
RE model				§	

*Note*: Green = implemented in the app; red = supported in the literature but not implemented; gray = not applicable. The symbol “*” indicates that PSM under the frequentist framework can only be realized through the two-step IPD approach. The symbol “#” refers to Freeman et al. (2022).^7^ The symbol “$” refers to Cope et al. (2020).^24^ The symbol “§” refers to Bowden et al. (2011).^34^ Abbreviations: AD, aggregated data; FE, fixed-effect; FP, fractional polynomial; IPD, individual participant data; PH, proportional hazard; PSM, parametric survival model; PWE, piecewise exponential; RE: random-effects. This table contains information updated up to July 31, 2024.

#### 2.3.1 Fractional polynomial model

In current practice, two approaches are commonly used to implement FP NMA: (1) a one-step, IPD-based method^7^ and (2) a two-step, AD-based method.^22^ In this study, we employed the two-step, AD-based method. In the first step, an ANOVA-like parameterization is used to express and fit the model as a generalized linear model (GLM) with time-varying covariates within a frequentist framework. The models are then compared based on the Akaike information criterion (AIC), and the model with the lowest AIC is selected for further analysis in a Bayesian or frequentist setting in the second step. This two-step approach can speed up the model selection process.^7^^,^ ^22^ Typically, an FP model can be written as follows^23^:(1)

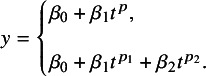



Power *p* can be −2, −1, −0.5, 0, 0.5, 1, 2, and 3 with 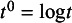 , if 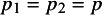 and 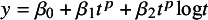 . For reference treatment *a* and intervention *b*, taking the FP1 model with power = −2 as an example, we get *d*_0*ab*_ *, d*_1*ab*_ *, β*_0*a*_ *, β*_1*a*_ *, β*_0*b*_, and *β_1b_*. To be specific,(2)

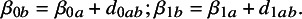



Thus, we can get the following formulas:(3)

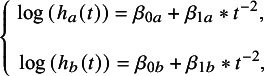


(4)

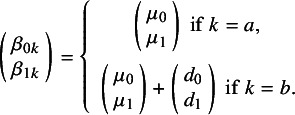



Here, *d* is the difference in *β*_0_ and *β*_1_ of the log hazard for treatment *b* relative to *a* and *μ* is the parameters *β* of the reference treatment. The methodology of calculation in both the Bayesian setting and the frequentist setting has been described in detail elsewhere.^22^^,^ ^23^ For the random-effects model, we consider only a heterogeneity parameter for *d*_0_ as the methodology for including all heterogeneity parameters still needs further development.^23^

#### 2.3.2 Piecewise exponential model

The PWE model follows a framework similar to that of the FP model, employing a two-step, AD-based method.^22^ However, currently, only fixed-effect models are available for PWE. For reference treatment *a* and intervention *b*, taking a PWE model with time point = 2 as an example, we get *d*_0*ab*_ *, d*_1*ab*_ *, β*_0*a*_ *, β*_1*a*_ *, β*_0*b*_, and *β*_1*b*_. We can get formula (5) based on formulas (2) and (4):(5)

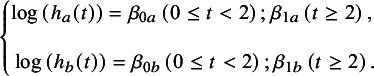



#### 2.3.3 Parametric survival model

In current practice, there are also two ways to realize PSM.^24^^,^ ^25^ Here, we applied a one-step, AD-based method. The models including Weibull, Gompertz, log-logistic, and log-normal are considered. Since frequentist analysis based on GLM is not available, we only include the Bayesian analysis. For reference treatment *a* and intervention *b*, taking the PSM Weibull model as example, based on formulas (2) and (4), we can get formula (6):(6)

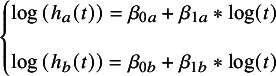



The log hazard function formulas for all four distributions can be found in Supplementary Material 2.

#### 2.3.4 Cox PH model

The NMA model based on the PH assumption can be realized in several ways. In this app, we select the method developed by Freeman et al.,^7^ which follows a two-step, IPD-based approach. Further discussion on the NMA model based on the PH assumption can be found in Section 5. In the first step, a Cox PH model is fitted individually to each trial to obtain an estimate of the log HR for the treatment effect and its corresponding standard error (SE):(7)

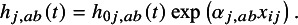



In formula (7), *h_j,ab_(t)* is the hazard function for treatment *b* compared to the baseline treatment *a* in trial *j*, *h_0j,ab_(t)* is the baseline hazard function for trial *j*, *x_ij_* is the treatment indicator variable for patient *i* from trial *j*, and *α_j,ab_* refers to the HR for a patient receiving treatment *b* compared to the baseline treatment *a* in trial *j*. In the second step, the treatment effect estimates are synthesized through a fixed-effect NMA model.^7^

#### 2.3.5 Generalized gamma model

The generalized gamma model is fitted through a two-step, IPD-based method.^7^ Each trial is independently evaluated by employing the generalized gamma model in the first step to get treatment effect estimates (log HR). In the second step, these treatment effect estimates of the baseline treatment compared to treatment *i* in trial *j* are synthesized, and their variability is estimated within a standard fixed-effect NMA model. The probability density function for the generalized gamma model can be found in Supplementary Material 2.

#### 2.3.6 Calculation of HR

For FP, PWE, and PSM, the HR is time-varying, meaning that HR can be written as a function of time. The calculation of HR can be written as formula (8) according to formulas (2)–(6):(8)

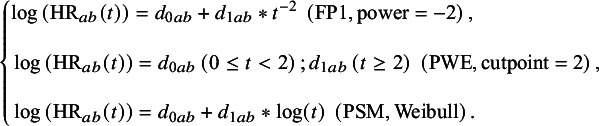



For the Cox PH model, the HR is *time-invariant* and does not require further calculation. For the generalized gamma model, the direct output of the model is the treatment effect. Typically, researchers have to use some functions in R to get the hazard function and the survival function.^35^ In order to get the time-varying HR, the relationship between HRs, the cumulative hazard function, and the survival function should be considered.

## 3 R Shiny app

### 3.1 Brief introduction

This web-based tool was constructed through the R Shiny package. Currently, most of the NMA models for survival data can be realized through R. This means that interactive data analysis and visualization with R are allowed. Users who do not have too much experience in R can also use this tool since they do not need to know the exact packages and functions to build models. Even installing R is not necessary since NMAsurv was deployed on shinyapps.io, which is a webserver. NMAsurv can be easily accessed through the URL https://psurvivala.shinyapps.io/NMAsurv/. The names of the R packages used to construct this tool can be found in Supplementary Material 2.

### 3.2 Functionalities and features

**Figure 1:**
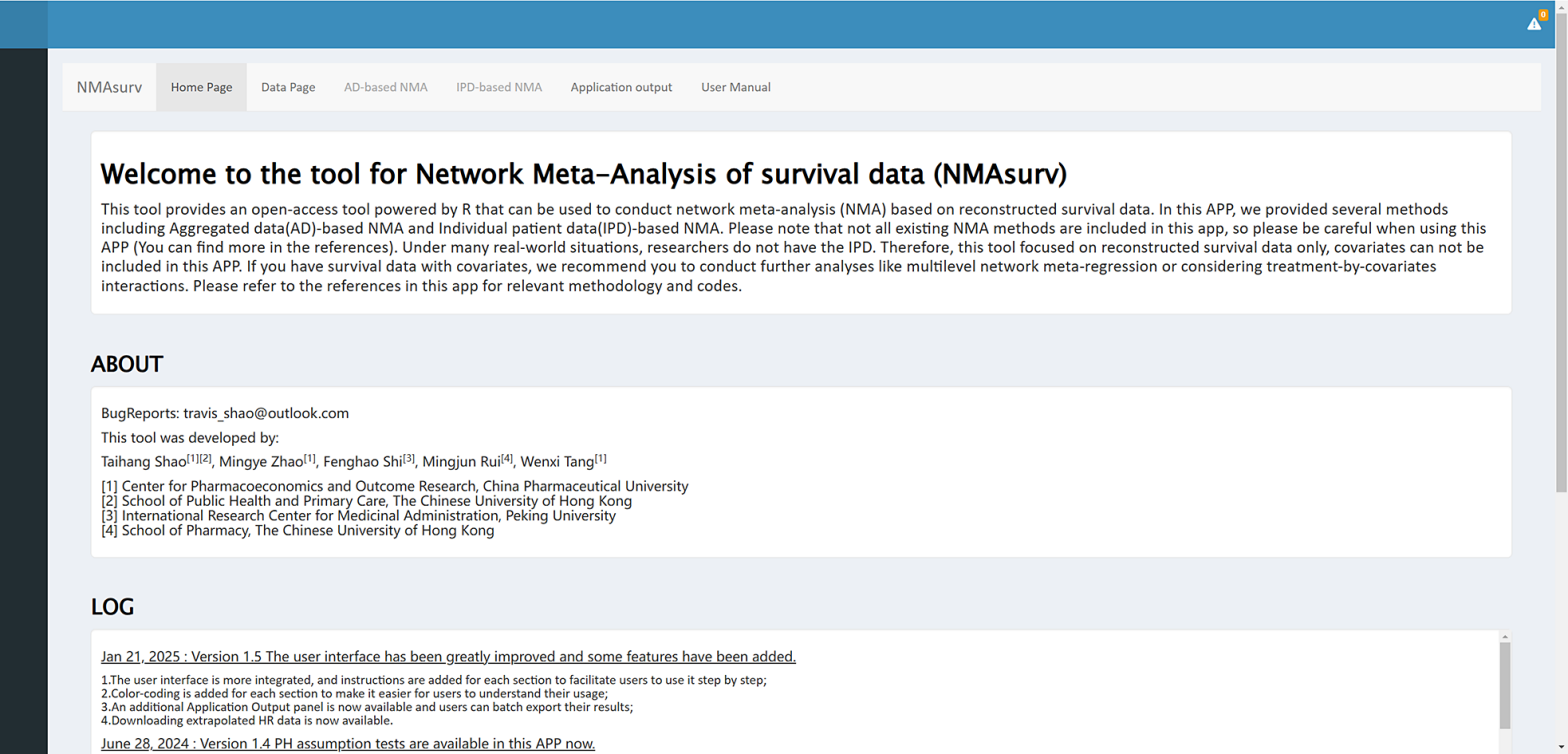
The interface of the homepage of NMAsurv.

Our goal is to build a user-friendly interface so that users can conduct the NMA based on TTE outcomes conveniently. The interface of the homepage is shown in Figure 1. In addition to enabling users to run NMA models, we also offer additional functions to assist with data preparation before running NMA and model selection afterward. The application consists of six modules, which can be easily accessed through the navigation bar: “Home Page,” “Data Page,” “AD-Based NMA,” “IPD-Based NMA,” “Application Output,” and “User Manual.” Some modules contain subsections that provide additional functionalities.

- “Data Page” module: This module allows users to upload their own data, transform IPD to AD, do the PH assumption tests, and generate network plots. Three kinds of PH assumption tests are available: Schoenfeld residual plot, Log–Log plot, and Grambsch–Therneau test. For the Network plot, users can create customized network plots by modifying design elements such as line color and background.
- “AD-Based NMA” module: This module consists of three sections, each corresponding to a different AD-based NMA model. Within each section, the application provides multiple subsections to accommodate various parameter settings, using frequentist or Bayesian frameworks, as well as using random-effects or fixed-effect models. This structure makes it easier for users to compare the results.
- “IPD-Based NMA” module: This module follows a similar structure to “AD-Based NMA.”
- “Application Output” module: In this module, users can quickly get familiar with the process and tips of model selection according to the notes provided. Additionally, users can export results in batches for further analysis here.

Most operations of this app can be simply finished by “point-and-click” on the web browser. Provided example data can help users quickly get in touch with the functions in this app. All generated results will be shown in texts, tables, or figures, which can be exported and downloaded. In Section 4, we provide an illustrative example of how to use this tool step-by-step.

## 4 An illustrative example

To illustrate the capacities of NMAsurv, we reanalyzed one NMA based on survival data published in *BMC Cancer*.^36^ This NMA aimed to compare the efficacy, safety, and effects on quality of life of different anaplastic lymphoma kinase (ALK)-inhibitors for global and Asian patients with advanced ALK-positive non-small-cell lung cancer (NSCLC). We reanalyzed the results of OS of first-line treatments on global patients. Authors included six RCTs (ALEX, CROWN, ALTA-1L, eXalt 3, PROFILE 1014, and ASCEND-4) with seven treatments (crizotinib, alectinib, lorlatinib, brigatinib, ensartinib, chemotherapy, and ceritinib). In this illustrative example, we used the same data as in the referenced NMA study for all analyses, which were the reconstructed pseudo-IPD based on the algorithm of Guyot et al.^19^ We presented only the FP model for AD-based NMA and the Cox PH model for IPD-based NMA, as the procedures for running other models are quite similar. We will go through this example based on the following steps: (1) uploading IPD; (2) transforming IPD to AD; (3) doing the PH assumption test; (4) creating the network plot; (5) running the FP model; (6) running the Cox PH model; and (7) generating a report. Please note that the steps outlined here apply only to this example; standardized procedures for conducting an NMA study can be found elsewhere.^1^ A detailed description of the steps for running all models can be found in the user manual. All data used in this illustrative example can be found in Supplementary Material 3.

**Figure 2:**
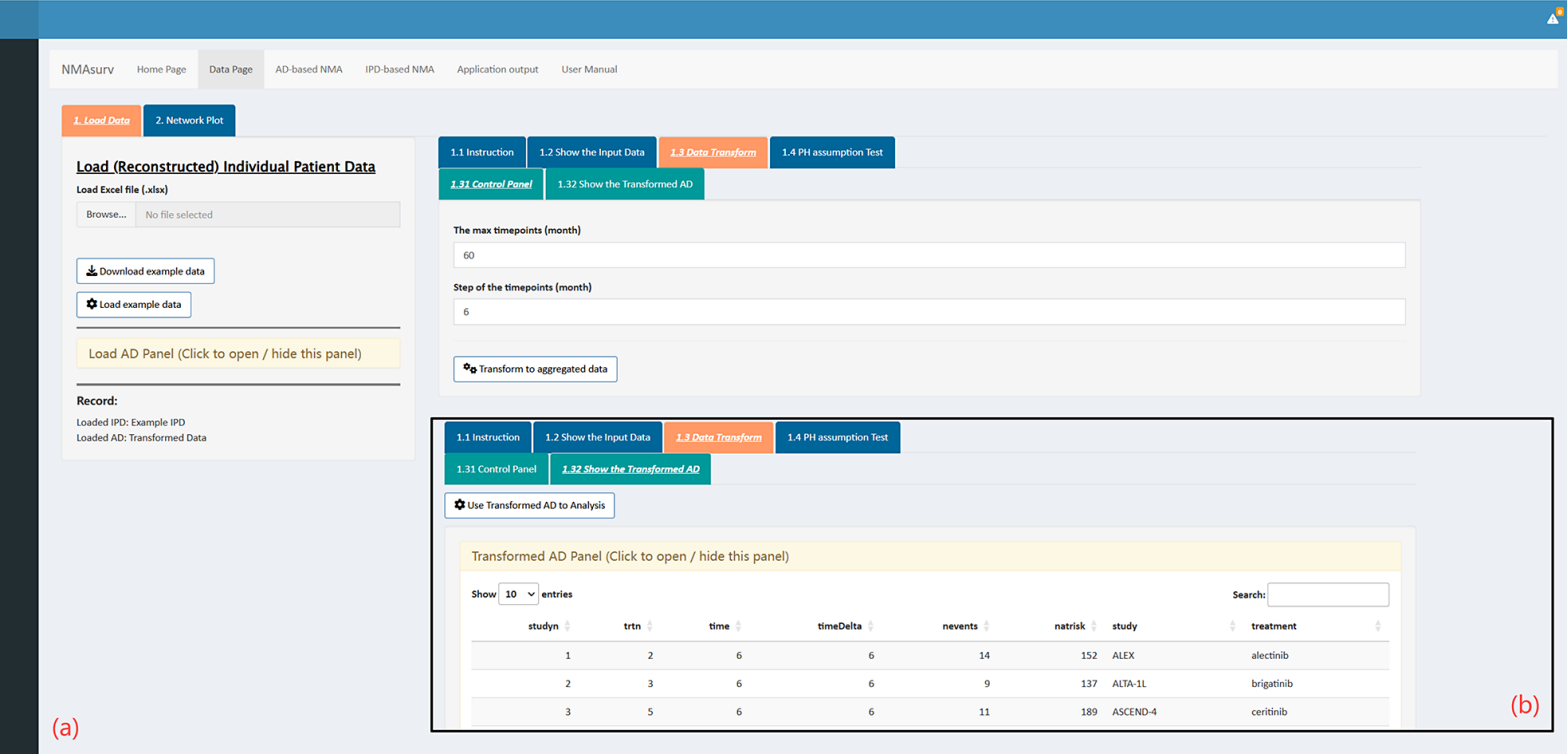
The interface of the “Data Transform” panel.

Uploading IPD is a compulsory step and should be the first step when using this app. Since NMAsurv is constructed based on the reconstructed pseudo-IPD, we can directly use the reconstructed pseudo-IPD here. To begin, navigate to the “Data Page” module and locate the “Load Data” section. A summary of how to import data and details on the required data format can be found in the “Instruction” panel. After loading the IPD, tables displaying summarized information about the imported data are available in the “Show the Input Data” panel. To run AD-based NMA models, AD is needed. Thus, transforming the IPD to AD in the “Data Transform” panel is the second step. Here, we should set “The max timepoint” and the “Step of the timepoint.” Typically, “The max timepoint” refers to the longest start timepoint in the AD, while “Step of the timepoint” refers to the interval between start timepoints. In this NSCLC-ALK network, since the longest follow-up time is approximately 65 months (PROFILE 1014, chemotherapy), we set “The max timepoint” to 60 and “Step of the timepoint” to 6. After clicking the “Transform to AD” button, followed by the “Use Transformed AD to Analysis” button, the AD is successfully generated and ready for analysis. (as shown in Figure 2a,b). In addition to using transformed AD as we did in this example, users can also choose to import AD. Before performing model analysis, the data source used in the analysis can be verified through the “Record” panel in the sidebar.

**Figure 3:**
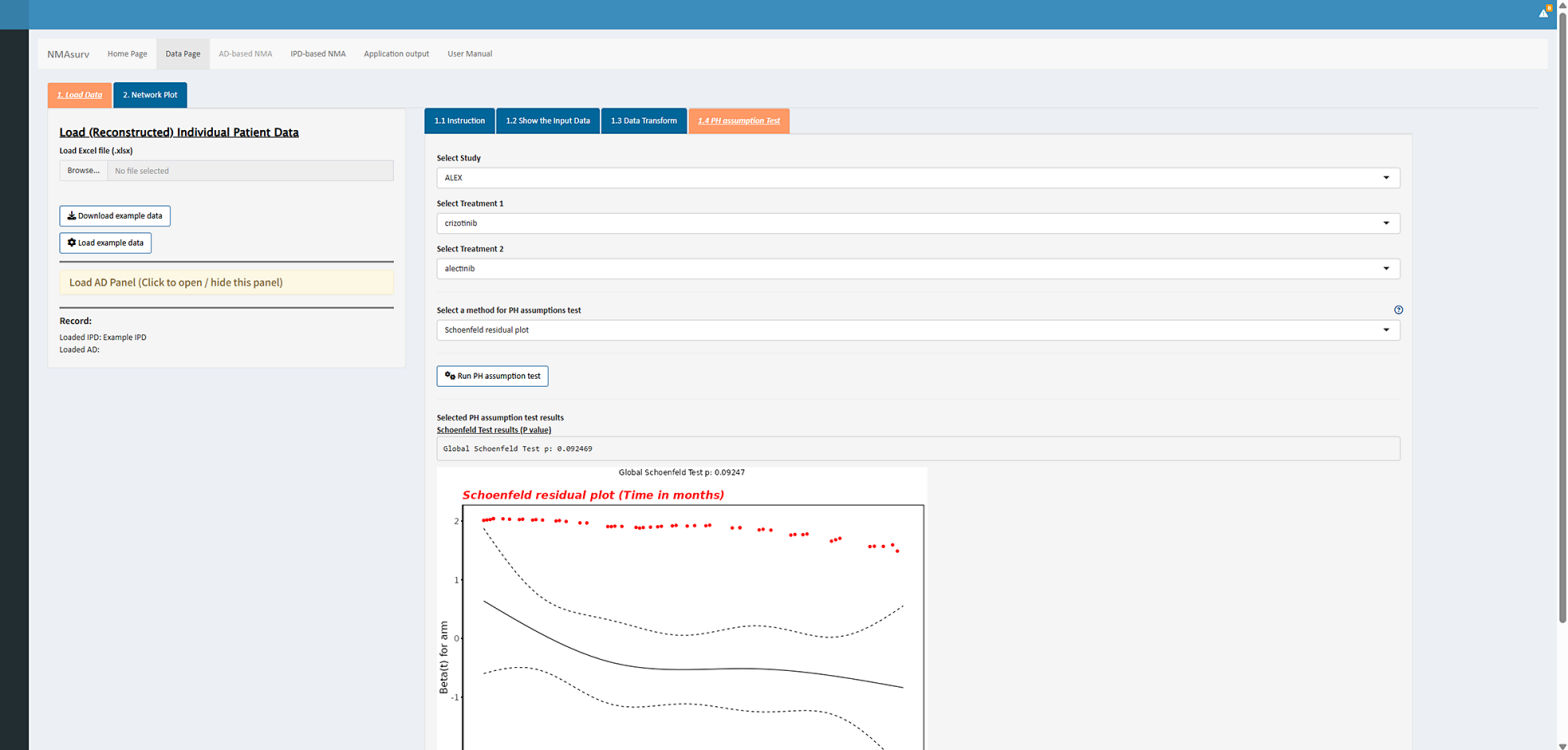
The interface of the “PH assumption test” panel.

The PH assumption test is the third step in our illustrative example, and it is a crucial step in TTE-based NMA. Rich studies on the PH assumption test can be found elsewhere.^37^^,^ ^38^ In the “PH assumption test” panel, we can select the studies and treatments to run the tests. Three PH assumption test methods are available, which can be chosen through the drop-down selection box. An example of Schoenfeld’s residual plot for ALEX is shown in Figure 3. All generated results for the NSCLC-ALK network are provided in Supplementary Material 2. According to the results, the Cox PH model is enough since the PH assumption holds. However, to demonstrate the functions in AD-based NMA, we still ran the FP model.

**Figure 4:**
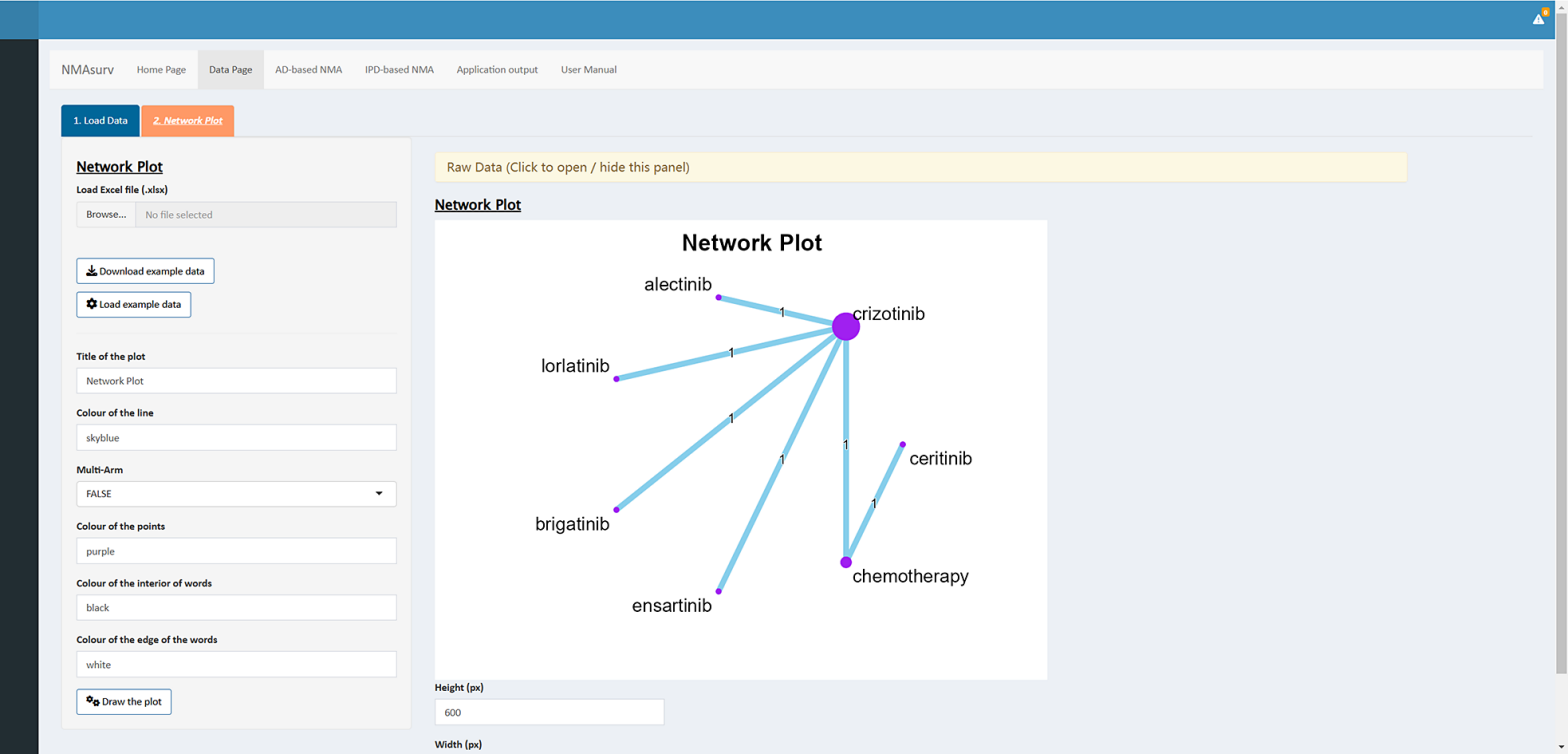
The interface of the “Network Plot” panel.

The fourth step involves creating the network plot. Since the data format for this step differs from that of IPD and AD, new data should be imported here. The data can be viewed in the table displayed in the main panel, as shown in Figure 4. A variety of customization options are available in the sidebar panel, including “the title of the plot,” “color of the line,” “multi-arm or not,” “color of the multi-arm area,” “color of the points,” “color of the interior of words,” and “color of the edge of words.” After clicking the button to draw the plot, the final network plot is displayed in the main panel (as shown in Figure 4).

**Figure 5:**
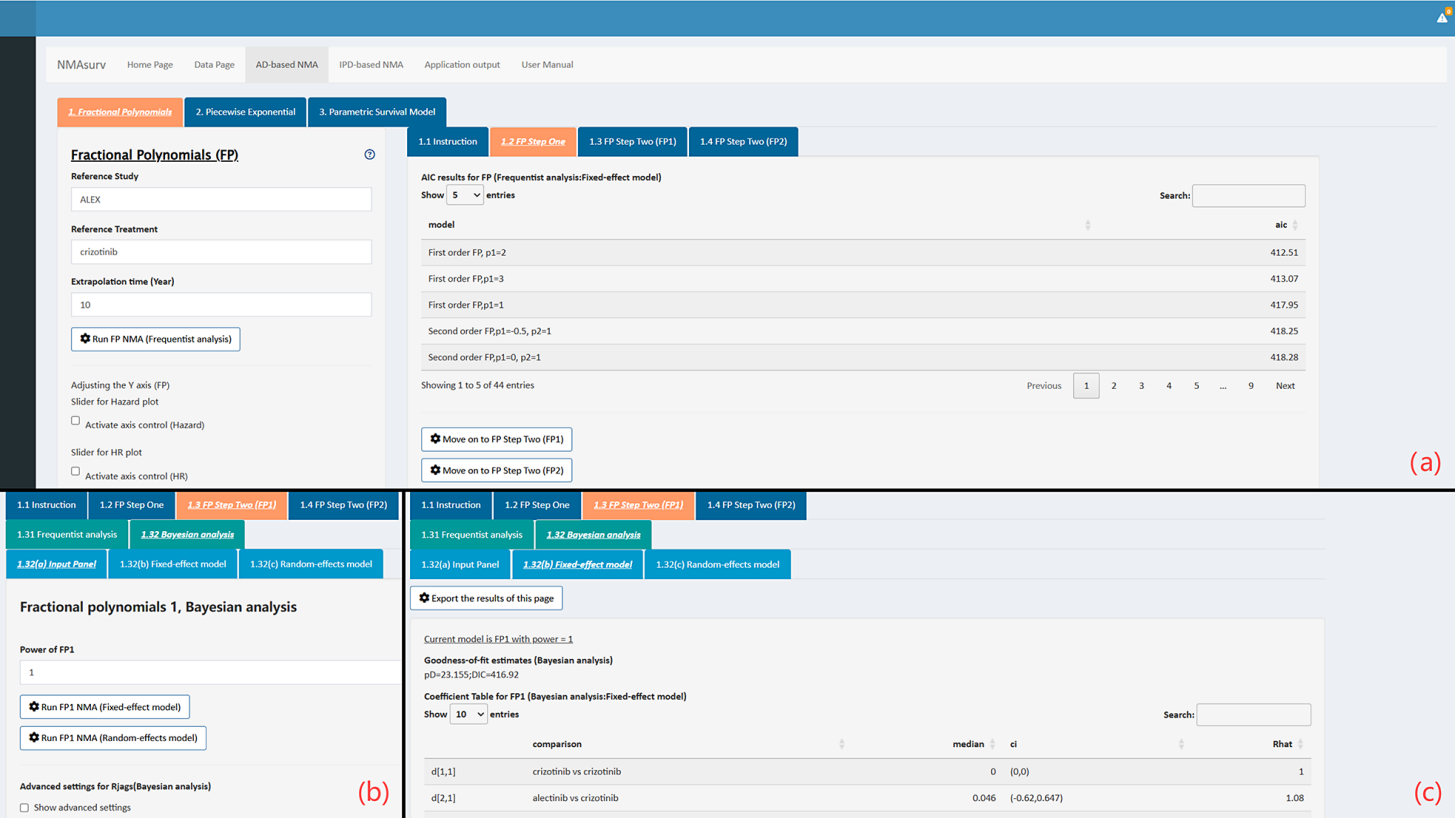
The interface of the panels in the FP model.

In the fifth step of this example, we ran the FP model to show a common procedure for running AD-based NMA models. First, we set the “Reference Study,” “Reference Treatment,” and “Extrapolation Time” through the text input box. In the NSCLC-ALK network, we selected “ALEX” as the reference study, “crizotinib” as the reference treatment, and 10 years as the extrapolation time. By clicking the “Run FP NMA (Frequentist analysis)” button, we obtained the step-one results for all FP models (as shown in Figure 5a). Then, we selected the model with the best statistical performance to run in the “FP Step Two (FP1)” panel. In this illustrative example, the FP1 models with powers of two, three, and one ranked top three. We finally selected the FP1 model with a power of one since overfitting was observed in subsequent runs of the other two models (more details can be found in the user manual of this app). A quick overview of the “Input Panel” and the results of Bayesian analysis using the fixed-effect model are shown in Figure 5b,c. More detailed results can be found in Supplementary Material 2. Based on the survival plot and the HR plot, we concluded that lorlatinib had the highest survival rate before the 45th month, while alectinib took the lead after the 45th month. The “Rhat” values in the coefficient table for Bayesian analysis showed that the model converges well. Additionally, parameter estimates from the frequentist and Bayesian analyses were highly consistent, indicating the robustness of the results. To illustrate parameter interpretation, we took alectinib versus crizotinib in the frequentist analysis as an example. The two parameters were 0.088 and −0.021, which indicated that the log HR can be written in the following formula: 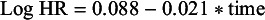 . For the fixed-effect model, NMAsurv provides downloads of extrapolated HRs.

**Figure 6:**
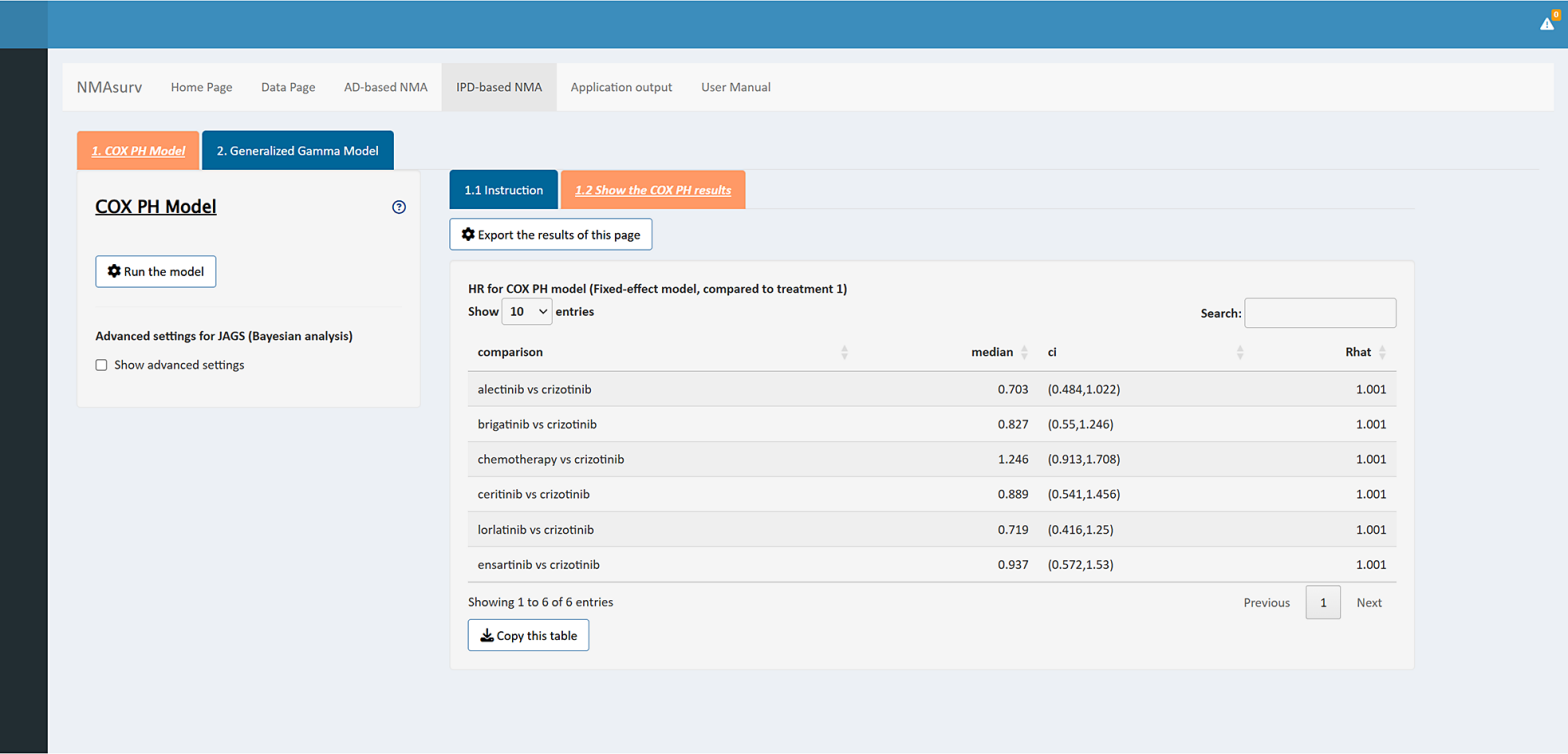
The interface of the panels in the Cox PH model.

The sixth step of this illustrative example involves running the Cox PH mode. The results can be easily obtained by clicking the button to run the model. HRs compared to the reference treatment are displayed in the main panel (as shown in Figure 6). Detailed information can be found in Supplementary Material 2. According to the results, all ALK inhibitors demonstrated HRs less than 1; however, their 95% confidence intervals (CIs) crossed 1. This suggested that, within this NSCLC-ALK network, the advantages of ALK inhibitors over crizotinib were not statistically significant. These findings might be different from the results obtained from other methods like “gemtc” or “netmeta” in R. A potential explanation is that the method used in this app takes the IPD into consideration, while “gemtc” and “netmeta” used RCT reported HRs with 95% CIs only.^13^

**Figure 7:**
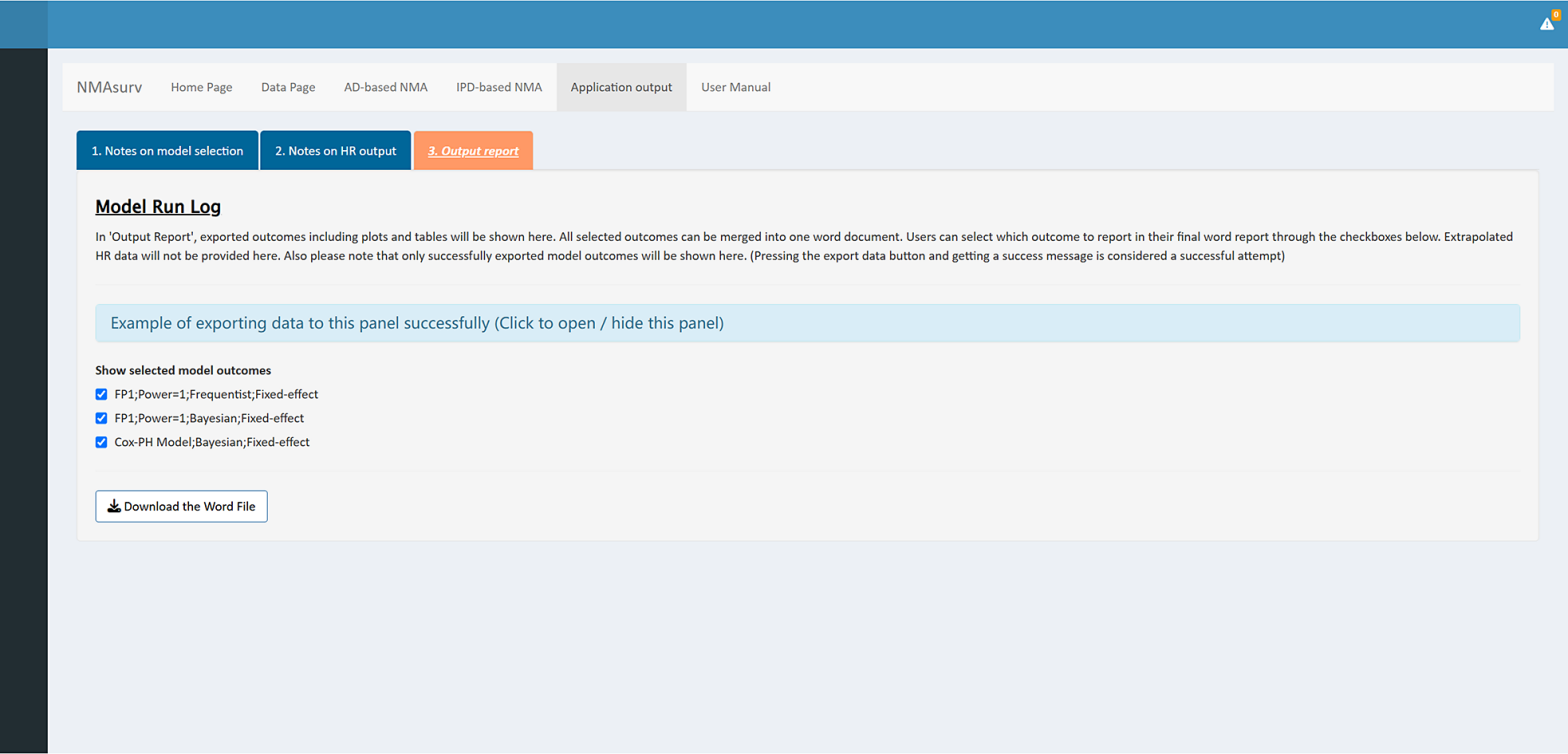
The interface of the “Output report” panel.

The final step of this example is to generate a report with selected results. NMAsurv provides an “Output Report” panel that enables users to export the results in batches. By clicking the “Export the results of this page” buttons as illustrated in Figures 5c and 6, the names of the selected model outcomes are displayed as checkboxes as shown in Figure 7. Then, we selected the desired outcomes by checking these boxes and then generated a report by clicking the “Download the Word File” button. The report included coefficient tables, survival plots, hazard plots, and HR plots, providing a detailed summary of the analyses.

## 5 Discussion

In this article, we introduced NMAsurv, a newly developed R Shiny tool designed for NMA based on survival data. With the emergence of innovative therapies, NMA, especially NMA under non-PH assumption, is becoming increasingly important in medical research. Flexible modeling techniques can be good alternatives to the traditional Cox PH model when the PH assumption is violated. However, methodologies of these methods are often complex and require users to have good programming skills. Shiny in R is a web-based interactive tool that enables users to run R functions using their own data without programming. Thus, users who have little knowledge of R programming or even non-R users can conduct NMA for TTE data through our tool. Moreover, NMAsurv offers a comprehensive set of results that cater to the needs of different users. The plotted survival curves provide clear insights for researchers assessing the long-term survival benefit differences between treatments, while the extrapolated HRs, available for download, support researchers conducting subsequent cost-effectiveness analyses.

It is important to highlight that NMA based on TTE outcomes can be directly established using trial-specific HRs,^39^^,^ ^40^ which relies on the PH assumption. Typically, these HRs are derived from Cox PH models developed by statistical analysts working on the trials. Researchers can calculate the logarithm of these HRs along with their SEs, which can then be input into statistical software such as R, Stata, and Gemtc to compute relative log HRs. However, we did not include this method in NMAsurv for two reasons: (1) it is already well supported by numerous tools that offer simple and comprehensive tutorials, and (2) it does not require IPD or AD, making it less aligned with the focus of NMAsurv on TTE outcomes. Instead, in NMAsurv, we adopted an alternative approach to conducting NMA under the PH assumption. Specifically, we applied the two-step, IPD-based approach developed by Freeman et al.^7^ We provide this function to enable users to directly analyze imported data when they determine that the PH assumption holds in their studies, thereby eliminating the need to switch to alternative software or reformat data for analysis.

Five models were included in NMAsurv, namely the Cox PH model, FP model, PWE model, PSM, and generalized gamma model, which are commonly used and well developed.^7^^,^ ^10^ Other existing models, such as cure models, the RP model, and the RMST model, were excluded from the current version of NMAsurv due to practical constraints. The current practice of constructing cure models, including the mixture cure model and non-mixture cure model, is to use Stan, which is a probabilistic programming language for statistical inference written in C++.^10^^,^ ^41^ In addition, cure models are the best choice when the cure is clinically realistic or can be estimated.^10^ The results will be biased if the survival data are immature, or the cure is estimable for the treatment group but not for the reference group. The RP model is a highly flexible approach that accommodates extreme changes in hazard functions over time. It is considered more adaptable and robust than FP models, as it reduces the likelihood of undesirable end effects and requires fewer iterations for convergence.^27^ However, its complex and data-specific coding structure and increased computational burden present challenges for the implementation of simple “point-and-click” operations.^7^^,^ ^27^ The RMST model is an alternative to the HR when there is evidence of non-PH. However, a key limitation of this approach is that it does not support extrapolation, nor does it provide estimates of survival rates.^7^ Additionally, like the RP model, its data-specific coding structure makes it difficult to be included in an application. Given these challenges, our tool focuses on models that are both well-developed and feasible for implementation in a user-friendly interface. Nonetheless, researchers are encouraged to try and find more modeling approaches than those provided by NMAsurv based on the characteristics of their data and the features of different models. Further information on the methodologies and corresponding codes is available elsewhere.^1^^,^ ^7^^,^ ^10^^,^ ^22^^,^ ^23^^,^ ^25^^,^ ^29^

Despite NMAsurv making the model construction in NMA research easier to realize, it has several limitations. First, this study was conducted based on reconstructed pseudo-IPD without taking individual patient characteristics into consideration, which is a popular way in NMA for TTE data. If researchers have true IPD with individual patient characteristics, we highly recommend them to try additional IPD-based methods. For two-study comparisons, methods such as STC and MAIC can be used. For larger treatment networks, multilevel network meta-regression is a suitable approach.^16^^,^ ^17^^,^ ^42^ Second, this tool only supports NMA methods that are well developed. While cure models, RP models, and RMST models were excluded from this version, these models may be included in future updates. Additionally, the random-effects model for all parameters in FP, whose methodology is still controversial, was not included in our tool. Third, the rank plot, which can be an important output in NMA, is not included in our tool. This is because the current treatment ranking code relies on the WinBUGS-specific function “ranked,” which is not available in JAGS. The reason we did not use WinBUGS is that codes based on WinBUGS cannot be deployed on the server (shinyapps.io, Linux). Fourth, although NMAsurv provides extrapolated HRs for download, which can be used in CEA, users should pay attention when using these estimates, especially when their research data are immature.^43^^,^ ^44^ Relying on these potentially imprecise estimates may lead to research bias. While some methods have been applied in published studies to reduce this bias,^45^^–^ ^47^ we strongly recommend that researchers perform comprehensive sensitivity analyses to thoroughly assess the impact of extrapolation. Finally, our tool is designed to generate statistical results and plots rather than explicitly recommending a specific model for users to choose. This serves as an important reminder: model selection is a complex process, and relying solely on statistical outcomes is not sufficient to make an informed decision. It is also crucial to assess the presence of overfitting by visual inspection by users themselves. To ensure transparency and reliability, following a standardized process for model selection is highly recommended.^43^ Although NMAsurv does not directly provide a conclusion for model selection, it enables users to export results in batches and offers several helpful notes to guide and inform the model selection process.

Future extensions of NMAsurv will include two directions. First, adding a survival data extrapolation function will be beneficial to the output of IPD-based NMA. Currently, only HRs or relative treatment effects are reported in IPD-based methods, while the report of survival plots or HR plots needs the extrapolation of survival data. Second, integrating more IPD-based methods will be the main direction. Taking covariates into consideration and including methods like the RP, RMST models will make this tool better.^7^^,^ ^48^ Future studies may focus on the simplification of algorithms in these complex models.

## Supporting information

Shao et al. supplementary material Shao et al. supplementary material

## Data Availability

The software in this paper is available at https://psurvivala.shinyapps.io/NMAsurv/. The source codes of the software are freely available on GitHub (https://github.com/TaihangShao/NMAsurv), and the data presented in this paper can be found in the Supplementary Material.

## Supplementary material

To view supplementary material for this article, please visit http://doi.org/10.1017/rsm.2025.10020.
