# Supplementary material for: NMAsurv: An R Shiny application for network meta-analysis based on survival data
Source: Res Synth Methods. 2025 Jul 10;16(6):1042–56. doi: 10.1017/rsm.2025.10020 (PMC12657653; doi:10.1017/rsm.2025.10020)
Supplement: Shao et al. supplementary material [file S1759287925100203sup001.zip › supporting information/supporting information 1.docx]

**User Manual: Platform for Network Meta-Analysis based on Survival data**

April 14th, 2025

**Title** Platform for **N**etwork **M**eta-**A**nalysis based on **Surv**ival data (NMAsurv)

**Date** 2024-8-23

**Author** Taihang Shao; Mingye Zhao; Fenghao Shi; Mingjun Rui and Wenxi Tang.

**Bugs Report and Consultation** travis_shao@outlook.com

**Description** This user-friendly application can be used to conduct network meta-analysis based on reconstructed survival data.

**Constructed Based on R (4.3.1)**

**License** GPL-2

**R Packages Needed**

| **Package** | **Verison** | **Package** | **Verison** | **Package** | **Verison** |
| --- | --- | --- | --- | --- | --- |
| broom | 1.0.7 | markdown | 1.13 | shinyBS | 0.61.1 |
| clipr | 0.8.0 | markdownInput | 0.1.2 | shinycssloaders | 1.1.0 |
| discSurv | 2.0.0 | MatrixModels | 0.5-3 | shinydashboard | 0.7.2 |
| doBy | 4.6.24 | metafor | 4.6-0 | shinydashboardPlus | 2.0.5 |
| dplyr | 1.1.4 | netmeta | 2.9-0 | shinyhelper | 0.3.2 |
| DT | 0.33 | officer | 0.6.7 | shinyjs | 2.1.0 |
| flexsurv | 2.3.2 | R.utils | 2.12.3 | shinymanager | 1.0.410 |
| flextable | 0.9.7 | R2jags | 0.8-9 | shinytitle | 0.1.0 |
| ggmcmc | 1.5.1.1 | rclipboard | 0.2.1 | shinyWidgets | 0.8.7 |
| ggplot2 | 3.5.1 | readxl | 1.4.3 | slickR | 0.6.0 |
| grid | 4.4.0 | rhandsontable | 0.3.8 | survHE | 2.0.2 |
| gridExtra | 2.3 | rmarkdown | 2.29 | survival | 3.8-3 |
| heemod | 1.0.2 | scales | 1.3.0 | survminer | 0.5.0 |
| knitr | 1.49 | shiny | 1.10.0 | tidyverse | 2.0.0 |
| lme4 | 1.1-36 | shinyalert | 3.1.0 | writexl | 1.5.1 |
| magrittr | 2.0.3 |  |  |  |  |

**Contents**

[1 Introduction 3](#_Toc189575086)

[1.1 Opening this APP 3](#_Toc189575087)

[1.2 Usage and potential users of this APP 3](#_Toc189575088)

[1.3 How to use this APP 3](#_Toc189575089)

[2 Example Data Used in This APP 4](#_Toc189575090)

[2.1 Main example data (NSCLC-ALK Network) 4](#_Toc189575091)

[2.2 Data of the Melanoma Network (Individual Patient Data) 4](#_Toc189575092)

[2.3 Data of the NSCLC-Che Network (Aggregated Data) 4](#_Toc189575093)

[2.4 Data of the NSCLC-PDX Network (Network Plot) 5](#_Toc189575094)

[3 Network Meta-Analysis of Survival data (NMAsurv) 6](#_Toc189575095)

[3.1 Introduction of NMAsurv 6](#_Toc189575096)

[3.2 Get started with NMAsurv 8](#_Toc189575097)

[3.3 Data Page 8](#_Toc189575098)

[3.31 Data format and Show the Input Data Panel 9](#_Toc189575099)

[3.32 Data transform 11](#_Toc189575100)

[3.33 PH assumption tests 13](#_Toc189575101)

[3.34 Network Plot 16](#_Toc189575102)

[3.4 AD-based NMA 20](#_Toc189575103)

[3.4.1 Fractional polynomials 20](#_Toc189575104)

[3.4.2 Piecewise Exponential Model 27](#_Toc189575105)

[3.4.3 Parametric survival model 30](#_Toc189575106)

[3.5 IPD-based NMA 32](#_Toc189575107)

[3.5.1 COX PH model 32](#_Toc189575108)

[3.5.2 Generalized Gamma Model 35](#_Toc189575109)

[3.6 Application Output 38](#_Toc189575110)

[3.6.1 Notes on model selection 38](#_Toc189575111)

[3.6.2 Notes on HRs output 41](#_Toc189575112)

[3.6.3 Instruction on “Output Report” panel 42](#_Toc189575113)

[4 Tips for users when using this APP 43](#_Toc189575114)

[5 Reference 44](#_Toc189575115)

[6 Appendix 46](#_Toc189575116)

[6.1 JAGS codes 46](#_Toc189575117)

[6.2 Output report example for NSCLC-ALK network 55](#_Toc189575127)

# Introduction

## 1.1 Opening this APP

This Shiny APP (NMAsurv) is constructed based on R (4.3.1) and has been deployed on the **shinyapps.io**. The URL of this shiny app is: https://psurvivala.shinyapps.io/NMAsurv/. Please note that 900 seconds of inactivity before a browser connection to a worker process is idle and the APP will be closed.

## 1.2 Usage and potential users of this APP

Nowadays, decision making for anti-cancer drugs usually requires a lifetime projection including survival benefit and cost. Meanwhile, healthcare decision-making also requires comparisons of all relevant competing interventions. However, the individual patient data (IPD) is often unobtainable. Thus, reconstructed pseudo-IPD is used by researchers instead. In addition, the realization of some flexible methods needs users to have a certain level of programming skills. This APP can be used to conduct network meta-analysis (NMA) based on survival data. In this APP, we provided several methods including Aggregated data (AD)-based NMA and IPD-based NMA. If users have survival data with covariates, we recommend the users to conduct further analyses like multilevel network meta-regression or considering treatment-by-covariates interactions.

Potential users for this APP include investigators who have a good grasp of the principles of survival analysis and NMA, and understand the strengths and weaknesses of these methods, but do not have the time, statistical background or programming expertise to conduct these methods themselves. Potential users can also be researchers who are majoring in health economics but do not have a deep understanding of evidence-based medicine; or clinicians who have extensive clinical experience but do not have a background in statistical methodologies and programming.

## 1.3 How to use this APP

This User Manual will introduce the properties of this APP and discusses the situations where it may or may not be suitable. Figures will be provided to help users to learn how to use this APP quickly. Besides, description of the methods used as well as their basic codes and functions will be provided in summary here. If users are not satisfied with the results, they may use provided codes and description to generate results in R themselves. Please note that all data of examples provided in this APP can be obtained from published articles. This APP does not make any statement about the appropriateness of particular therapies which are used in examples.

# Example Data Used in This APP

## 2.1 Main example data (NSCLC-ALK Network)

The main example data was extracted from an NMA for survival data published in BMC Cancer^1^. This NMA aimed to compare the efficacy, safety and effects on quality of life of different anaplastic lymphoma kinase (ALK)-inhibitors for global and Asian patients with advanced ALK-positive non-small-cell lung cancer (NSCLC). For this example data, we reanalyzed the result of OS of first-line treatments on global patients. Authors included six RCTs (ALEX, CROWN, ALTA-1L, eXalt 3, PROFILE 1014, ASCEND-4) with seven treatments (crizotinib, alectinib, lorlatinib, brigatinib, ensartinib, chemotherapy, ceritinib). In this illustrative example, we used the same data as this NMA study for all analysis, which was the reconstructed pseudo-IPD based on the algorithm of Guyot et.al. 2012^2^. Some basic information for this example data can be found below.

Table 2‑1 Basic information for example data

| Trial | Intervention | Total patients | HR for OS (95% CI) | Median follow-up time (Treatment Group) |
| --- | --- | --- | --- | --- |
| ALTA-1L | Brigatinib VS Crizotinib | 137 VS 138 | 0.81 (0.53–1.22) | 40.4 months |
| PROFILE 1014 | Crizotinib VS Pemetrexed + Cisplatin/Carboplatin | 172 VS 171 | 0.76 (0.55–1.05) | 46 months |
| CROWN | Lorlatinib VS Crizotinib | 149 VS 147 | 0.72 (0.41–1.25) | 18.3 months |
| ALEX | Alectinib (600 mg twice daily) VS Crizotinib | 152 VS 151 | 0.67 (0.46–0.98) | 48.2 months |
| eXalt3 | Ensartinib VS Crizotinib | 143 VS 147 | 0.91 (0.54–1.54) | 23.8 months |
| ASCEND-4 | Ceritinib VS Pemetrexed + Cisplatin/Carboplatin | 189 VS 187 | 0.73 (0.50–1.08) | 19.7 months |

## 2.2 Data of the Melanoma Network (Individual Patient Data)

This additional example IPD is extracted from a Melanoma Network. This data is the reconstructed IPD obtained from Freeman S.C. et.al.’s research^3^. And the original source is a NMA of therapies for previously untreated advanced BRAF-mutated melanoma^4^. The NMA identified 23 eligible articles reporting on thirteen phase II and phase III randomized controlled trials. The melanoma network consists of 3913 overall survival events from 6378 patients. In addition, the network includes 13 trials and 13 treatments: dacarbazine, tremelimumab, ipilimumab, dabrafenib, vemurafenib, nivolumab, pembrolizumab, ipilimumab + dacarbazine, dabrafenib + trametinib, vemurafenib + cobimetinib, nivolumab + ipilimumab, selumetinib + dacarbazine and ipilimumab + sargramostin.

## 2.3 Data of the NSCLC-Che Network (Aggregated Data)

This additional example AD is extracted from Anna Wiksten et.al^5^. The original source is a study published by Jansen^6^. The example consists of survival data from 7 studies and 4 treatments (docetaxel, pemetrexed, gefitinib, and best supportive care). Any question on how to get the aggregated data please refer to Jansen’s study where a detailed description can be found.

## 2.4 Data of the NSCLC-PDX Network (Network Plot)

This additional example data for network plot is obtained from a published NMA comparing the effectiveness of PDX in NSCLC^7^. OS data which included nine trials (10 treatments) was chosen. 10 treatments include chemotherapy, camrelizumab + chemotherapy, nivolumab + ipilimumab, nivolumab + ipilimumab + chemotherapy, atezolizumab + chemotherapy, bevacizumab + chemotherapy, atezolizumab + bevacizumab + chemotherapy, pembrolizumab + chemotherapy, sintilimab + chemotherapy, and nivolumab + bevacizumab + chemotherapy.

# Network Meta-Analysis of Survival data (NMAsurv)

## 3.1 Introduction of NMAsurv

This tool (NMAsurv) can be used to conduct network meta-analysis (NMA) based on reconstructed survival data. In this APP, we provided several methods including Aggregated data (AD)-based NMA and Individual patient data (IPD)-based NMA. Please note that not all existing NMA methods are included in this app, so please be careful when using this APP. Methods considered in this APP include Fractional polynomials (FP), Piecewise exponential model (PWE), Parametric survival model, Cox proportional hazard (PH) Model and Generalized Gamma Model. A summary of the five implemented methods along with their respective data and model assumptions is provided in Table 3-1. Some useful functions like data transform from IPD to Aggregated data, Network plot and PH assumption tests. Some other flexible methods like Restricted mean survival time (RMST) and Royston-Parmar models (RP) are not included in this APP and may be available in the future.

Typically, like survival extrapolation, users can compare the performance of different models through the goodness-of-fit (GOF) statistics. In this tool, the GOF statistics including AIC and Deviance information criterion (DIC) are provided. AIC is described by the following formula^8^:

$$AIC=-2logL+2k$$

*L* refers to the likelihood of the model and *k* refers to the number of parameters. DIC is described by the following formula^6,9^:

$$DIC=\overset{\_}{D}+pD$$

$$pD=\overset{\_}{D}-\overset{\wedge}{D}$$

$\overset{\_}{D}$ is the posterior mean residual deviance, pD is the ‘effective number of parameters’ and $\overset{\wedge}{D}$ is the deviance evaluated at the posterior mean of the model parameters. Please note that sometimes AIC and DIC may not be applicable when comparing different types of models. For AD-based methods, hazard plots and survival plots can be drawn through which users can conduct the visual inspection. For IPD-based methods, only treatment effects or HRs are provided. But users can still draw hazard plots and survival plots themselves based on the provided parameters and their own extrapolation results. Anyway, the final model choice should be based on the users’ final usage and several sensitivity analyses. Although in this APP, the results or conclusions on which model performs best will not be provided, users can batch export the results (images and tables) of their selected models into a Word document to facilitate their comparison of results or report writing. Users can also benefit a lot from many published studies including frameworks and suggestions on selecting NMA models^10,11^. In addition, we provide some tricks to help the researchers to make the model selection more rigorous and comprehensive. Details can be found in section 3.6.1.

Table 3‑1 A summary of the five implemented methods and their data/model assumptions

| Data availability / approaches | FP model | PWE model | PSM | Cox PH model | Generalized Gamma model |
| --- | --- | --- | --- | --- | --- |
| One-step IPD | # | # |  |  |  |
| Two-step IPD |  |  | $ |  |  |
| One-step AD |  |  |  |  |  |
| Two-step AD |  |  |  |  |  |
| Frequentist |  |  | * |  |  |
| Bayesian |  |  |  |  |  |
| FE model |  |  |  |  |  |
| RE model |  |  |  | § |  |
| Notes: Green = implemented in the app; red = supported in the literature but not implemented; grey = not applicable.  Abbreviations: FP: fractional polynomial; PWE: piecewise exponential; PSM: parametric survival model; PH: proportional hazard; IPD: individual participant data; AD: aggregated data; FE: the fixed-effect; RE: the random-effects.  * PSM under the frequentist framework can only be realized through the two-step IPD approach  # Can refer to Freeman SC et.al. (2022) ^3^  $ Can refer to Cope S et.al. (2020) ^12^  § Can refer to Bowden J et.al. (2011) ^13^ | | | | | |

| 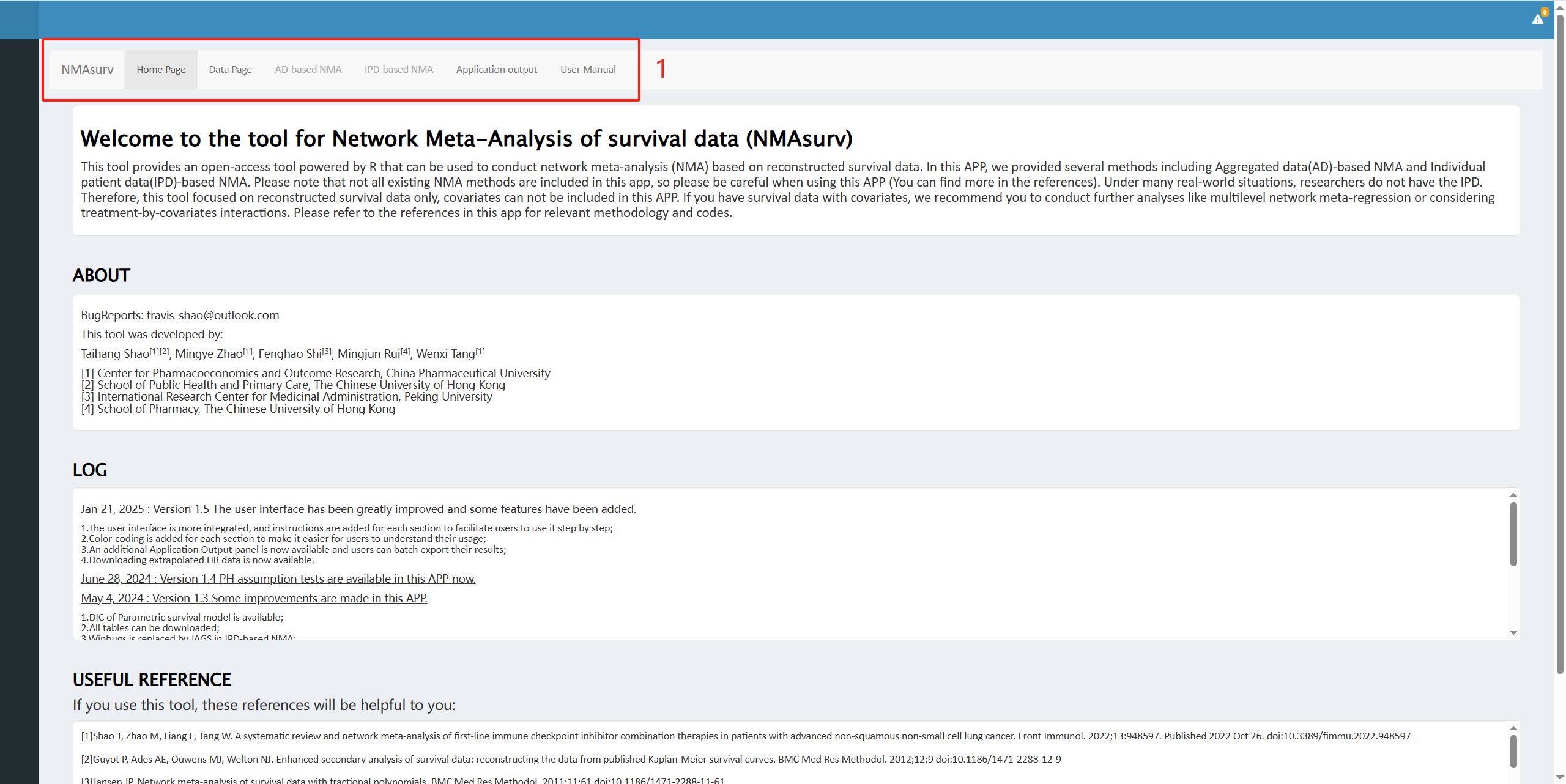 |
| --- |

Figure 3‑1 Dashboard of NMAsurv

## 3.2 Get started with NMAsurv

NMAsurv can be opened through the URL (https://psurvivala.shinyapps.io/NMAsurv/) directly. According to Figure 3-1, highlight position 1 is the navbar of this tool, through which users can switch to different modules easily. In this APP, totally six modules can be selected: “Home Page”, “Data Page”, “AD-based NMA”, “IPD-based NMA”, “Application output”, and “User Manual”. Before loading the data, two NMA modules are not available for users. Please note that users should have some basic knowledge of NMA based on survival data if they want to use this tool (e.g. know what NMA is and what survival data is). Thus, we provided references for some useful articles in the section “Useful Reference” on the homepage.

## 3.3 Data Page

For “Data Page” module, there are two main sections named “Load Data” and “Network Plot”. Four subsections can be found for “Load Data”: “Instruction”, “Show the Input Data”, “Data Transform”, and “PH assumption Test”. Before loading the data and moving on to the following modules, users are highly recommended to read the brief instruction in the “Instruction” section. To make users clear about the procedures during the data inputting, some useful information about the steps for data inputting are provided: (1) Users should load the (reconstructed) IPD first, and they can check the input data in the 'Show the Input Data' panel; (2) Users are highly recommended to read the notes on data format before moving on; (3) If the data is not imported, many functions will temporarily not be available for users; (4) After inputting the IPD, users can either transform the IPD to AD through the 'Data Transform' panel or load their own AD directly through the 'Load AD' panel; (5) Users should test the PH assumption through the 'PH assumption Test' panel before running the NMA models. A flow chat showed all steps for data inputting can be found below (Figure 3-2):


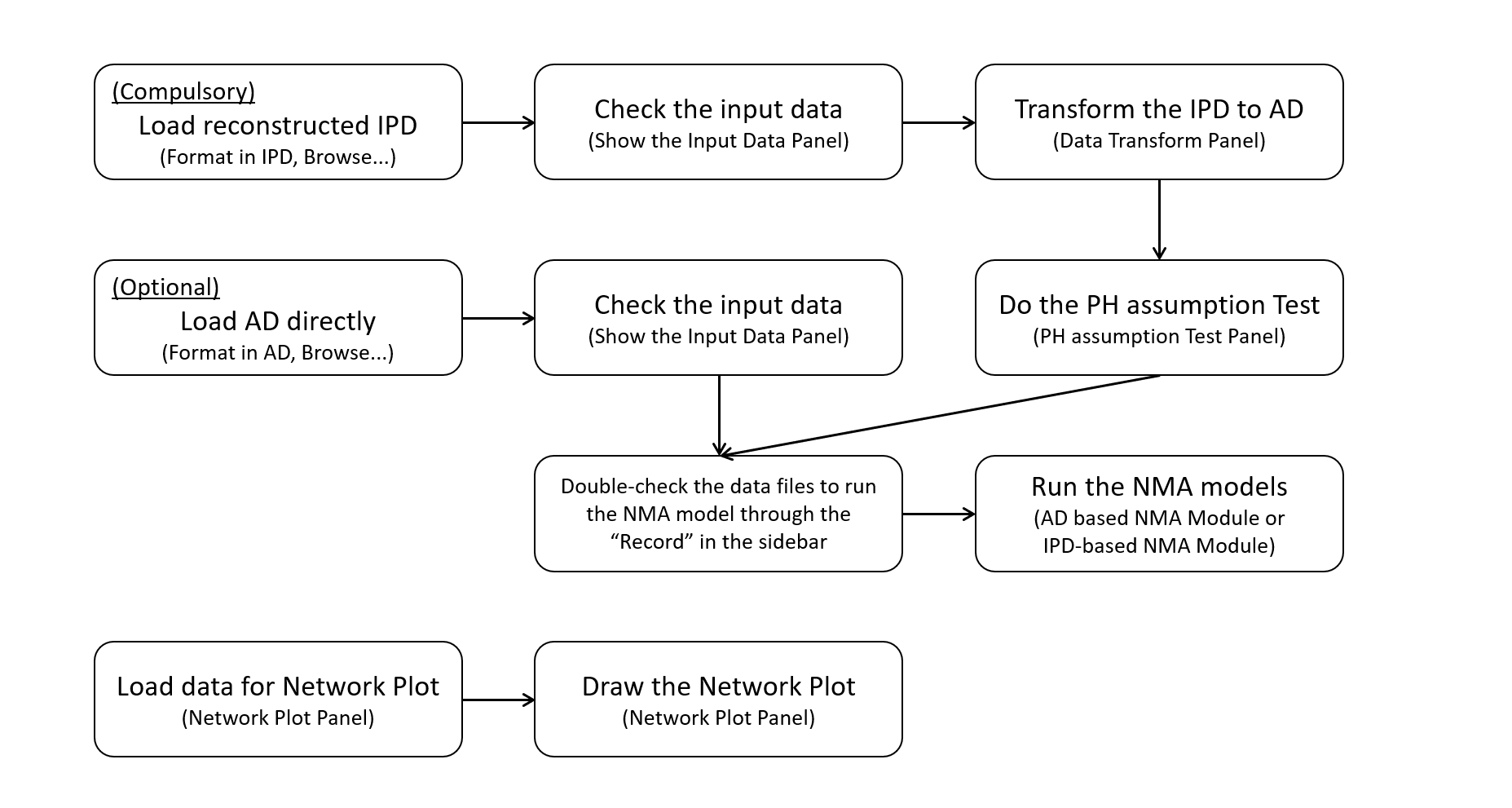


Figure 3‑2 Flow chart for data input

### 3.31 Data format and Show the Input Data Panel

Users should always pay more attention to the data format before loading the data. The example IPD (NSCLC-ALK network) is shown as Table 3-2. For the input IPD, it should include **seven** columns: n, patid, time, event, arm, study and treatment. **n** [num] is the ID of record; **patid** [num] is the ID of a patient in a trial; **time** [num] is the survival time of a patient (in months); **event** [num] refers to whether the patient is survival or dead; **arm** [num] refers to the arm of the trial; **study** [chr] refers to the study Name; **treatment** [chr] is the name of treatment. After uploading the data, NMAsurv will generate two tables to show the included treatments and their reference numbers. An example can be found as Table 3-3. The order of code arrangement is based on the order in which the target first appears. Typically, we use code 1 for the reference treatment and the reference study.

Table 3‑2 Example IPD

| n | patid | time | event | arm | study | treatment |
| --- | --- | --- | --- | --- | --- | --- |
| 1 | 1 | 1.5367 | 1 | 1 | ALEX | crizotinib |
| 2 | 2 | 2.2049 | 1 | 1 | ALEX | crizotinib |
| 3 | 3 | 2.8062 | 1 | 1 | ALEX | crizotinib |
|  | ...... | | | | | |
| 1838 | 169 | 65.9757 | 0 | 2 | PROFILE 1014 | chemotherapy |
| 1839 | 170 | 65.9757 | 0 | 2 | PROFILE 1014 | chemotherapy |
| 1840 | 171 | 66 | 0 | 2 | PROFILE 1014 | chemotherapy |

Table 3‑3 Included treatments and reference numbers

| Treatment_name | Treatment_code | Study_name | Study_code | Study_arm |
| --- | --- | --- | --- | --- |
| crizotinib | 1 | ALEX | 1 | 2 |
| ...... | | | |  |

The example AD (NSCLC-ALK network) is shown as Table 3-4. For the input AD, it should include **eight** columns: studyn, trtn, time, timeDelta, nevents, natrisk, study and treatment. **studyn** [num] is the reference number of specific study; **trtn** [num] is the reference number of specific treatment; **time** [num] refers the time of one specific observation (in months); **timeDelta** [num] refers to the duration of time between two consecutive observations; **nevents** [num] represents number of events; **natrisk** [num] refers to number at risk; **study** [chr] refers to the study name; **treatment** [chr] is the name of treatment.

Table 3‑4 Example aggregated data

| studyn | trtn | time | timeDelta | nevents | natrisk | study | treatment |
| --- | --- | --- | --- | --- | --- | --- | --- |
| 1 | 2 | 6 | 6 | 14 | 152 | ALEX | alectinib |
| 2 | 3 | 6 | 6 | 9 | 137 | ALTA-1L | brigatinib |
| 3 | 5 | 6 | 6 | 11 | 189 | ASCEND-4 | ceritinib |
| ...... | | | | | | | |
| 6 | 4 | 66 | 6 | 0 | 21 | PROFILE 1014 | chemotherapy |
| 1 | 1 | 60.735 | 0.735 | 0 | 11 | ALEX | crizotinib |
| 6 | 1 | 65.9388 | 5.9388 | 0 | 8 | PROFILE 1014 | crizotinib |


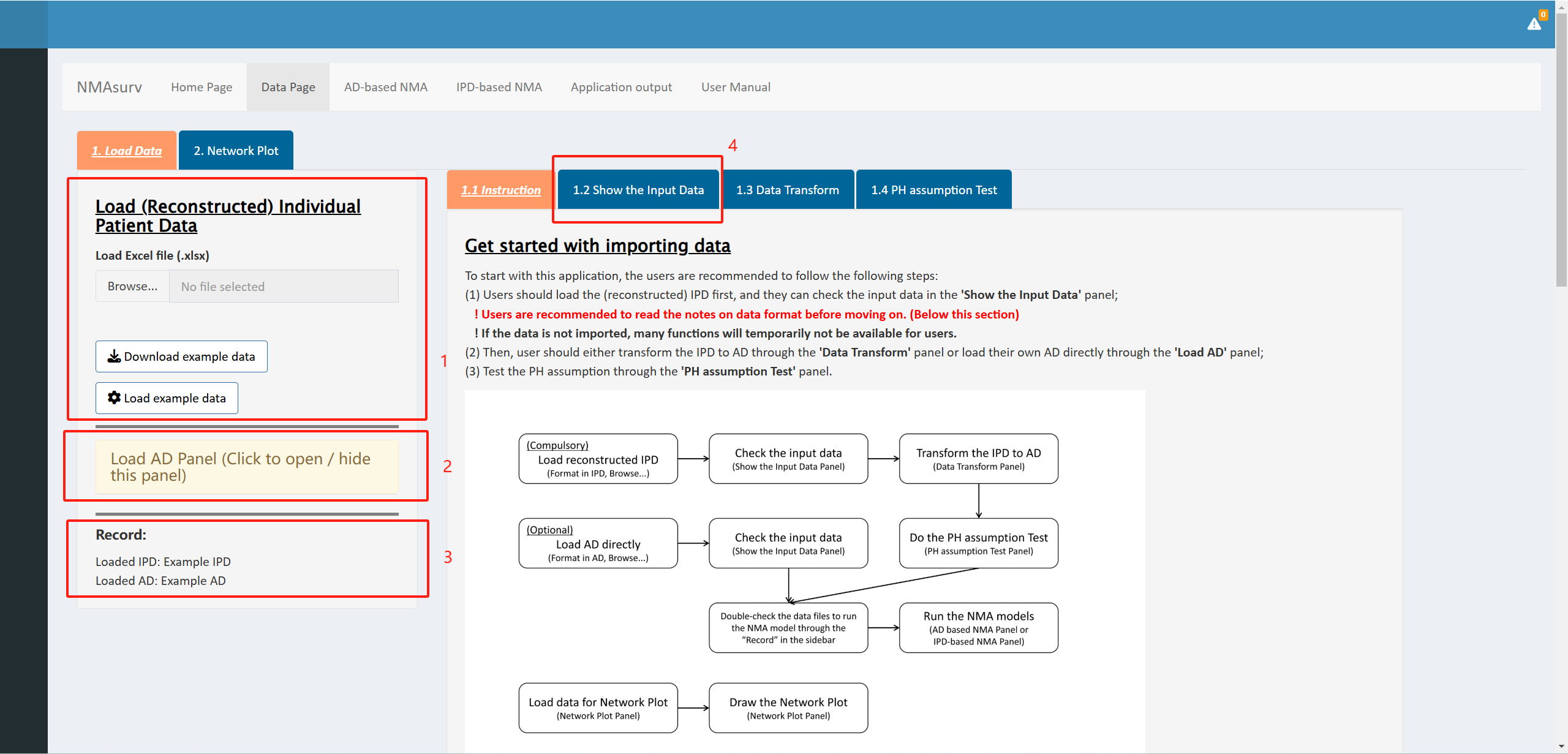


Figure 3‑3 Interface of the Load Data panel

In this APP, uploading the IPD (Figure 3-3, highlight 1) is the compulsory step, and the information of the inputted IPD will be shown in the “Show the Input Data” panel (Figure 3-3, highlight 3). Inputting AD is not a compulsory step since users can choose to use the “Data Transform” function to get the transformed AD. Therefore, it will be hidden in a CollapsePanel when launching the APP (Figure 3-3, highlight 2). After uploading the AD, the information of the inputted AD will also be shown in the “Show the Input Data” panel (Figure 3-3, highlight 3). At any time, users can check what data this APP will use to run the NMA models in the record panel which is shown in highlight 4 in Figure 3-3.

### 3.32 Data transform

This APP provide the function “Data transform”, which can allow users to transform the IPD data into aggregated data. According to Figure 3-4, users can use two input boxes which are shown in highlight position 1 to customize the aggregated data based on their own data. To be specific, **“The max timepoint”** refers to the longest start timepoint in the aggregated data. (In the NSCLC-ALK network, (1) we want to set the start timepoint sequence as “0, 6, 12, 18,~”; (2) and the longest follow-up time is nearly 65 months; thus, we input 60 here). **“Step of the timepoint”** refers to the step of the start timepoint sequence. (Similarly, the sequence of “0, 6, 12, 18,~”, the step is 6 here). “The start timepoint” means the start time of each time interval. (In the NSCLC-ALK network, 0 is the start time of the time interval of 0-6, …, 60 is the start time of the time interval of 60 to the end). After clicking the button (Figure 3-4, highlight 2), users can check the generated AD in the “Show the Transformed AD” panel. If users want to use the transformed AD to conduct the analysis, they should press the button (Figure 3-4, highlight 3) to input the transformed AD.

The detailed codes of data transform will not be provided here. The method we used is Anova Parameterization, which is quite similar to the method of SC Freeman et.al.^3^. The main idea is to calculate the total events and censors in each given time intervals based on the imported IPD. An example code for Anova Parameterization can be found below. Reported total events and censors in RCTs are highly recommended to verify and calibrate the generated AD.

anova_data <- function(timepoints, timepoints2, ref.study=1, df){

df2 <- survSplit(Surv(time, event) ~., data=df, cut=timepoints, episode ="timegroup")

df2$y <- df2$time - df2$tstart

df2$n <- 1

df3 <- summaryBy(y + event + n ~ timegroup + treatment + txCode + study + studyCode , FUN=c(sum, max), data=df2)

df3 <- subset(df3, select=-c(event.max, n.max))

names(df3) <- c("spgrp", "treatment", "trtn", "study", "studyn", "y", "nevents", "natrisk", "y.max")

df3$start <- NA

for(i in unique(df3$spgrp)){

df3$start[df3$spgrp==i] <- timepoints2[i]

}

df3$time <- df3$start + df3$y.max

return(df3)

}

| 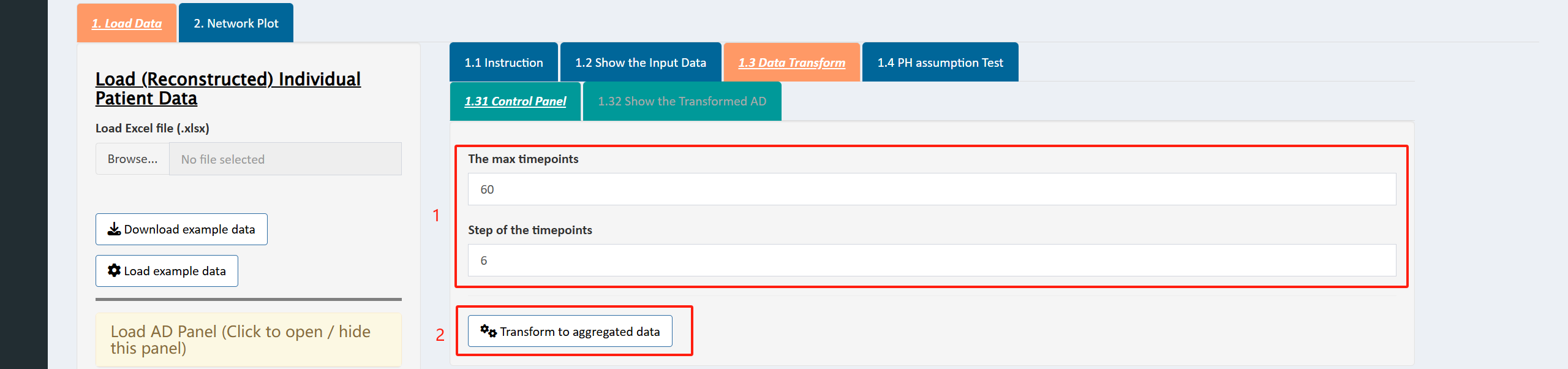 |
| --- |
| 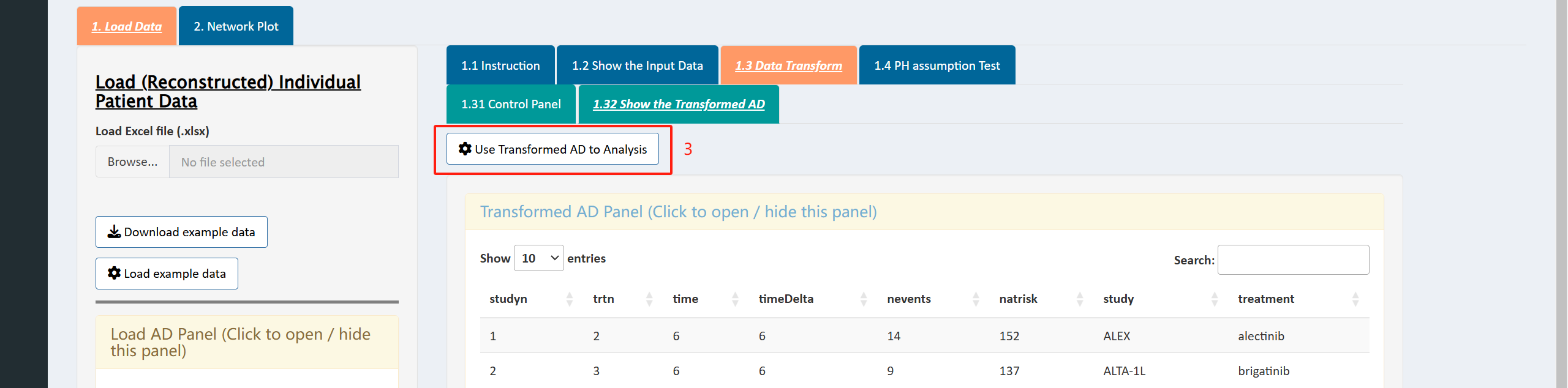 |

Figure 3‑4 Interface of the Data Transform panel

### 3.33 PH assumption tests

In section proportional hazards (PH) assumption tests, users can test whether the PH assumption is held between two arms. This APP provided three methods to test the PH assumption. They are Schoenfeld residual plot^14^, Log-Log plot^3^ and Grambsch-Therneau test^15^. Here is the summarized introduction for these three methods:

**Schoenfeld residual plot**

The Schoenfeld residual test primarily examines the proportional hazards assumption by analyzing the changes in Schoenfeld residuals over time. The basic idea is that if the proportional hazards assumption holds, the Schoenfeld residuals should be randomly distributed over time without showing any systematic trend. In this APP, users can get the P value through the Schoenfeld residual plot. Typically, if P value is smaller than 0.05, PH assumption is considered not held. An example plot (not from NSCLC-ALK network) can be found as Figure 3-5.

| 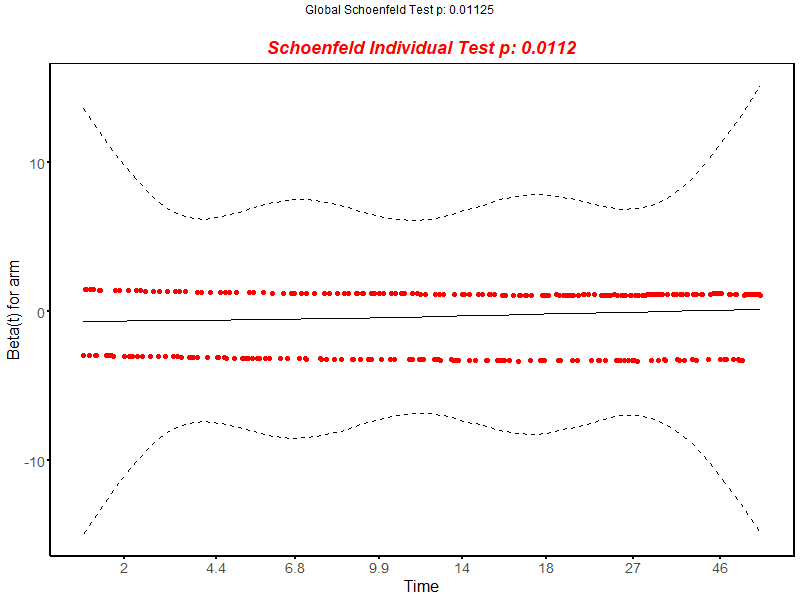 |
| --- |

Figure 3‑5 Example Schoenfeld residual plot

**Grambsch-Therneau test**
The Grambsch-Therneau test is a more formal statistical test based on Schoenfeld residuals. It evaluates the proportional hazards assumption by examining the correlation between Schoenfeld residuals and the ranks of time. In this APP, users can get the P value through the Grambsch-Therneau test. Typically, if P value is smaller than 0.05, PH assumption is considered not held. An example results table (not from NSCLC-ALK network) can be found as Table 3-5. In Table 3-5, **chisq** refers to the chi-square; **df** refers to the degree of freedom; **p** means the p value.

Table 3‑5 Example Grambsch-Therneau test results

|  | chisq | df | p |
| --- | --- | --- | --- |
| arm | 6.43 | 1 | 0.011 |
| GLOBAL | 6.43 | 1 | 0.011 |

**Log-Log plot**

If the PH assumption holds, the log-cumulative hazard curves for different groups should be parallel. Through visual inspection, if two lines are not parallel, PH assumption is considered not held. An example plot (not from NSCLC-ALK network) can be found as Figure 3-6.

| 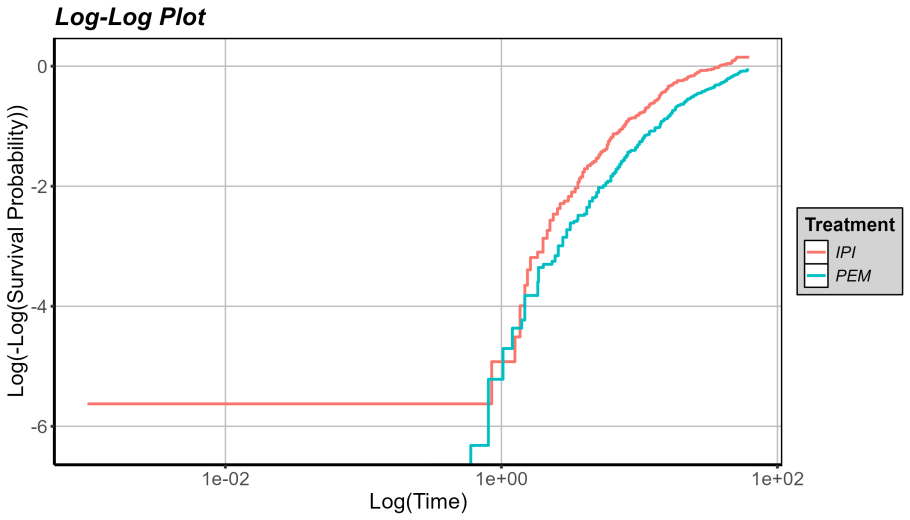 |
| --- |

Figure 3‑6 Example Log-Log plot

Usually, PH assumption uncertain in > 1 trials in the network, users should consider NMA that do not rely on PH assumption; No evidence of a violation of the PH assumption in any of the trials in the network, users can consider PH models.

In NMASurv, according to Figure 3-7 highlight 1, users can select the studies and treatments for PH assumption tests based on their IPD input. Methods for PH assumption tests can also be selected in Figure 3-7 highlight 2. By pressing the button in Figure 3-7 highlight 3, the results of PH assumption tests will be shown. Please note that without inputting IPD, PH assumption tests will not be available to users. Please also note that the examples shown here are not generated based on the studies in the NSCLC-ALK network. We selected another trial (Keynote 006, Ipilimumab vs Pembrolizumab) since the generated results can better illustrate how to tell if the PH assumption is violated.

Here we provide the core codes for PH assumption tests through package “*survival* ” and “*survminer* ”. Please note that the plots in this APP are redrawn through “*ggplot2*”. Through the following codes, users can only get the raw plots.

fit <- coxph(formula = Surv(time, event) ~ arm, data = data)

res1 <- cox.zph(fit)

ggcoxzph(res1) # Schoenfeld residual plot

s1 <- Surv(data$time, data$event)

plot(survfit(s1 ~ data$txCode), col=c("blue", "red"), fun="cloglog", xlab="Log(time)", ylab="log(-log(survival probability))") # Log-Log plot

print(res1) # Results of Grambsch-Therneau tes


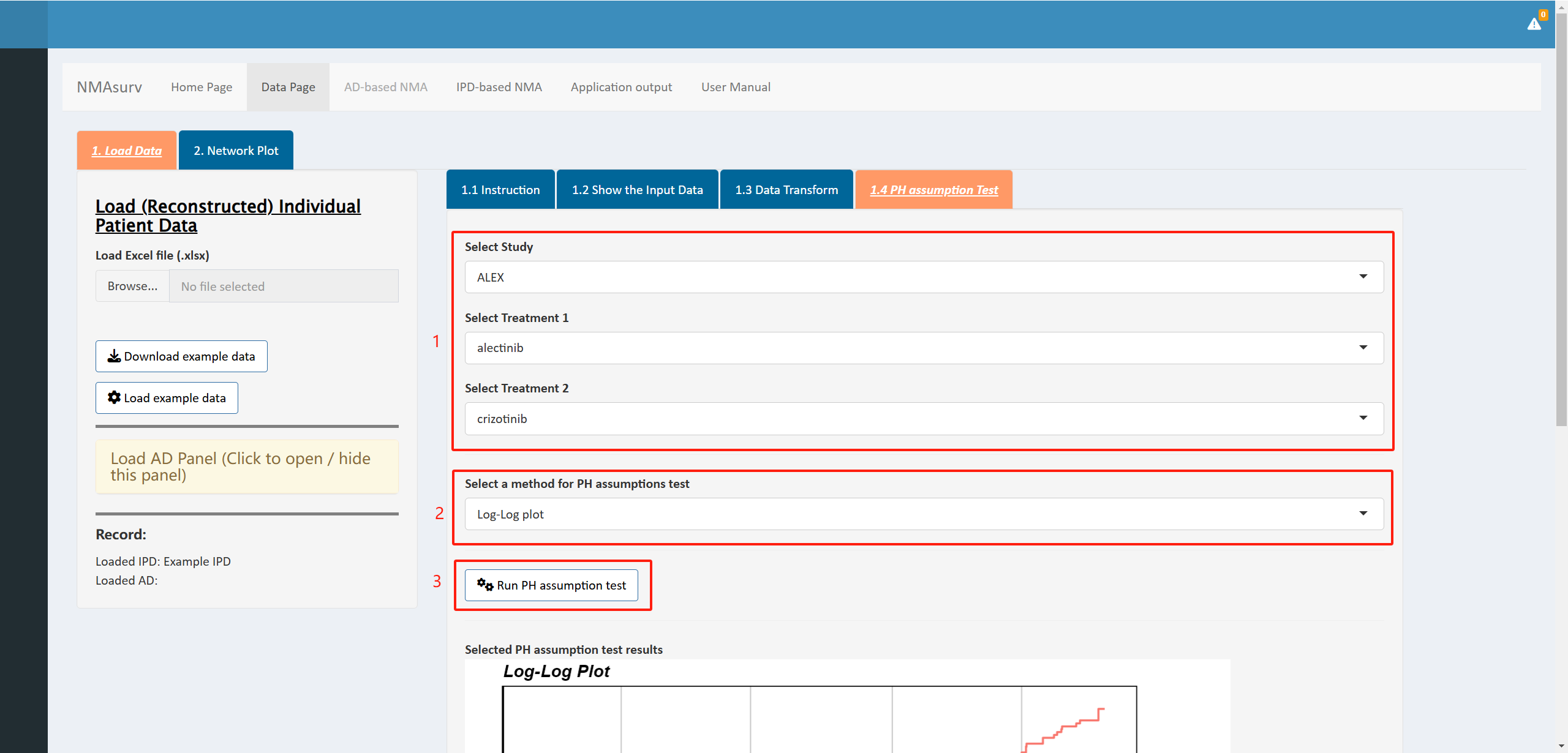


Figure 3‑7 Section PH assumption Test

### 3.34 Network Plot

In section Network Plot, we allow users to draw a network plot based on their own needs. Please note that in this APP, we used the “*netgragh*” function in the “*netmeta*” package to draw a network plot. If users do not like the format of this plot, they can try other functions. For example, a simpler method like “*gemtc*” package or more difficult one like “*igraph*”. An example network plot of “*netgragh*” will be shown as Figure 3-8 (NSCLC-ALK network). Also, users should know that the data used to draw the network plot is different to that used in the Load Data panel. Thus, users should input the data with another data format here.


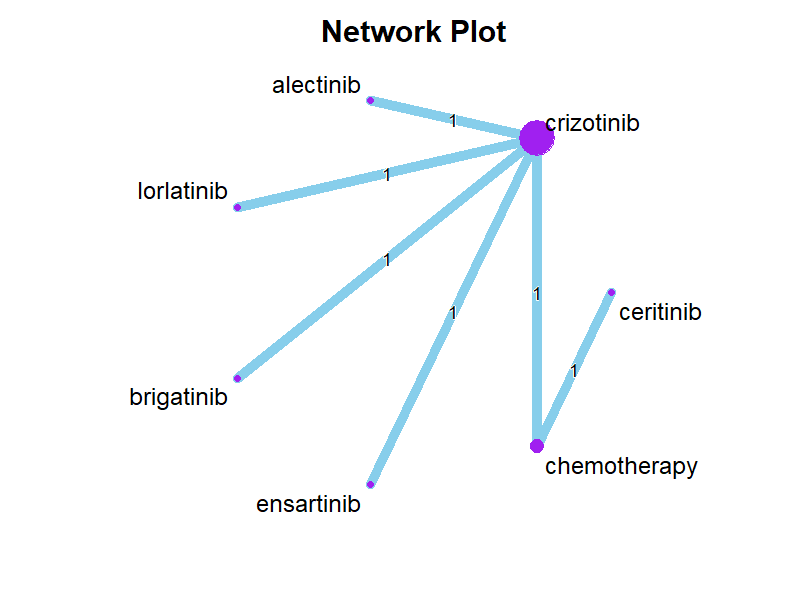


Figure 3‑8 Example network plot (NSCLC-ALK network)

For the data to draw network plot, it should include **nine** columns: studyCode, Study, t1, t2, treat1, treat2, hr, lower, upper. **studyCode** [num] refers to the study code; **Study** [chr] is the study name; **t1,t2** [num] refers to the treatment code; **treat1,treat2** [chr] refers to the treatment name; **hr with lower and upper** [num] refers to the hazard ratio with its upper and lower limits. The data input of NSCLC-ALK network can be found in Table 3-6.

Table 3‑6 Example data input of the network plot (NSCLC-ALK network)

| studyCode | Study | t1 | t2 | treat1 | treat2 | hr | lower | upper |
| --- | --- | --- | --- | --- | --- | --- | --- | --- |
| 1 | ALEX | 1 | 2 | crizotinib | alectinib | 0.67 | 0.46 | 0.98 |
| 2 | CROWN | 1 | 3 | crizotinib | lorlatinib | 0.72 | 0.41 | 1.25 |
| 3 | ALTA-1L | 1 | 4 | crizotinib | brigatinib | 0.81 | 0.53 | 1.22 |
| 4 | eXalt 3 | 1 | 5 | crizotinib | ensartinib | 0.91 | 0.54 | 1.54 |
| 5 | PROFILE 1014 | 6 | 1 | chemotherapy | crizotinib | 0.76 | 0.55 | 1.05 |
| 6 | ASCEND-4 | 6 | 7 | chemotherapy | ceritinib | 0.73 | 0.5 | 1.08 |

Here we also provided another example data here (NSCLC-PDX network) since NSCLC-ALK network does not have a three-arm study. This data here is obtained from Shao et.al. (2022)^7^, which has already been described in detail in section 2.4. This NSCLC-PDX (OS) network included 10 treatments and 6 studies. Since study 6 (Impower150) is a three-arm study, we will split it into 3 lines for input. The example inputted data and generated plot can be found as Table 3-7 and Figure 3-9.

Table 3‑7 Example data input of network plot (NSCLC-PDX network)

| studyCode | Study | t1 | t2 | treat1 | treat2 | hr | lower | upper |
| --- | --- | --- | --- | --- | --- | --- | --- | --- |
| 1 | CameL | 1 | 2 | che | cam+che | 0.73 | 0.53 | 1.02 |
| ...... |  |  |  |  |  |  |  |  |
| 6 | Impower150 | 6 | 5 | bev+che | ate+che | 0.84 | 0.71 | 1 |
| 6 | Impower150 | 6 | 7 | bev+che | ate+bev+che | 0.8 | 0.67 | 0.95 |
| 6 | Impower150 | 5 | 7 | ate+che | ate+bev+che | 0.95 | 0.75 | 1.22 |
| ...... |  |  |  |  |  |  |  |  |
| 9 | Tasuki52 | 6 | 10 | bev+che | niv+bev+che | 0.85 | 0.63 | 1.14 |

| 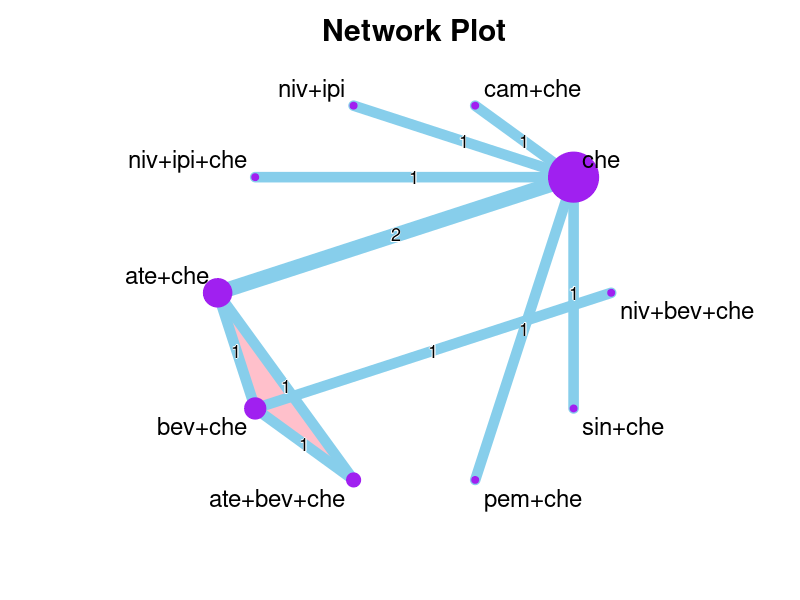 |
| --- |

Figure 3‑9 Example network plot (NSCLC-PDX network)

In NMAsurv, users should input the data first before drawing the network plot (Figure 3-10, highlight 1). The inputted data will be shown in highlight position 3. Then, through the input boxes shown in highlight position 2, users can personalize this plot through modifying “**Title of the plot**”, “**Color of the line**”, “**Multi-arm or not**”, “**Color of the multi-arm area**”, “**Color of the points**”, “**Color of the interior of words**”, and “**Color of the edge of words**”. Through pressing the “draw the plot” button, users can get and download the final plot in highlight position 4. The core codes we used to generate the plot will be shown below:

a <- netmeta(data$lhr, data$se, treat1=data$t1, treat2=data$t2, studlab=data$Study, reference=1)

netgraph(a, labels = lab, offset = 0.02, plastic = F,

col = ...,

multiarm = ...,

col.multiarm = ..., points = T,

col.points = ..., number.of.studies = T, cex=2, cex.number.of.studies = 1.5, col.number.of.studies = ...,

bg.number.of.studies = ..., cex.points = 2*c(count_trt$Freq))

title(..., adj=0.5,cex.main=2.5)

The summarized descriptions of function “*netgraph*” is shown in Table 3-8, a detailed one can be found through clicking the following URL:

(https://www.rdocumentation.org/packages/netmeta/versions/2.8-2/topics/netgraph.netmeta).

Table 3‑8 Summarized descriptions of function "netgraph"

| Name | Description |
| --- | --- |
| a | An object of class netmeta (mandatory). |
| labels | An optional vector with treatment labels. |
| offset | Distance between edges (i.e. treatments) in graph and treatment labels for 2-D plots (value of 0.0175 corresponds to a difference of 1.75% of the range on x- and y-axis). |
| col | A single color (or vector of colors) for lines connecting treatments (edges) if argument plastic = FALSE. Length of the vector must be equal to the number of edges (see list element 'comparisons' in netmeta). |
| plastic | A logical indicating whether the appearance of the comparisons should be in '3D look' (not to be confused with argument dim). |
| multiarm | A logical indicating whether multi-arm studies should be marked in plot. |
| col.multiarm | Either a function from R package colorspace or grDevice to define colors for multi-arm studies or a character vector with colors to highlight multi-arm studies. |
| points | A logical indicating whether points should be printed at nodes (i.e. treatments) of the network graph. |
| cex.points, pch.points, col.points, bg.points | Corresponding size, type, color, and background color for points. Can be a vector with length equal to the number of treatments. |
| number.of.studies | A logical indicating whether number of studies should be added to network graph. |
| cex.number.of.studies | The magnification to be used for number of studies. |
| col.number.of.studies | Color for number of studies. |
| bg.number.of.studies | Color for shadow around number of studies. |
| cex | The magnification to be used for treatment labels. |

|  |
| --- |

| 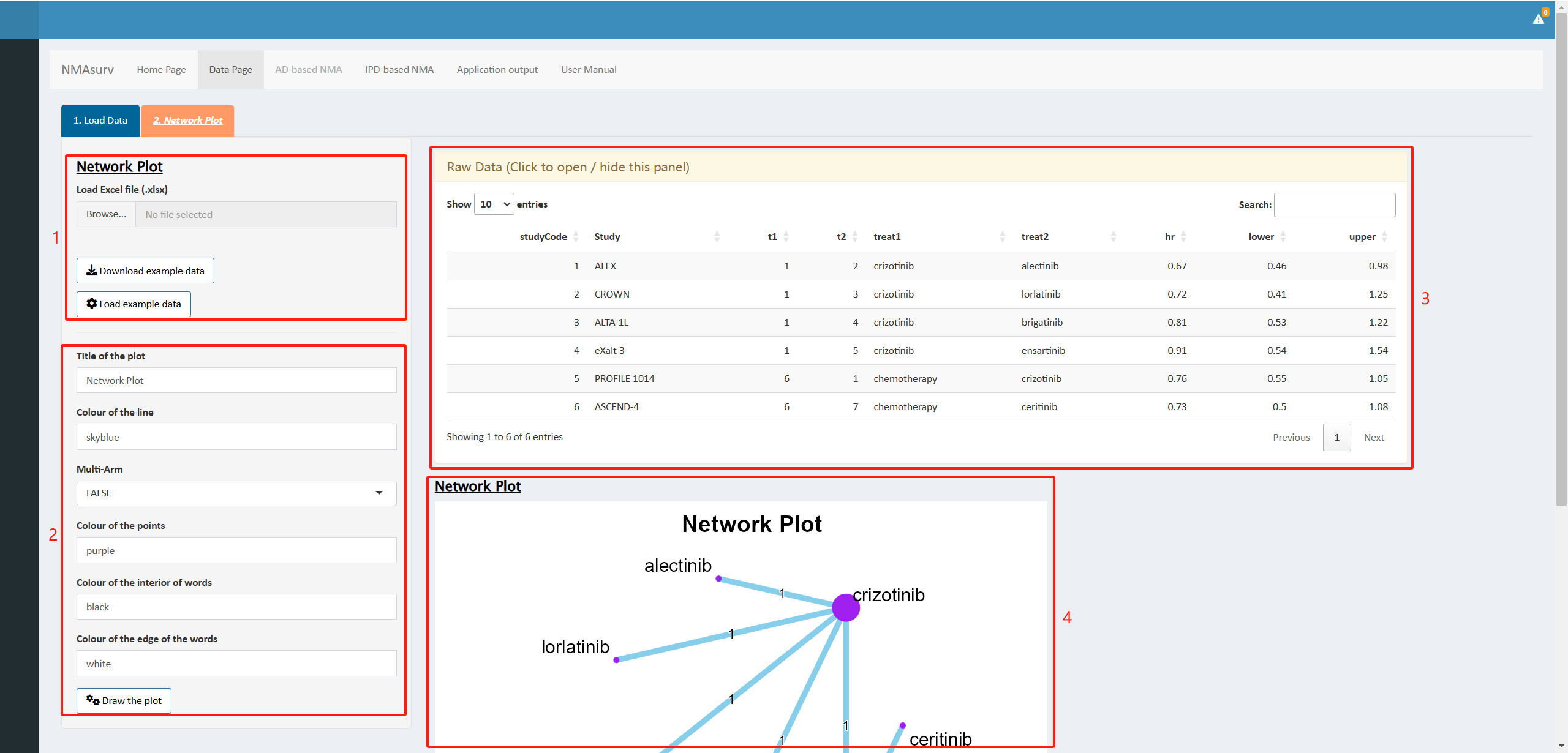 |
| --- |

Figure 3‑10 Section network plot

## 3.4 AD-based NMA

### 3.4.1 Fractional polynomials

The detailed methodology of Fractional polynomials (FP) has been described elsewhere so we do not provide it here^6,16,17^. In this FP NMA section, we use the framework of Wiksten et al.(2020)^5^. We propose a two-step process for fitting FP models. In the first step, an ANOVA-like parameterisation is used to express and fit the models as GLM with time-varying covariates in a frequentist framework. The fit of the models in terms of the AIC is compared (in “FP Step One” panel). The model with the lowest AIC can be selected to fit in the Bayesian setting (or Frequentist setting) in the second step (in “FP Step TWO (FP1)” panel and “FP Step TWO (FP2)” panel). Please note that the core methodology of frequentist analysis in step one and two is the same.

For the fixed-effect model, we provide parameter estimates. Hazard plot and survival plot are also drawn based on these parameters. Use the Difference in **Beta** and the **Beta** of reference treatment to calculate other **Beta** values. Then, hazard over time for each of the interventions can be calculated through the function with **Beta**. In addition, through **d** (**trtf** in Frequentist setting), Hazard Ratio between selected treatments can be calculated. This process can also be realized easily through EXCEL. For Example: For reference treatment a and intervention b, we construct a FP1 model with power = -2. We get *d_0ab_, d_1ab_, Beta_0a_, Beta_1a_, Beta_0b_, Beta_1b_*.
Note:

*Beta_0b_=Beta_0a_+d_0ab_; Beta_1b_=Beta_1a_+d_1ab_*

Thus,

*Log(HR_ab_(t))=d_0ab_+d_1ab_*t^-2^,*

*Log(Hazard_a_(t))=Beta_0a_+Beta_1a_*t^-2^,*

*Log(Hazard_b_(t))=Beta_0b_+Beta_1b_*t^-2^*

$$\left( \begin{matrix} \beta_{0k} \\ \beta_{1k} \end{matrix} \right)=\left\{ \begin{matrix} \left( \begin{matrix} \mu_{0} \\ \mu_{1} \end{matrix} \right) if k=a \\ \left( \begin{matrix} \mu_{0} \\ \mu_{1} \end{matrix} \right)+\left( \begin{matrix} d_{0} \\ d_{1} \end{matrix} \right) if k=b \end{matrix} \right.$$

Note:*Beta_0a_, Beta_0b_* are usually calculated as average from study specific estimates(*μ_0_, μ_1_, μ_2_*) of the reference treatment^5,6^. One alternative is to fit and extrapolate the selected reference treatment with FP model to calculate the Beta. However, biases may be introduced through this method.

For random-effects model, we provide the parameter estimates only. Plots can be drawn through the same methods. In addition, we only consider the model with a heterogeneity parameter for *d_0_* in random-effects model. We do not consider all heterogeneity parameters (*d_0_, d_1_, d_2_* in FP2) since we believe that the methodology still needs further development^6^. (Bayesian analysis using the random-effects model would require careful specification of the prior for between-study heterogeneity. Also, maximum likelihood estimation can be problematic at this time, in which the number of parameters increases with the number of studies.) An example of Bugs codes for random-effects FP models with all heterogeneity parameters can be found in Jansen JP (2011)^6^. In Jansen's study, two kinds of random-effects models showed similar fit results.

| 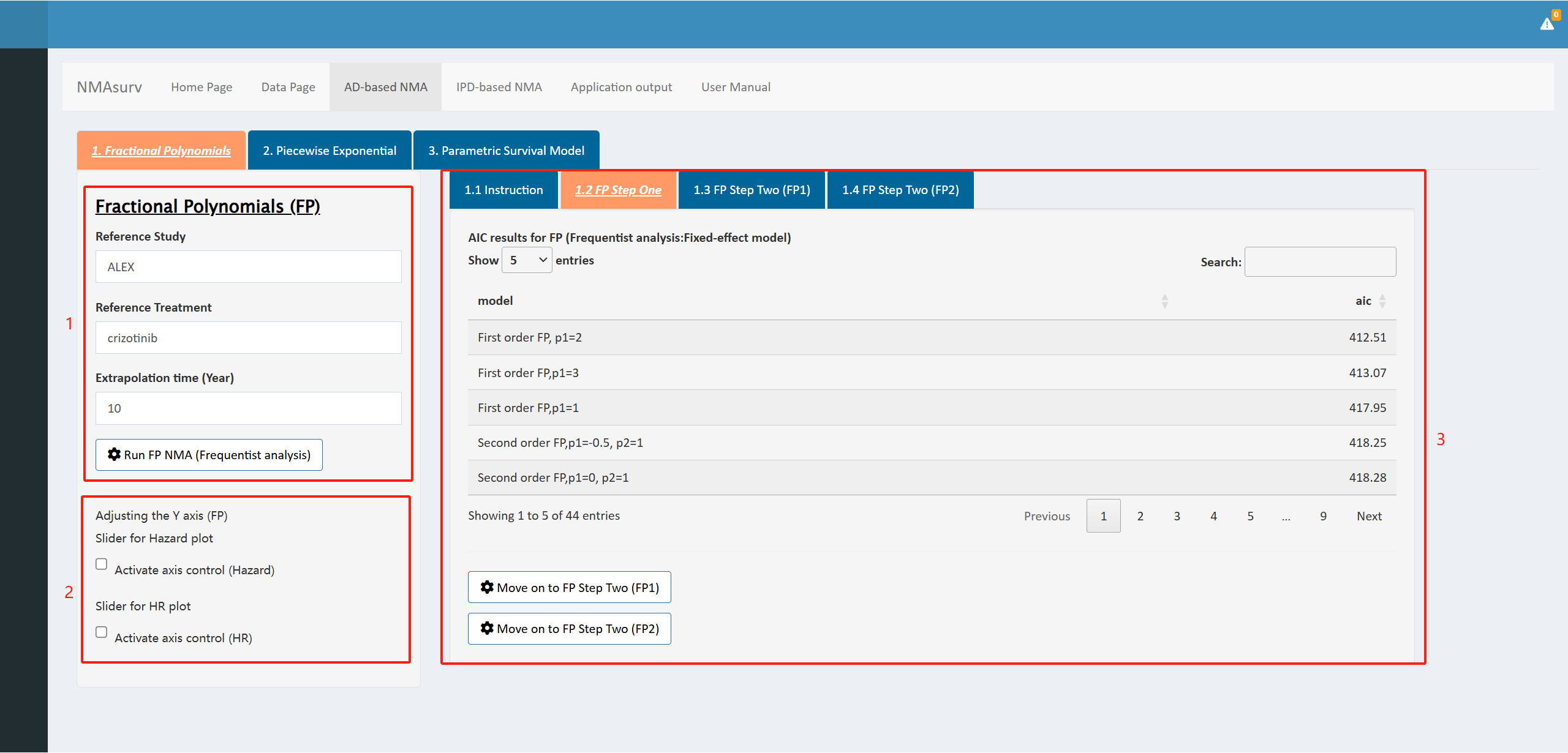 |
| --- |

Figure 3‑11 Section FP 1 (AD-based NMA)

According to Figure 3-11, through the input box in highlight position 1, users can input their **Reference Study**, **Reference Treatment** and **Extrapolation Time**. Then, after pressing the “Run FP NMA (Frequentist analysis)”, users can get the results of step one (“frequentist framework”), which can be found in “FP Step One” panel (Figure 3-11, highlight 3). After selecting the best FP1 and FP2 models, users can move to FP1 and FP2 for step two analysis easily through the buttons provided below the step one results. In the sidebar panel, users can also adjust the Y axis for their drawn plots (will be used later) in highlight position 2.

Take FP1 (Power=1, Bayesian analysis, the fixed-effect model) as an example, according to Figure 3-12 (a), users can input the power of FP1 in highlight position 1. After running the fixed-effect FP1 model, users can get the results which are shown in Figure 3-12 (b) and Figure 3-13 (NSCLC-ALK network). According to Figure 3-12 (b), users can check which model they are running in highlight 2; know the GOF estimates in highlight 3; and download the coefficient table in highlight 4. Hazard plots, survival plots, HR plots are shown in the panel in Figure 3-13. Please note that users can download the extrapolated HR data through the button in Figure 3-13, highlight 1. Y-axis for hazard plots and HR plots can be modified through the panel on the sidebar. NMAsurv provided the results output function, user can press the button shown in Figure 3-12, highlight 1 to export the results to the “Application Output” panel. Detail information about the output function can be found later. For Frequentist analysis, most procedures are similar to Bayesian analysis.

In NMAsurv, users can adjust the Jags parameters for running the Bayesian analysis in FP1 in Figure 3-12, highlight 2. In this tool, “R2jags” was used. A detailed description of this package is available on (https://cran.r-project.org/web/packages/R2jags/R2jags.pdf). A brief description can be found in Table 3-9. The same approach will be used in subsequent models.

Table 3‑9 Brief introduction of R2jags

| Name | Description |
| --- | --- |
| data | (1) a vector or list of the names of the data objects used by the model, (2) a (named) list of the data objects themselves, or (3) the name of a "dump" format file containing the data objects, which must end in ".txt", see example below for details. |
| inits | a list with n.chains elements; each element of the list is itself a list of starting values for the BUGS model, or a function creating (possibly random) initial values. If inits is NULL, JAGS will generate initial values for parameters. |
| parameters.to.save | character vector of the names of the parameters to save which should be monitored. |
| model.file | file containing the model written in BUGS code. Alternatively, as in R2WinBUGS, model.file can be an R function that contains a BUGS model that is written to a temporary model file (see tempfile) using write.model |
| n.chains | number of Markov chains (default: 3) |
| n.iter | number of total iterations per chain (including burn in; default: 2000) |
| n.burnin | length of burn in, i.e. number of iterations to discard at the beginning. Default is n.iter/2, that is, discarding the first half of the simulations. If n.burnin is 0, jags() will run 100 iterations for adaption. |
| n.thin | thinning rate. Must be a positive integer. Set n.thin > 1 to save memory and computation time if n.iter is large. Default is max(1, floor(n.chains * (n.iter-n.burnin) / 1000)) which will only thin if there are at least 2000 simulations. |

| \| (a)  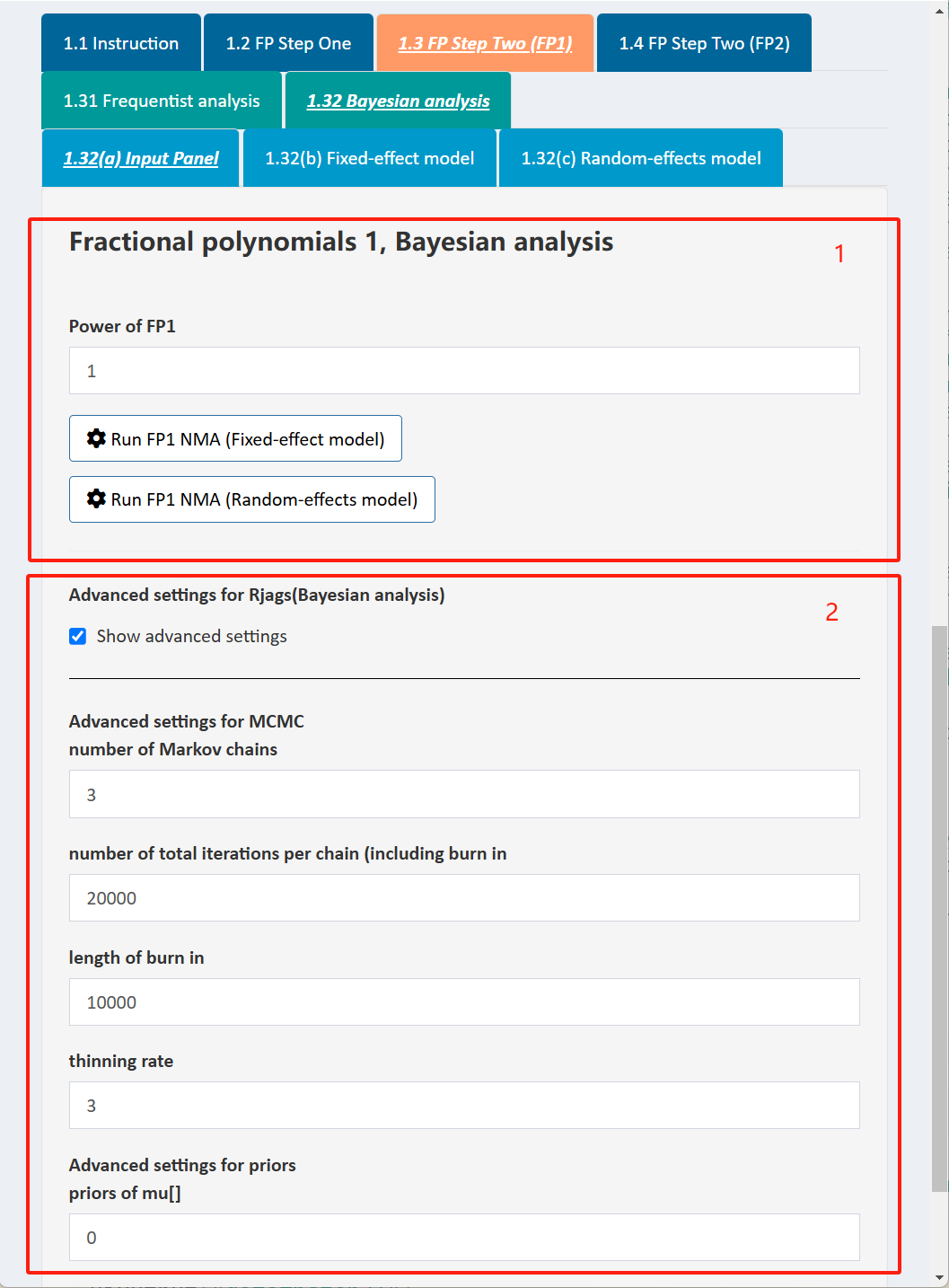 \| (b)  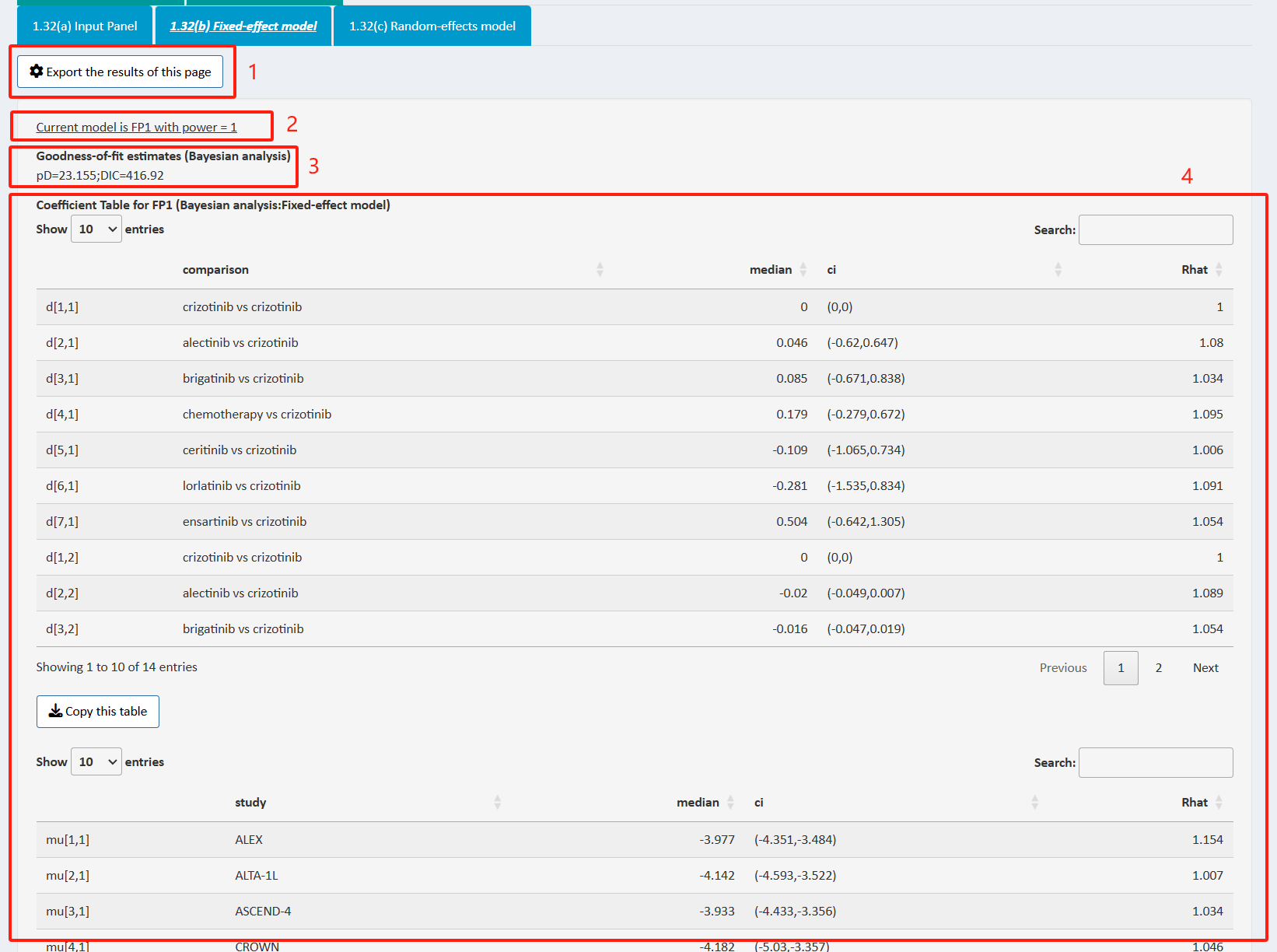 \| \| --- \| --- \| |
| --- | --- | --- |

Figure 3‑12 Section FP1 (Power=1, Bayesian analysis, the fixed-effect model)

| \| 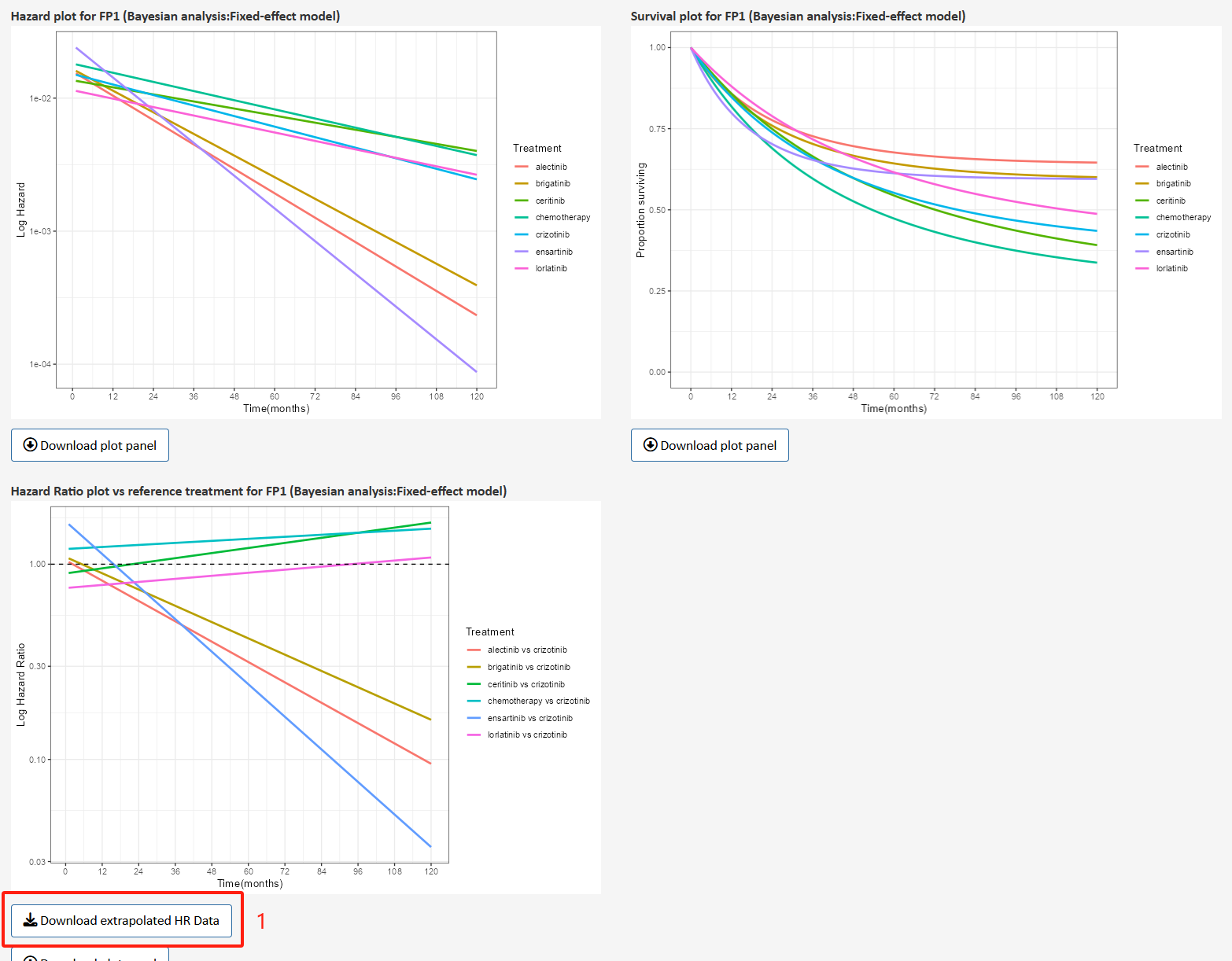 \| \| --- \| |
| --- | --- |

Figure 3‑13 Section FP1 (Power=1, Bayesian analysis, the fixed-effect model) (Continued)

For Frequentist analysis results, this APP will provide estimated value, standard error, and upper and lower limits of treatment effects. For Bayesian analysis results, this APP will provide median and confidence interval for parameters **mu** (the parameters Beta of the “baseline” treatment) and **d** (the difference in Beta_0_ and Beta_1_ of the log hazard for treatment b relative to a). To understand these, users can refer to the formula mentioned earlier. This APP will also report the indicator of “**Rhat**”, which is used as convergence diagnostics for Markov Chains. “**Rhat**” compares the between- and within-chain estimates for model parameters and other univariate quantities of interest^18^. If chains are not mixed well (ie, the between- and within-chain estimates don't agree), R-hat is larger than 1. It is recommended to run at least four chains by default and only use the sample if R-hat is less than 1.05.

Here, we provide the codes for users to run the fixed-effect FP NMA model^5^. Notably, we take the example of FP 2 with power -2 and 2. And only core codes are provided here:

**## Codes for preparation**

# set reference study and treatment

ref.study <- ...

ref.trt <- ...

# import the data

data <- c(...)

studies <- c(...)

treatments <- c(...)

**## Codes for frequentist analysis**

# Model formula

models <- list("Second order FP,p1=-2, p2=2" = list(g1=function(x){x^-2}, g2=function(x){x^2}, f1=function(x){x^-2}, f2=function(x){x^2}))

# Fit the model

fit.KM.NMA<-function(bf){

data.new=data

data.new$g0=1

data.new$f0=1

data.new$g1=bf[[1]](data.new$time)

data.new$g2=bf[[2]](data.new$time)

data.new$f1=bf[[3]](data.new$time)

data.new$f2=bf[[4]](data.new$time)

f=cbind(nevents,natrisk-nevents)~trtf*f0+studyf*g0+trtf*f1+trtf*f2+studyf*g1+studyf*g2

glm(f,family=binomial(link=cloglog),data=data.new,offset = log(timeDelta))

}

fits=lapply(models,fit.KM.NMA)

**## Codes for preparing data for JAGS**

d_arms <- data %>%

group_by(studyn, trtn) %>%

slice(1) %>%

group_by(studyn) %>%

dplyr::mutate(arm = 1:n(), n_arms = max(arm)) %>%

select(studyf, trtf, studyn, trtn, arm, n_arms)

d_arms

d_std <- d_arms %>%

group_by(studyn) %>%

select(studyn, n_arms) %>%

slice(1)

d_std

dat <- km %>%

left_join(d_arms, by = c("studyf", "trtf", "studyn", "trtn"))

d_trts <- dat %>%

mutate(studyn.arm = interaction(studyn, arm)) %>%

filter(!duplicated(studyn.arm)) %>%

select(studyn, arm, trtn) %>%

arrange(studyn, arm) %>%

tidyr::spread(key = arm, trtn, drop = FALSE)

d_trts

Nobs <- nrow(dat)

dat_jg <- list(Nobs = Nobs, Ns = nrow(d_std), Na = d_std$n_arms, r = dat$nevents, n = dat$natrisk, time = dat$time, dt = dat$timeDelta, s = dat$studyn, a = dat$arm, t = as.matrix(select(ungroup(d_trts), -studyn)), Ntrt = max(select(ungroup(d_trts), -studyn), na.rm = TRUE))

**## Codes for Bayesian analysis**

model.pars <- list(P1 = -2, P2=1)

set.seed(...)

fit <- jags(model.file = "...txt",

data = c(dat_jg,

list(prior.mean = rep(0, 3)),

list(prior.prec = diag(rep(0.0001, 3))),

model.pars),

parameters = c("d", "mu", "Beta"),

n.chains = 3, n.iter = 200000, n.burnin = 50000, n.thin = 3)

Please note that codes for the random-effects model are similar to the fixed-effect model. Thus, we will not provide them here. JAGS codes for running the fixed-effect and the random-effects FP2 Bayesian analysis will be provided in the Appendix.

### 3.4.2 Piecewise Exponential Model

The framework of Piecewise Exponential Model (PWE) model is similar to that of FP. Users can use two steps to build a PWE model. A PWE function can also be written in the form of ANOVA-like parameterisation, so that it can be fitted in the GLM framework^5,19^. Currently, only the fixed-effect models are available for PWE NMA. The choice of where to place cut points and how many cut points could result in many models being fitted before the best model can be selected. We recommend users to use Frequentist analysis to select suitable models before moving to Bayesian analysis.

Using the difference in **Beta** and the **Beta** of reference treatment, users can calculate other **Beta** values. Then, hazard over time for each of the interventions can be calculated through the function with **Beta**. In addition, through **d** (**trtf** in Frequentist setting), Hazard Ratio between selected treatments can be calculated. This process can also be realized easily through EXCEL. For Example: For reference treatment a and intervention b, we construct a PWE model with time point = 2. We get *d_0ab_, d_1ab_, Beta_0a_, Beta_1a_, Beta_0b_, Beta_1b_*.

Note:

*Beta_0b_=Beta_0a_+d_0ab_; Beta_1b_=Beta_1a_+d_1ab_- Beta_0b_*

Thus,

*Log(HR_ab_(t))=d_0ab_(0<=t<2); d_1ab_(t>=2),*

*Log(Hazard_a_(t))=Beta_0a_(0<=t<2); Beta_1a_(t>=2),*

*Log(Hazard_b_(t))=Beta_0b_(0<=t<2); Beta_1b_(t>=2).*

Note:*Beta_0a_*, *Beta_0b_* are usually calculated as average from study specific estimates (*μ_0_*, *μ_1_*, *μ_2_*) of the reference treatment.

According to Figure 3-14, through the input box in highlight position 1, users can input their **Reference Study**, **Reference Treatment** and **Extrapolation Time** which is similar to the FP models. Then, users can input the cutpoint (unit: month) of PWE in highlight position 2. After pressing the buttons below, users can get the hazard plots, survival plots, HR plots and coefficient tables which are similar to the FP models. Similar to FP NMA, users can modify the settings for JAGS to run the Bayesian analysis. Take PWE 1, cutpoint = 12 as an example, the results are shown in Figure 3-15 (NSCLC-ALK network). Procedures for running PWE 2 is similar to those for PWE 1.

Here, we provide the codes for users to run the fixed-effect PWE NMA model^5^. Notably, we take the example of PWE model with two cutpoints 2 and 12. The codes for data preparation and running Frequentist analysis and Bayesian analysis in PWE is quite similar to that in FP, thus we will not elaborate on it again. Users only need to make simple modifications. We only provide the codes for model formula in PWE here. JAGS codes for PWE with two cutpoints can be found in Appendix.

models <- list("PWE2" = list(g1=function(x){as.numeric(x > 2 & x <= 12)}, g2=function(x){as.numeric(x > 12)}, f1=function(x){as.numeric(x > 2 & x <= 12)}, f2=function(x){as.numeric(x > 12)}))

| 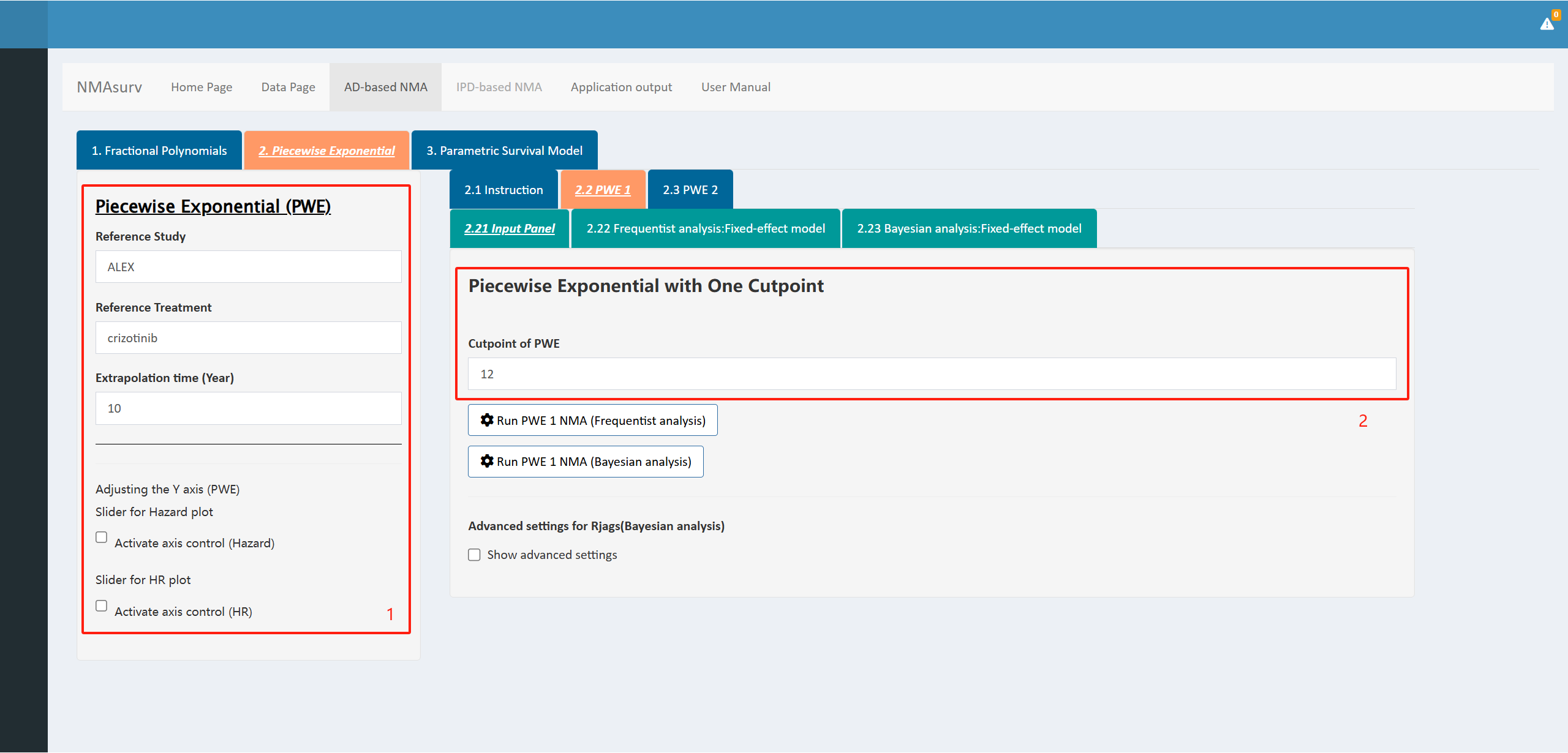 |
| --- |

Figure 3‑14 Section PWE 1 input panel

| 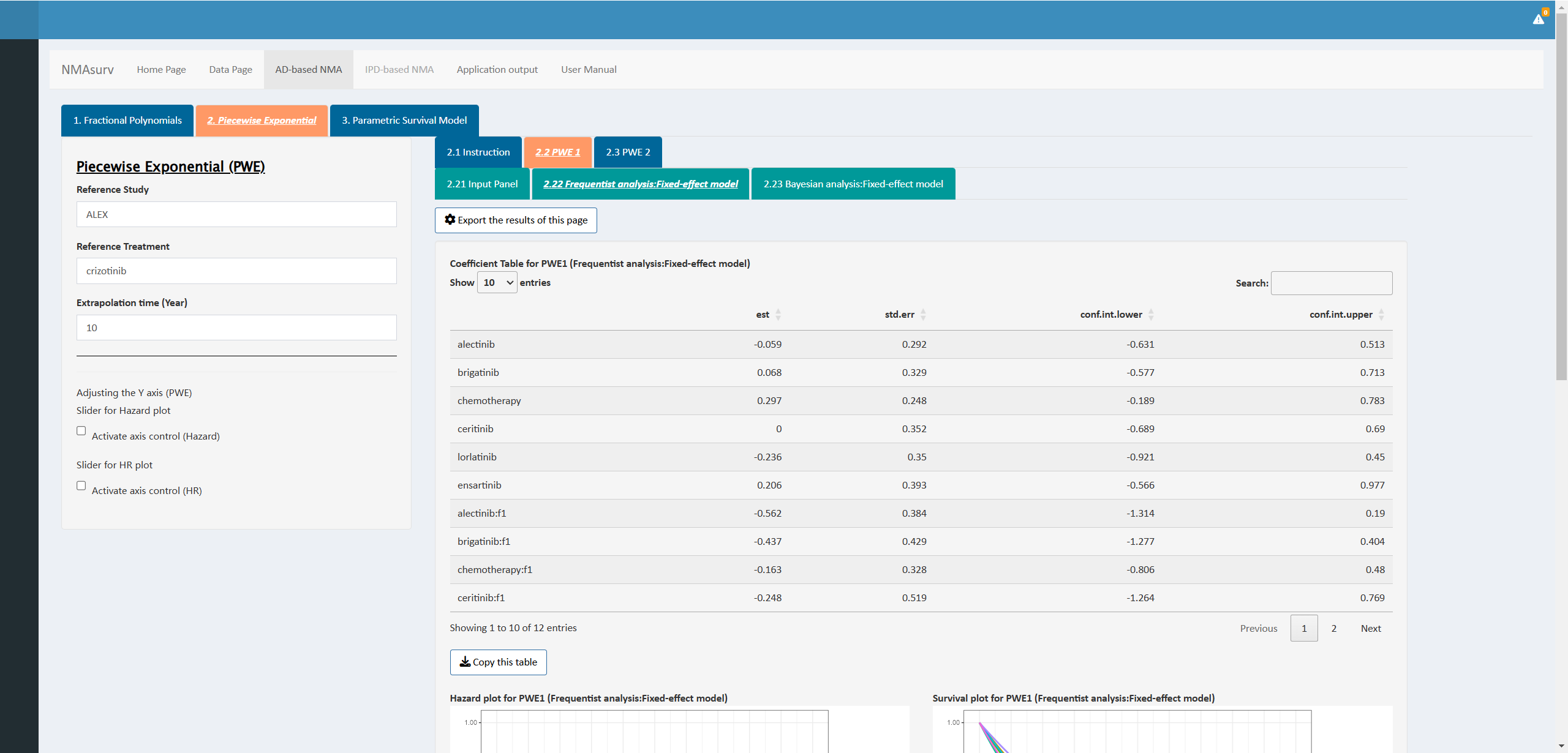 |
| --- |

Figure 3‑15 Example results of PWE 1 (Cutpoint = 12, Frequentist analysis, fixed-effect model)

### 3.4.3 Parametric survival model

In the part of Parametric survival model (PSM), we only include Bayesian analysis (Fixed-effect model and random-effects model). Models including Weibull, Gompertz, log-logistic, log-normal are considered^11^. Use the Difference in Beta and the Beta of reference treatment to calculate other Beta values. Then, hazard over time for each of the interventions can be calculated through the function with Beta. In addition, through d, Hazard Ratio between selected treatments can be calculated. This process can also be realized easily through EXCEL. For Example: For reference treatment a and intervention b, we construct a PSM Weibull model. We get *d_0ab_, d_1ab_, Beta_0a_, Beta_1a_, Beta_0b_, Beta_1b_*.
Note:

*Beta_0b_=Beta_0a_+d_0ab_; Beta_1b_=Beta_1a_+d_1ab_*

Thus,

*Log(HR_ab_(t))=d_0ab_+d_1ab_*log(t),*

*Log(Hazard_a_(t))=Beta_0a_+Beta_1a_*log(t),*

*Log(Hazard_b_(t))=Beta_0b_+Beta_1b_*log(t).*

Note: Beta_0a_, Beta_0b_ are usually calculated as average from study specific estimates(*μ_0_*, *μ_1_*, *μ_2_*) of the reference treatment. We provide the formulas for four distributions here:

**Weibull:**

$$f(t)=a+b*\log(t)$$

**Gompertz:**

$$f(t)=a+b*t$$

**Log-Logistic:**

$$f(t)=\log(\frac{\frac{e^{b}}{e^{a}}*(\frac{t}{e^{a}})^{e^{b}-1}}{1+(\frac{t}{e^{a}})^{e^{b}}})$$

**Log-Normal:**

$$f(t)=\frac{(2\pi)^{-0.5}*e^{-\frac{(\frac{\log(t)-a}{e^{b}})^{2}}{2}}}{e^{b}*t*pnorm(-\frac{\log(t)-a}{e^{b}})}$$

Please note that FP1 with power=0 is equal to Weibull, while FP1 with power=1 is equal to Gompertz. For formula forms which are easier to understand, please refer to the description in the APP. For more information about these formulas, please refer to other studies^20-22^. Based on the control panel shown in Figure 3-16 (a) and the results panel shown in Figure 3-16 (b), users can run the NMA models and check the generated results (NSCLC-ALK network). Since procedures for all four distributions are quite similar to FP and PWE NMA, we will not describe them here. Like FP and PWE NMA, users can modify the settings for JAGS to run the Bayesian analysis.

Codes for PSM are quite similar to those for FP and PWE, thus we do not provide them here. JAGS codes for the fixed-effect PSM of four distributions can be found in Appendix.

| \| (a)  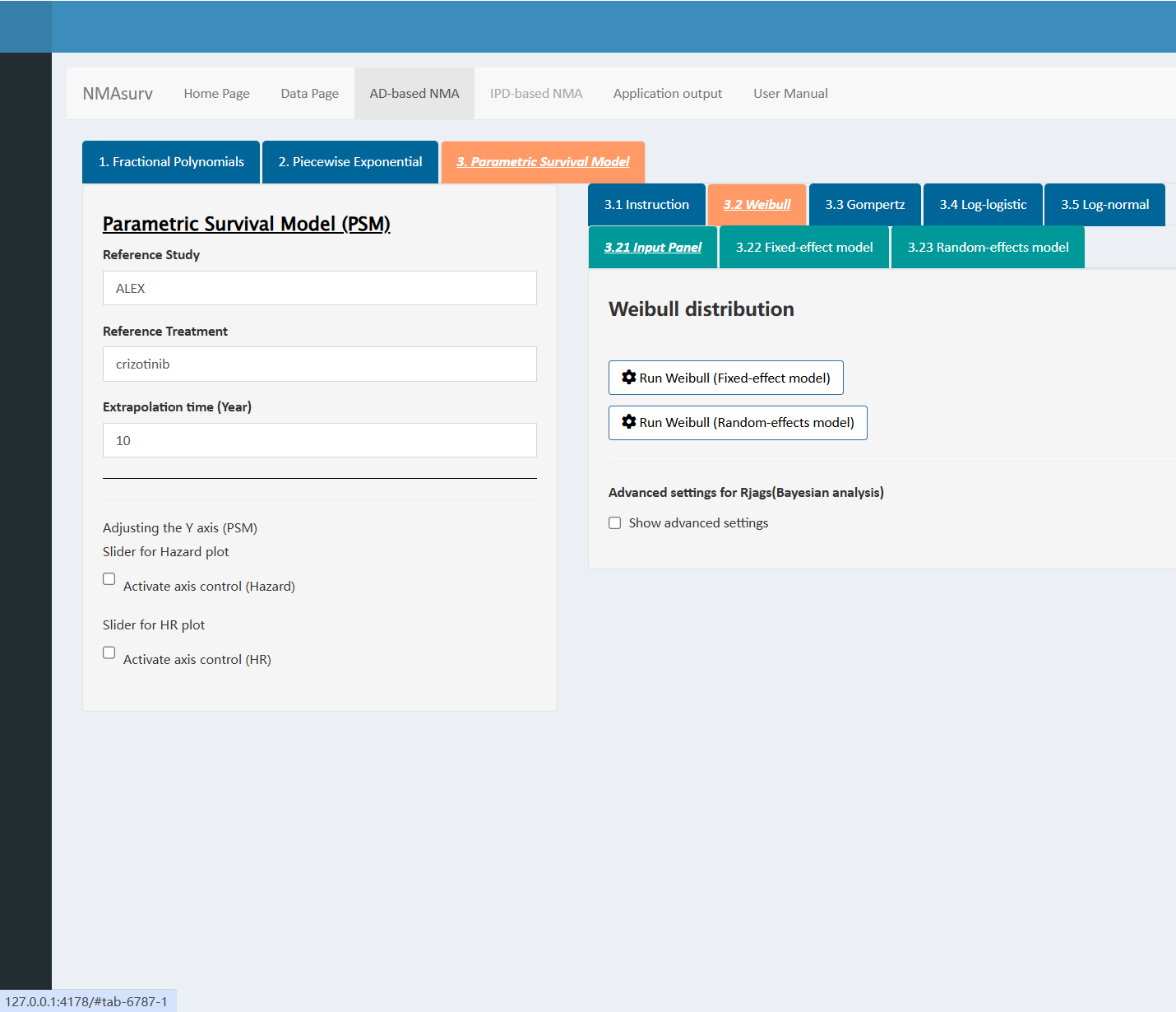 \| (b)  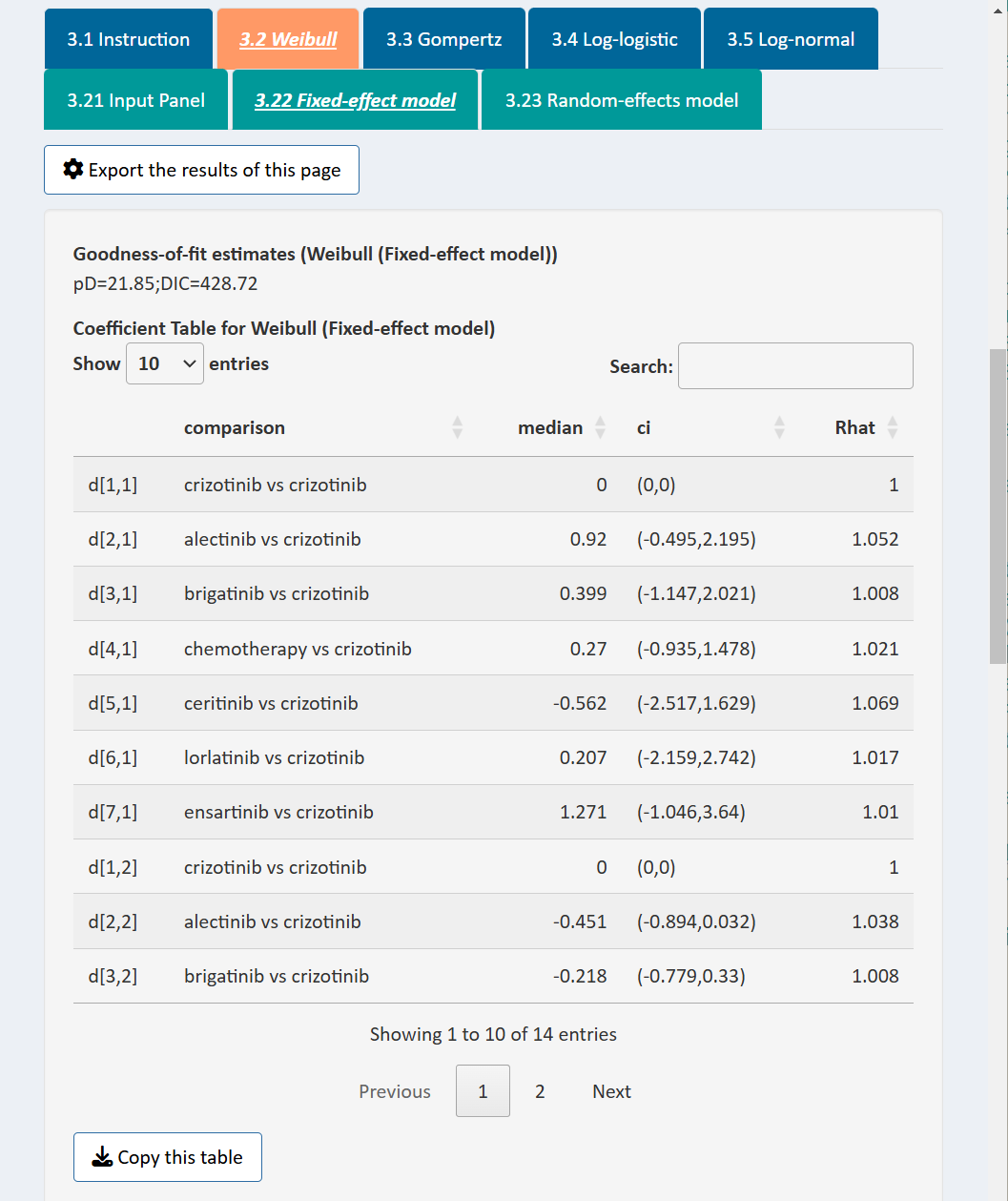 \| \| --- \| --- \| |
| --- | --- | --- |

Figure 3‑16 Section PSM (AD-based NMA)

## 3.5 IPD-based NMA

### 3.5.1 COX PH model

In the part of COX PH model, we only include Bayesian analysis (Fixed-effect model). Treatment 1 (Code 1) is considered as the reference treatment. Changing the reference treatment can be realized through modifying the data.

The Cox PH model was fitted using a two-stage approach according to the method of Freeman SC et.al. (2022)^3^. In the first stage, a Cox PH model was fitted individually to each trial to obtain an estimate of the log HR for the treatment effect and its corresponding standard error^23^.

$$h_{j,ab}(t)=h_{0j,ab}(t)\exp(\alpha_{j,ab}x_{ij})$$

In this formula, *h_j,ab_(t)* is the hazard function for treatment *b* compared to the baseline treatment *a* in trial; *j*, *h_0j,ab_(t)* is the baseline hazard function for trial *j*; *x_i_*_j_ is the treatment indicator variable for patient *i* from trial *j*, taking the value 0 if patient *i* receives the baseline treatment *a* and the value 1 if patient *i* receives treatment *b*; and *α_j,ab_* refers to the HR for a patient receiving treatment b compared to the baseline treatment *a* in trial *j*. In the second stage, the treatment effect estimate was synthesised through a standard fixed-effect NMA model.

This APP will provide the results of HR (compared with the reference treatment). When HR is obtained, the user can calculate the cumulative hazard (*H(t)*) for reference treatment (written as **cumhaz_ref**). Then, multiplying HR with the **cumhaz_ref**, the cumulative hazard for each treatment (written as **cumhaz**) can be obtained. Finally, through formula '**exp(-cumhaz)**', the survival rate (*S(t)*) can be calculated.

$$H{{R_{\text{ab}}}_{,}}_{t_{0}\to t_{1}}=\frac{\Delta H(t{{)_{b}}_{,}}_{t_{0}\to t_{1}}}{\Delta H(t{{)_{a}}_{,}}_{t_{0}\to t_{1}}}$$

$$H(t)=-\ln S(t)$$

Please note that in the first formula, *t_0_* to *t_1_* approaches zero infinitely. Typically, HR value is enough for common NMA. We provide the calculation of survival rate here in case some users need to conduct further research like cost-effectiveness analysis.

The operation of COX PH model is quite simple. According to Figure 3-17, users only need to press the button in highlight position 1 to get the results of treatment effects shown in highlight position 2 (NSCLC-ALK network). Users can also modify the settings for JAGS to run the Bayesian analysis.

To realize the COX PH model, users have to get the hazard ratio for each trial based on the inputted IPD. Detailed information of the methodology to obtain the data can be found elsewhere^3^. Thus, we do not give any description here. In summary, this stage can be implemented using the “*coxph”* function from the “*survival”* package. In NMAsurv, this process of getting data will be completed by the codes so that users do not need to calculate the HRs themselves. We provide this reminder in case some users want to program themselves. After getting the data, NMAsurv will directly use JAGS to run the fixed-effect model to get the estimated HRs. The codes for running JAGS are similar to the AD-based NMA, and we will not discuss them here. JAGS code to run the COX PH NMA can be found in the Appendix. An example of data input of COX PH NMA can be found in Table 3-10 (melanoma network). We selected to put the data of melanoma network here since it contains a three-arm study, which can better help to display the data structure. JAGS codes for the fixed-effect COX PH model can be found in the Appendix.

Table 3‑10 Example of data input of COX PH NMA

| Study | Treatment1 | Treatment2 | HR | SE | Log HR | Variance^a^ |
| --- | --- | --- | --- | --- | --- | --- |
| BREAK-3 | 1 | 4 | 0.824 | 0.176 | -0.193 | NA |
| BRIM-3 | 1 | 6 | 0.799 | 0.087 | -0.225 | NA |
| CheckMate 066 | 1 | 9 | 0.463 | 0.125 | -0.771 | NA |
| CheckMate 069 | 8 | 10 | 0.754 | 0.271 | -0.282 | NA |
| COBRIM | 6 | 7 | 0.687 | 0.127 | -0.376 | NA |
| COMBI-d | 4 | 5 | 0.759 | 0.125 | -0.276 | NA |
| COMBI-v | 5 | 6 | 1.404 | 0.135 | 0.339 | NA |
| Hodi 2014 | 8 | 12 | 0.669 | 0.199 | -0.402 | NA |
| Keynote 006 | 8 | 11 | 0.730 | 0.095 | -0.314 | NA |
| Ribas 2013 | 1 | 2 | 0.878 | 0.088 | -0.130 | NA |
| Robert 2011 | 1 | 3 | 0.730 | 0.098 | -0.315 | NA |
| Robert 2013 | 1 | 13 | 0.828 | 0.247 | -0.188 | NA |
| CheckMate 067 | 8 | 9 | 0.665 | 0.102 | -0.408 | 7.08E-09 |
| CheckMate 067 | 8 | 10 | 0.545 | 0.107 | -0.607 | 7.08E-09 |
| a: variance of baseline treatment  Note: this example data is generated based on the melanoma network. | | | | | | |

| 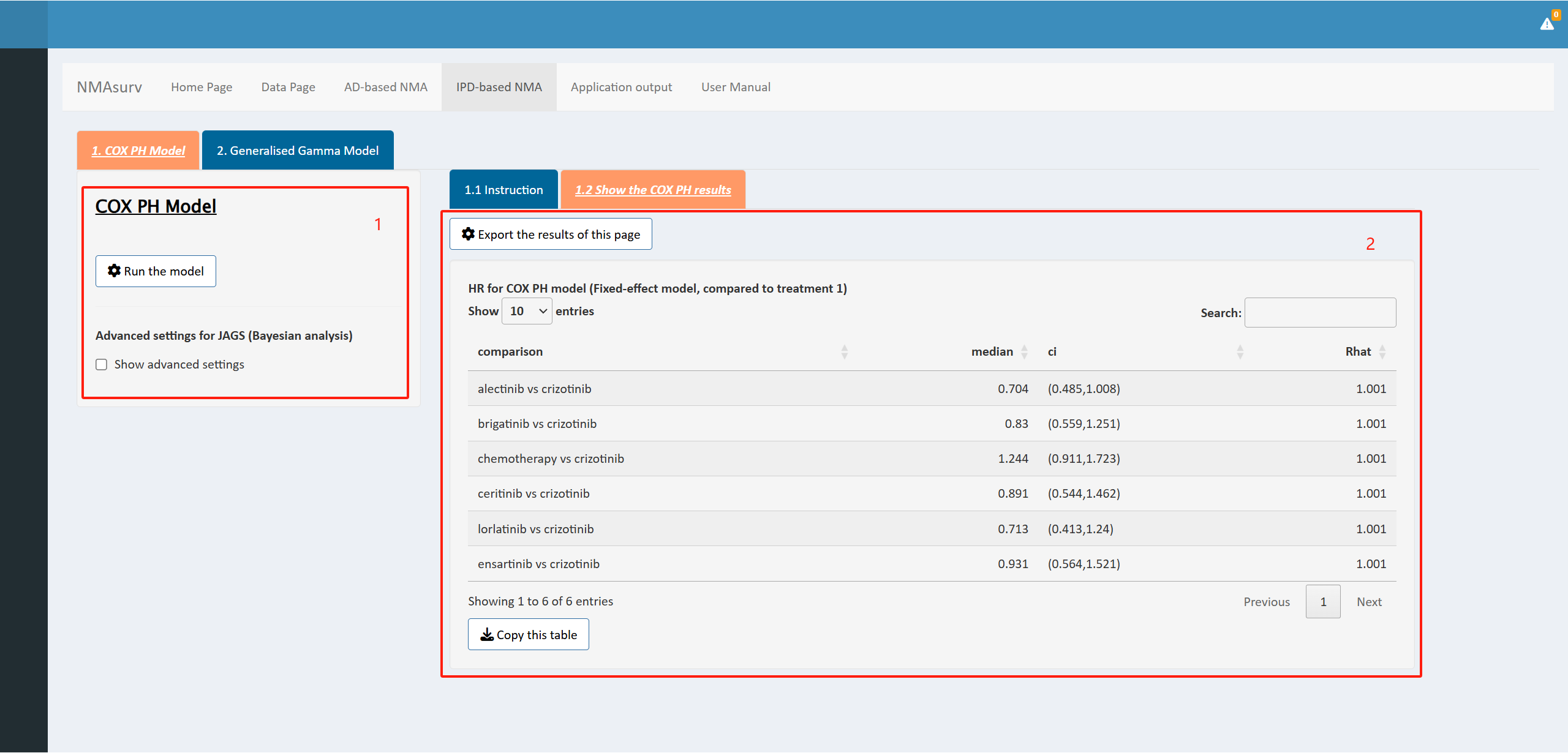 |
| --- |

Figure 3‑17 Section COX PH model

### 3.5.2 Generalized Gamma Model

Similar to COX PH Model, in the part of Generalized Gamma Model, we only include Bayesian analysis (Fixed-effect model). Treatment 1 (Code 1) is considered as the reference treatment. Changing the reference treatment can be realized through modifying the data. The Generalized Gamma Model was fitted using a two-stage process according to the method of Freeman SC et.al. (2022)^3^. In the first stage, each trial was analyzed separately using the Generalized Gamma Model to obtain estimates of the log hazard ratio for the treatment effect and the corresponding standard error. Initially, each trial was independently evaluated by employing the Generalized Gamma Model. This approach facilitated the derivation of log hazard ratio estimates pertinent to the treatment effect, alongside the corresponding standard errors. Formula of Generalized Gamma Model (Q ≠ 0) can be found in section 3.4.1 and we provide it again here.

$$PDF=f(\left. x \right|\mu,\sigma,Q)=\frac{\left| Q \right|(Q^{-2})^{Q^{-2}}}{\sigma t\Gamma(Q^{-2})}\exp[Q^{-2}(Qw-e^{Qw})]$$

In this formula, $\gamma\sim G\text{amma}(Q^{\text{-2}}\text{,1})$, $\omega=\log(Q^{2}\gamma)/Q$, $x=\exp(\mu+\sigma\omega)$, *t* is survival time, *μ* is the location parameter, *σ* is the scale parameter and *Q* is the shape parameter^24^. Please note that in this model, the treatment effect was dependent on the location parameter only. In addition, in this model,

$$\log(t_{ij})=x_{ij}\alpha$$

*x_ij_* is the treatment indicator variable for patient *i* from trial *j*, taking the value 0 if patient i receives the baseline treatment *a* and the value 1 if patient i receives treatment *b*. *μ* is the regression coefficient representing the treatment effect for treatment b compared to the baseline treatment a. In the second stage, the treatment effect estimates of the baseline treatment compared to treatment *i* in trial *j* was synthesized and its variability was estimated within a standard fixed-effect NMA model.

This APP will provide the results of Treatment Effects (compared with the reference treatment). The user can use “*flexsurv*” package to calculate the coefficients (including **mu**, **sigma** and **q**) of applying Generalized Gamma Model on the reference treatment. To be specific, **mu** refers to *μ*, **sigma** refers to *σ,* and **q** refers to Q, respectively. When treatment effects are obtained, users can calculate the survival rate (*S(t)*) through the coefficients and treatment effects. Formula in R can be written as:

S(t)n = 1 - pgengamma(Time, mu = mu + TEn, sigma = sigma, Q=q, ...)

Similarly, hazard (*h(t)*) can be written as:

h(t) = hgengamma(Time, mu = mu + TEn, sigma = sigma, Q=q)

In this APP, estimated time-varying HRs will be estimated based on the calculated treatment effects and the hazard of the reference treatment. To be specific, in the example ALK-NSCLC network, Crizotinib in ALEX will be fitted through a “gengamma” distribution, and the **mu**, **sigma** and **q** will be obtained. Through hazard function and calculated treatment effects, hazards of all the treatments can be calculated. Finally, the HR_12_ can be calculated by h_1_(t)/h_2_(t).

According to Figure 3-18, users can press the button in highlight position 1 so that they can get the results of treatment effects shown in highlight position 2 (NSCLC-ALK network). Users can also modify the settings for JAGS to run the Bayesian analysis. Users should be careful that the results will include both treatment effects and estimated time-varying HRs.

Like COX PH model, users have to prepare the data for running Generalized Gamma Model NMA. Detailed information of the methodology to obtain the data can be found elsewhere^3^. Thus, we do not give any description here. In NMAsurv, this process will be completed by the codes so that users do not need to prepare the data themselves. JAGS code to run the Generalized Gamma Model NMA can be found in the Appendix. An example of data input of Generalized Gamma Model NMA can be found in Table 3-11 (melanoma network). We selected to put the data of melanoma network here since it contains a three-arm study, which can better help to display the data structure.

Table 3‑11 Example of data input of Generalized Gamma Model NMA

| Study | Treatmetn1 | Treatmetn2 | Beta | SE | Covariance | Multi-arm |
| --- | --- | --- | --- | --- | --- | --- |
| BREAK-3 | DB | DTIC | 0.157 | 0.196 | NA | 0 |
| BRIM-3 | VM | DTIC | 0.508 | 0.106 | NA | 0 |
| CheckMate 066 | NIV | DTIC | 0.847 | 0.164 | NA | 0 |
| CheckMate 067 | NIV | IPI | 0.430 | 0.102 | 0.118 | 1 |
| CheckMate 067 | NIV+IPI | IPI | 0.557 | 0.115 | 0.118 | 1 |
| CheckMate 069 | NIV+IPI | IPI | 0.260 | 0.472 | NA | 0 |
| coBRIM | VM+COB | VM | 0.320 | 0.102 | NA | 0 |
| COMBI-d | DB+TR | DB | 0.311 | 0.130 | NA | 0 |
| COMBI-v | VM | DB+TR | -0.234 | 0.090 | NA | 0 |
| Hodi 2014 | IPI+SRG | IPI | 0.368 | 0.206 | NA | 0 |
| Keynote 006 | PEM | IPI | 0.507 | 0.135 | NA | 0 |
| Ribas 2013 | TRL | DTIC | 0.046 | 0.086 | NA | 0 |
| Robert 2011 | IPI+DTIC | DTIC | 0.301 | 0.117 | NA | 0 |
| Robert 2013 | SEL+DTIC | DTIC | 0.156 | 0.181 | NA | 0 |
| CheckMate 067 | IPI | IPI | 0.000 | 0.118 | NA | 1 |
| Note: this example data is generated based on the melanoma network. | | | | | | |

| 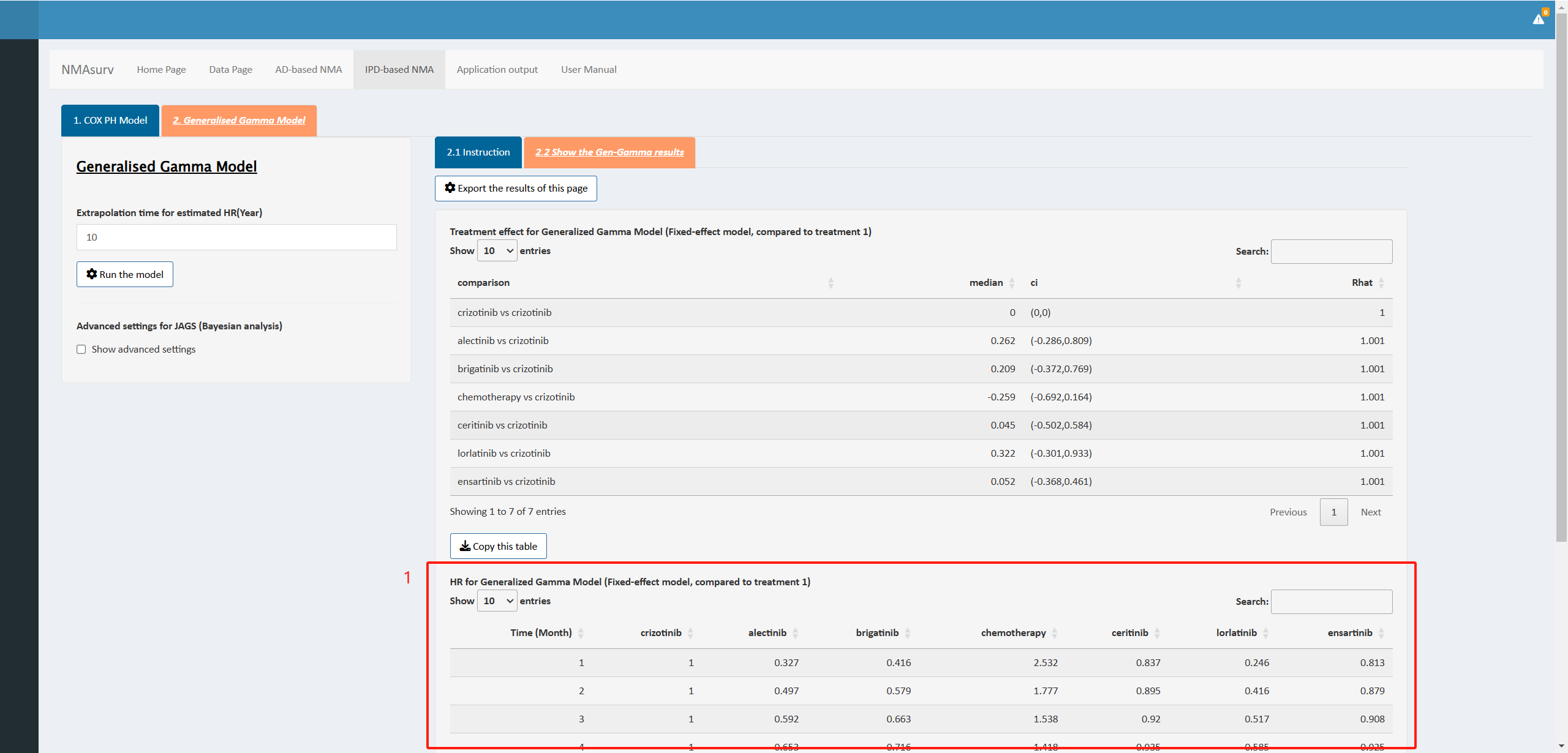 |
| --- |

Figure 3‑18 Section Generalized Gamma Model

## 3.6 Application Output

Given the importance of selecting an appropriate NMA model for the estimation of time-varying relative treatment effects to be used in evidence-based medicine or cost-effectiveness analysis, users should pay more attention to this process. Typically, users have to select the models within NMA methods and between NMA methods. In NMAsurv, we will not provide the conclusions to tell the users which model performed best. However, we provide several other functions to help users to make their own decisions: (1) statistical indicators and plots for users to select the models within NMA methods; (2) extrapolated HRs and results batch export for users to generate their reports and make the final comparison; (3) some useful notes and references for users to get familiar with model selection quickly. In “Application Output” module, NMAsurv provides three functions: “Notes on model selection”, “Notes on HRs report”, and “Output Report”. The first two are useful notes and references while the third one is a panel for users to export results in batches. Please note that the notes in the app are similar to the ones provided here; however, here we include more detailed information.

### 3.6.1 Notes on model selection

**Model selection for specific NMA methods**

For the FP model, through the step one frequentist analysis, the best models with the lowest AIC can be obtained. But be aware that additional step two analysis should be conducted because overfit may exist in models with the lowest AIC. A check for hazard, survival and HR plots is needed before making a conclusion on the final model. For example, take NSCLC-ALK network as an example, for FP, the best models are FP1 with power = 2,3,1. Before we made the final conclusion, we conducted an additional visual inspection for survival curves. We found that FP1 with power = 2 and 3, although had the lowest AIC, showed different degrees of overfitting in the middle or later part of the extrapolation. Details can be found in the highlighted areas in Figure 3-19 (a) and (b). Thus, FP1 with power = 1 was selected at last. For other models, overfitting can also be observed. Therefore, visual inspection is highly recommended to be conducted instead of using statistical indicators only.

| (a) FP1, power = 2  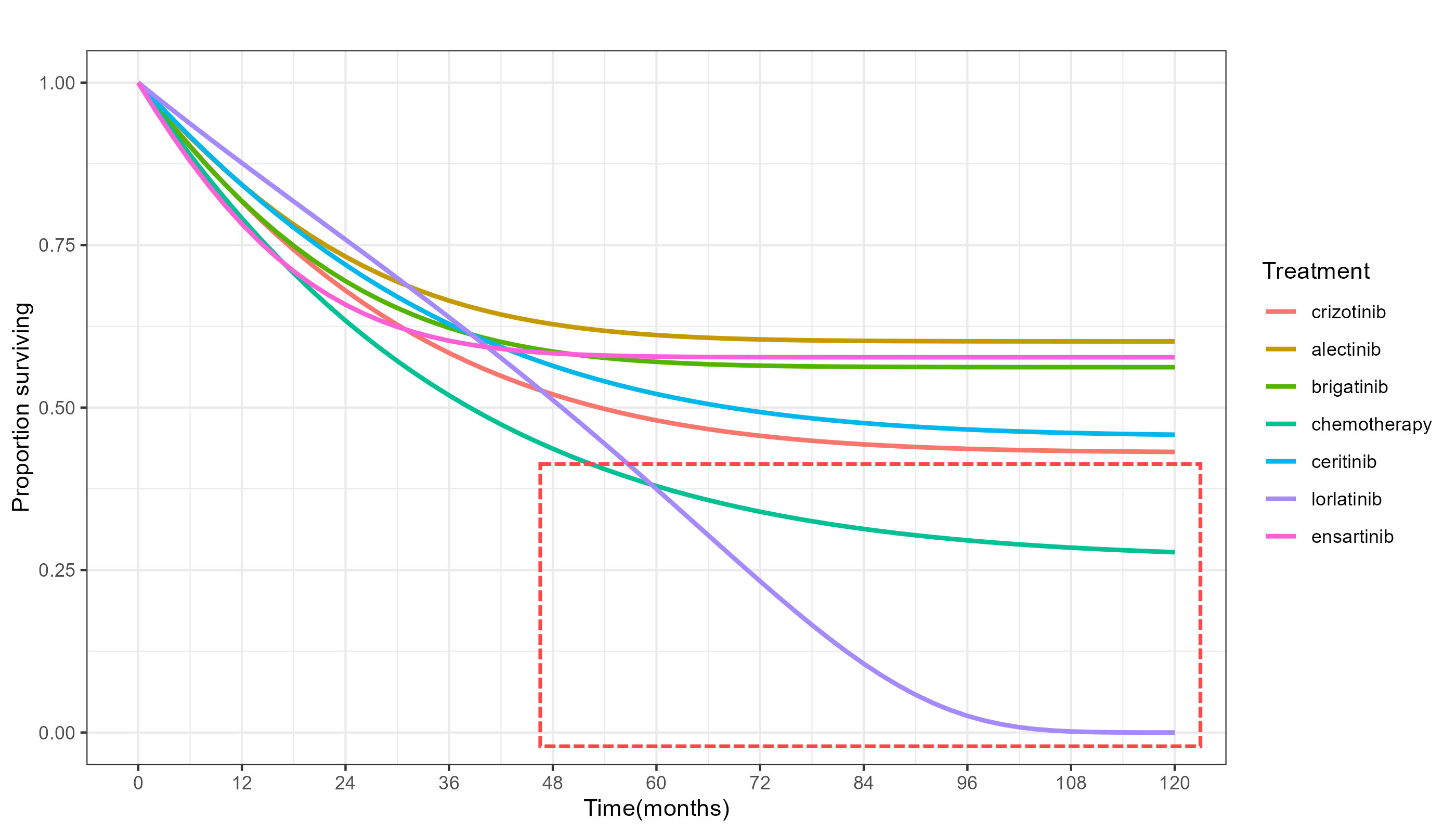 |
| --- |
| (b) FP1, power = 3  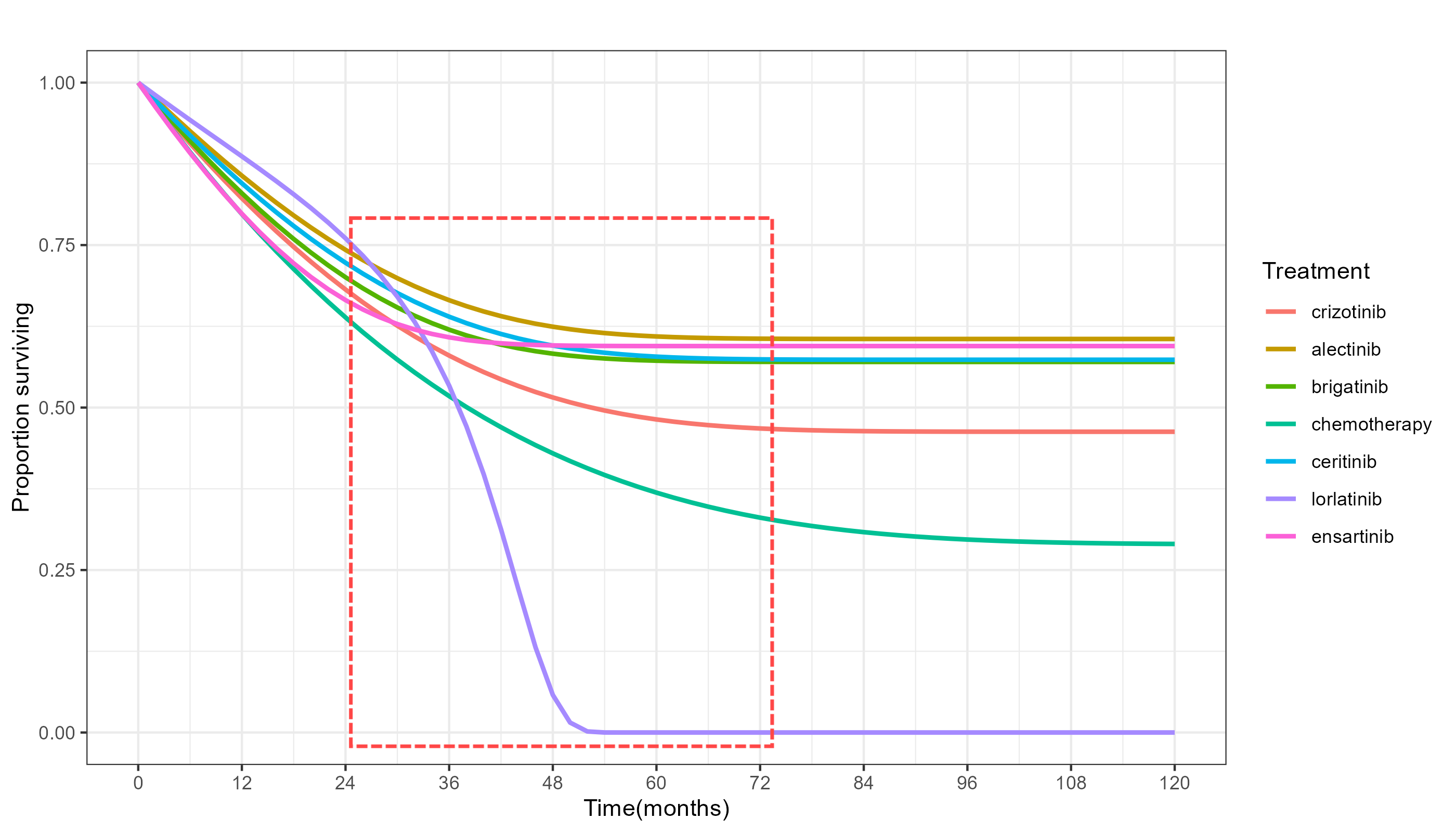 |

Figure 3‑19 Survival Plots for FP example

For the PWE model, the most important part is to select the most suitable cutpoints. Here are some suggestions on the selection of potential cutpoints: (1) the time that most survival curves show a plateau period; (2) the time that there are turning points on hazard plots. In this APP, the AIC of PWE can be compared with FP. This is because in NMAsurv, the generalized linear model framework is used for both models. Therefore, through comparing AIC, users can select the best model with statistical performance among all FP and PWE models.

For the PSM, a selection of the best model can be conducted based on the comparison of DIC. However, users should pay attention that not all parametric survival distribution may be suitable for the given data. For example, see the example data (NSCLC-ALK network), Log-normal distribution had a poor fit on the data (Figure 3-20).

| 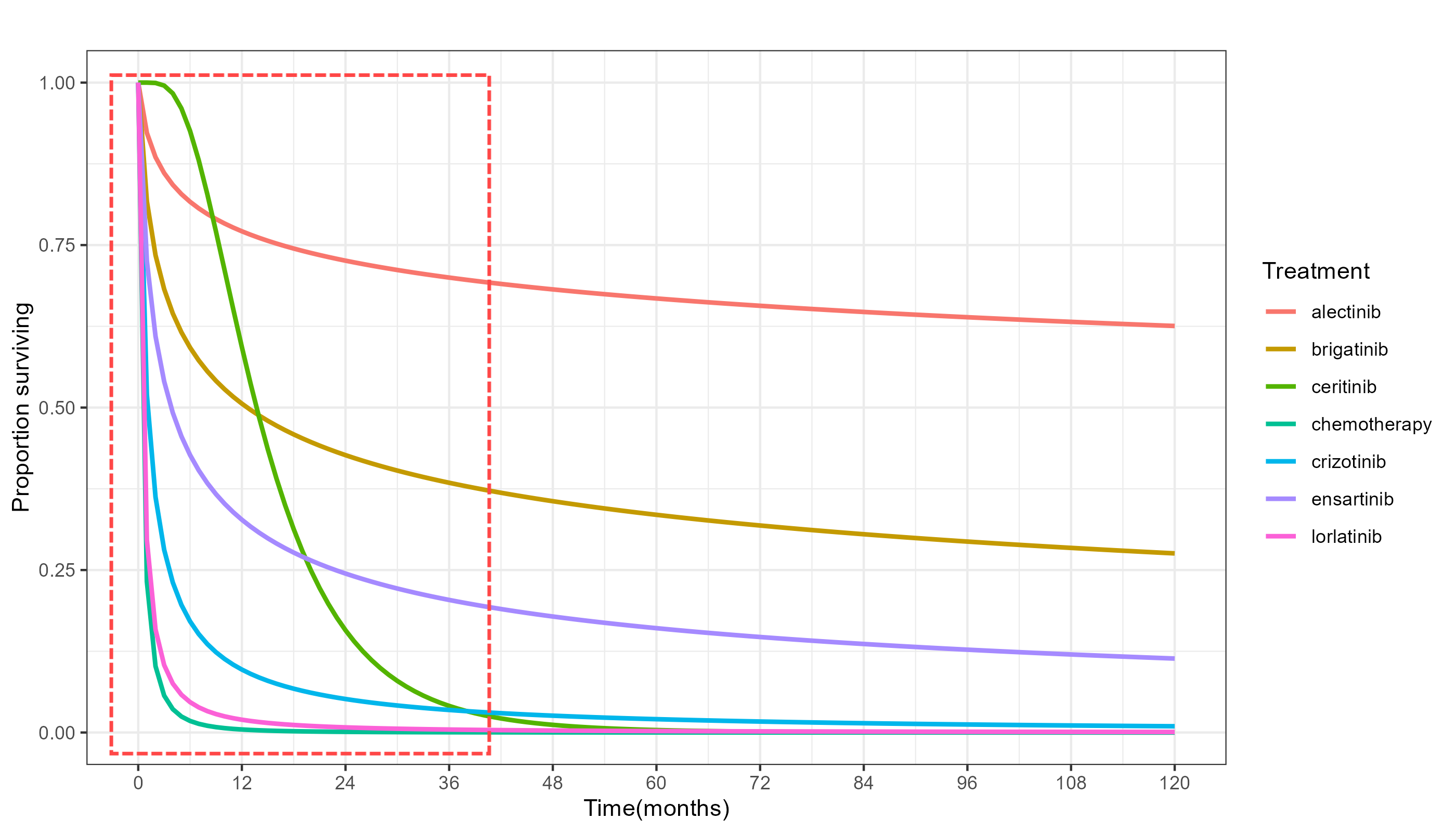 |
| --- |

Figure 3‑20 Survival Plots for PSM example

For the COX PH Model and Gen Gamma Model, there is no need for model selection within models since there will be only one model generated for these two methods.

Regarding the problems mentioned before about overfitting and poor model fitting performance, one possible reason is that the data used is immature. For survival extrapolation, if the data is immature, it will lead to unreliable predictions^25^.

**Model selection among different NMA methods**

There is currently no authoritative standard for how to select the best model among different NMA models. Comparisons between NMA models are often difficult to achieve due to different methodological compositions. Using different research data may also lead to large changes in choices for models. However, there are many published studies including frameworks and suggestions on selecting NMA models in this field. Researchers can benefit a lot from these published studies. Some useful references can be found elsewhere^3,11,26^. Here we want to mention the study published by Cope et.al. (2023). This study compared four non-PH NMA methods (one-step or two-step NMA based on traditional survival distributions or FP, restricted cubic splines (RCS) based NMA, and RMST based NMA) through an oncology case study, demonstrating that FP and RCS provided the best data fit for PFS. While HRs across methods were similar at 5 years, they diverged in long-term extrapolations, and non-PH methods captured uncertainties in relative treatment effects over time. The authors recommend using non-PH NMA methods when PH assumptions are questionable, employing a transparent model selection process and sensitivity analyses to ensure robust results. They also come up with a model selection process which can be great useful for researchers who want to conduct an NMA with a rigorous process.

In NMAsurv, users can batch export the results (images and tables) of their selected models into a Word document to facilitate their comparison of results or report writing. Although the results or conclusions on which model performs best will not be provided in this APP, developers can provide some tricks to help the researchers to make the whole model selection process more rigorous and comprehensive here:

(1) Although the choice between different NMA models may be difficult at times, it is a good idea to make the entire model selection process transparent and justifiable with the help of existing model selection frameworks.

(2) In addition to statistical indicators, visual inspection through plots is also very important. Some estimates that are obviously counterintuitive can be easily discovered through visual inspection.

(3) External evidence and clinical validity will be good evidence to support model choice if available.

(4) Sensitivity analyses are highly suggested due to the inherent uncertainty, especially for economic evaluation. Considering different models in sensitivity analyses should be given priority instead of using only one NMA model in the study.

### 3.6.2 Notes on HRs output

For users who want to do the cost-effectiveness analysis of multiple treatments, they have to conduct the NMA to get the relative treatment effects. Typically, the relative treatment effects can be the HRs. For time-varing HRs, they should have the same follow-up time as the cost-effectiveness analysis. This means that extrapolation on HRs is needed. In this APP, for each model except COX PH model (time-constant HRs), we provide the output of estimated HR with the time duration determined by the users. However, users should note that sometimes the extrapolation of time-varing HRs is not reliable, especially when the data used to run the NMA model is immature. Under this condition, continuing to use time-varing HRs to extrapolate may lead to greater biases than using constant HRs (Based on the results of one unpublished article). Some CEA studies have tried to solve this by using an alternative extrapolation method^27,28^. The details of this method can be found as follows:

(1) Calculate the (average) time duration with mature survival data for each treatment based on the method of Gebski et.al. (2018)^29^. The following is a brief description of the method, which is directly obtained from the original paper^29^:

“*We propose methods to determine the minimum number of subjects remaining at risk after which Kaplan-Meier survival plots for time-to-event outcomes should be curtailed, as, once the number remaining at risk drops below this minimum, the survival estimates are no longer meaningful in the context of the investigation. The size of the decrease of the Kaplan Meier survival estimate S(t) at time t if one extra event should occur is considered in two ways. In the first approach, the investigator sets a maximum acceptable absolute decrease in S(t) should one extra event occur. In the second, a minimum acceptable number of subjects still at risk is calculated by comparing the size of the decrease in S(t) if an extra event should occur with the variability of the survival estimate had all subjects been followed to that time (confidence interval approach).*”

(2) For each treatment, for time with mature survival data, use the model estimated HRs as the results; for time with immature survival data, use the HR of the last time point of the mature data as the result, and extrapolate the HR for the remaining time by this constant HR value.

Here is a simple example to illustrate this method: For treatment A, the average time to have mature data is 12 months, but we want to get the extrapolated HRs of 36 months. Thus, we set the time point as 12 months. Before the 12th month, we used the model estimated time-varing HRs. After the 12th month, we used the HR of the 12th month to extrapolate the HRs to the 36th month. That is, between the 13th month and the 36th month, the HRs are constant.

Although this method sometimes can be quite useful to solve the problem of immature data, currently, there is no authoritative methodological research available to validate whether using the last HR of mature data to extrapolate is a reasonable approach. Future studies are needed to justify this method.

### 3.6.3 Instruction on “Output Report” panel

In 'Output Report' panel, exported outcomes including coefficient tables, survival plots, hazard plots and HR plots will be shown here. All selected outcomes can be merged into one word document. Users can select which outcome to report in their final word report through the checkboxes as shown in Figure 3-21 (a). Extrapolated HR data will not be provided here. Also please note that only successfully exported model outcomes will be shown here. (Pressing the export data button and getting a success message is considered a successful attempt, as shown in Figure 3-21 (b)). An example of output report for NSCLC-ALK network with two models ((1) FP1, Power=1, Frequentist analysis, Fixed-effect model and (2) Cox-PH Model, Bayesian analysis, Fixed-effect model) can be found in Appendix.

| (a)  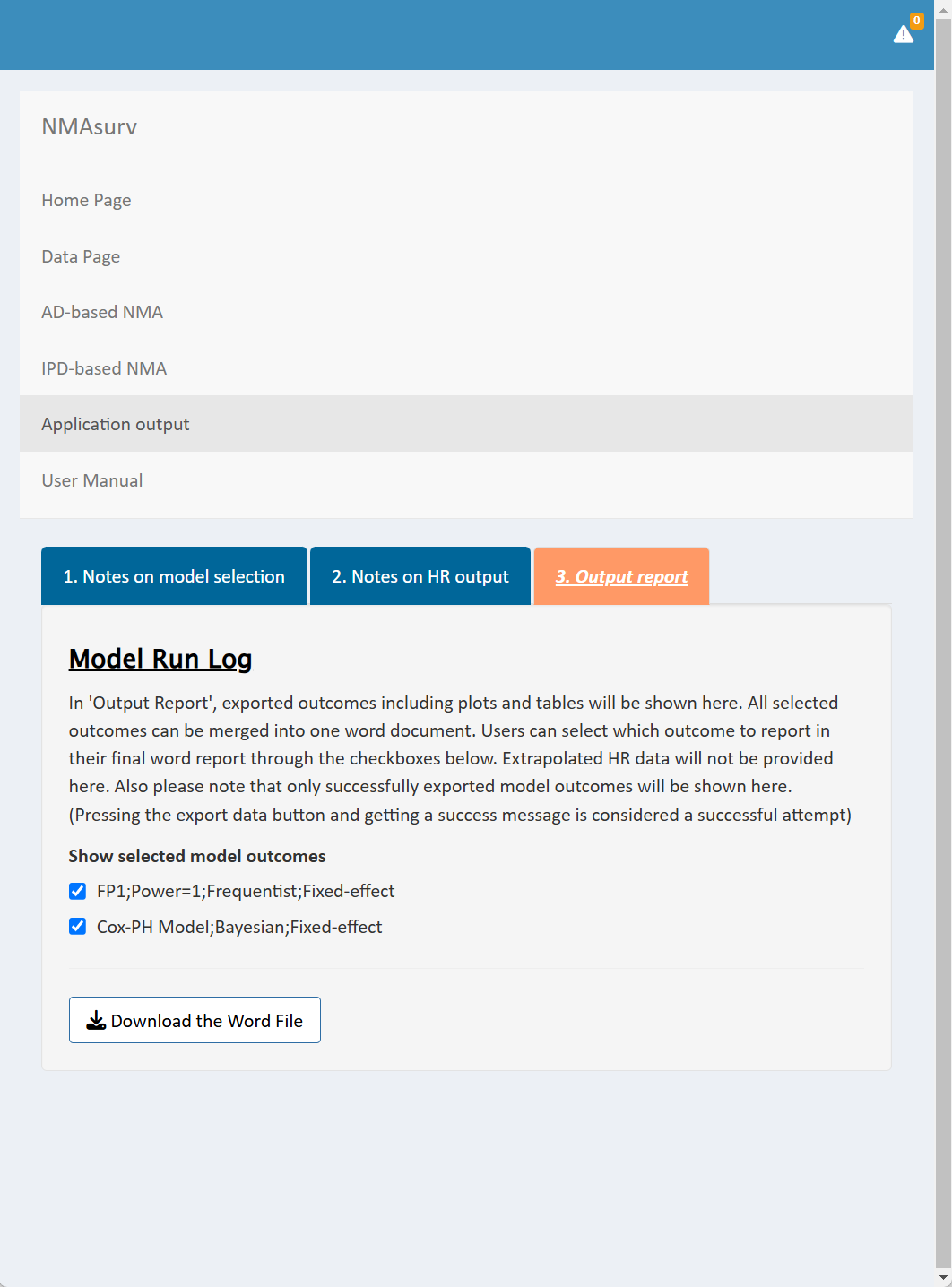 | (b) 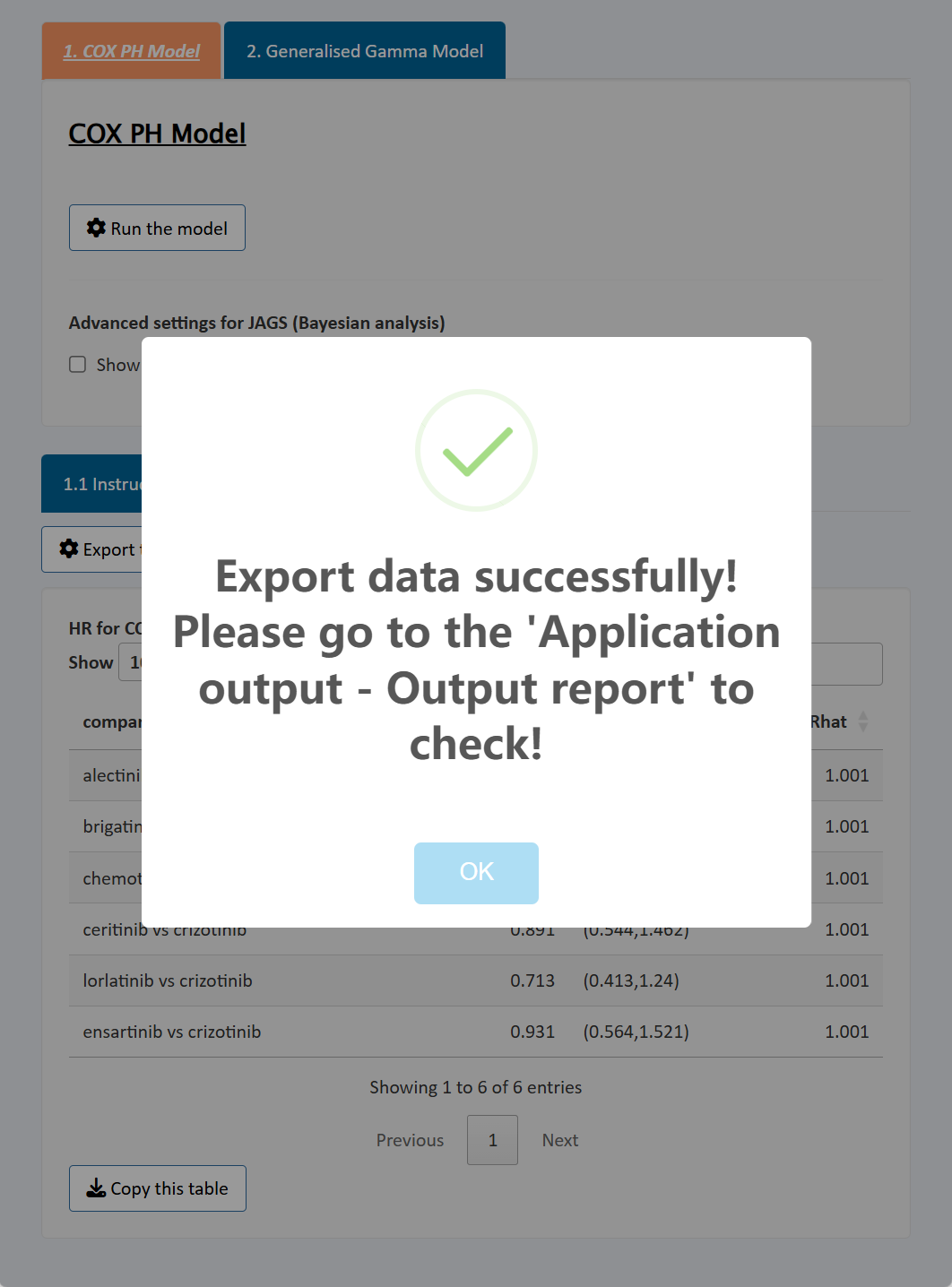 |
| --- | --- |

Figure 3‑21 Example of successful result export

# Tips for users when using this APP

Before using NMAsurv, developers recommend users to read these tips. Firstly, when opening the APP, if the content is not displayed well, users can adjust the zoom ratio of the web page display. For example, if the monitor size is 27 inches and the resolution is 2560*1440, a suitable zoom ratio will be 100% to 125%.

Secondly, when encountering errors, users can check the following items: (1) whether the data has been correctly inputted. (2) Check the parameters input. (3) Check the data format and the content of the data. (4) Check the settings of JAGS. (5) There are some bugs in this APP. Bugs can be reported to the developers through email. If bugs can be found, we will be very grateful.

Thirdly, users should be aware that this APP is designed to conduct NMA based on reconstructed survival data. Although users can get results without programming through this APP, they can program themselves if they have other special requirements. In addition, this tool will not report any results for choosing models among different kinds of models. Users should take rigorous and comprehensive steps to reduce the uncertainty. When building NMA models, published frameworks and guidelines should be paid more attention to^10,11^.

Finally, although users can use this APP without mastering the methodology of survival analysis and NMA, we strongly recommend users to learn some basic knowledge to avoid selecting inappropriate models or obtaining incorrect results. Useful learning materials can be found through the instruction part of each module and the references in the homepage.

# Reference

1. Zhao M, Shao T, Shao H, Zhou C, Tang W. Identifying optimal ALK inhibitors in first- and second-line treatment of patients with advanced ALK-positive non-small-cell lung cancer: a systematic review and network meta-analysis. BMC Cancer. 2024;24(1):186

2. Guyot P, Ades AE, Ouwens MJ, Welton NJ. Enhanced secondary analysis of survival data: reconstructing the data from published Kaplan-Meier survival curves. BMC Med Res Methodol. 2012;12:9

3. Freeman SC, Cooper NJ, Sutton AJ et al. Challenges of modelling approaches for network meta-analysis of time-to-event outcomes in the presence of non-proportional hazards to aid decision making: Application to a melanoma network. Stat Methods Med Res. 2022;31(5):839-861

4. Zoratti MJ, Devji T, Levine O, Thabane L, Xie F. Network meta-analysis of therapies for previously untreated advanced BRAF-mutated melanoma. Cancer Treat Rev. 2019;74:43-48

5. Wiksten A, Hawkins N, Piepho HP, Gsteiger S. Nonproportional Hazards in Network Meta-Analysis: Efficient Strategies for Model Building and Analysis. Value Health. 2020;23(7):918-927

6. Jansen JP. Network meta-analysis of survival data with fractional polynomials. BMC Med Res Methodol. 2011;11:61

7. Shao T, Zhao M, Liang L, Tang W. A systematic review and network meta-analysis of first-line immune checkpoint inhibitor combination therapies in patients with advanced non-squamous non-small cell lung cancer. Front Immunol. 2022;13:948597

8. Jrgensen SE. Model Selection and Multimodel Inference. Ecol Modell. 2004;

9. Spiegelhalter DJ, Best NG, Carlin BP, Van Der Linde A. Bayesian measures of model complexity and fit. Journal of the Royal Statistical Society: Series B (Statistical Methodology). 2002;64(4):583-639

10. CHTE2020 SOURCES AND SYNTHESIS OF EVIDENCE; UPDATE TO EVIDENCE SYNTHESIS METHODS. 2020;2022(14 April)

11. Cope S, Chan K, Campbell H et al. A Comparison of Alternative Network Meta-Analysis Methods in the Presence of Nonproportional Hazards: A Case Study in First-Line Advanced or Metastatic Renal Cell Carcinoma. Value Health. 2023;26(4):465-476

12. Cope S, Chan K, Campbell H et al. A Comparison of Alternative Network Meta-Analysis Methods in the Presence of Nonproportional Hazards: A Case Study in First-Line Advanced or Metastatic Renal Cell Carcinoma. Value Health. 2023;26(4):465-476

13. Bowden J, Tierney JF, Simmonds M, Copas AJ, Higgins JP. Individual patient data meta-analysis of time-to-event outcomes: one-stage versus two-stage approaches for estimating the hazard ratio under a random effects model. Res Synth Methods. 2011;2(3):150-62

14. SCHOENFELD D. Partial residuals for the proportional hazards regression model. Biometrika. 1982;69(1):239-241

15. GRAMBSCH PM, THERNEAU TM. Proportional hazards tests and diagnostics based on weighted residuals. Biometrika. 1994;81(3):515-526

16. Royston P, Altman DG. Regression Using Fractional Polynomials of Continuous Covariates: Parsimonious Parametric Modelling. Insurance Mathematics and Economics. 1995;16(2):165-166

17. Royston P, Parmar MK. Flexible parametric proportional-hazards and proportional-odds models for censored survival data, with application to prognostic modelling and estimation of treatment effects. Stat Med. 2002;21(15):2175-97

18. Aki V, Andrew G, Daniel S, Bob C, Paul-Christian B. Rank-Normalization, Folding, and Localization: An Improved $\widehat{R}$ for Assessing Convergence of MCMC (with Discussion). Bayesian Anal. 2021;16(2):667-718

19. Li Y, Gail MH, Preston DL, Graubard BI, Lubin JH. Piecewise exponential survival times and analysis of case-cohort data. Stat Med. 2012;31(13):1361-8

20. Amzal B, Fu S, Meng J, Lister J, Karcher H. Cabozantinib versus everolimus, nivolumab, axitinib, sorafenib and best supportive care: A network meta-analysis of progression-free survival and overall survival in second line treatment of advanced renal cell carcinoma. PLoS One. 2017;12(9):e0184423

21. Wiecek W, Karcher H. Nivolumab versus Cabozantinib: Comparing Overall Survival in Metastatic Renal Cell Carcinoma. PLoS One. 2016;11(6):e0155389

22. Ouwens MJ, Philips Z, Jansen JP. Network meta-analysis of parametric survival curves. Res Synth Methods. 2010;1(3-4):258-71

23. Harrell FE. Cox Proportional Hazards Regression Model. 2001:465-507

24. Cox C, Chu H, Schneider MF, Munoz A. Parametric survival analysis and taxonomy of hazard functions for the generalized gamma distribution. Stat Med. 2007;26(23):4352-74

25. Shao T, Zhao M, Liang L, Shi L, Tang W. Impact of Extrapolation Model Choices on the Structural Uncertainty in Economic Evaluations for Cancer Immunotherapy: A Case Study of Checkmate 067. Pharmacoecon Open. 2023;7(3):383-392

26. Heeg B, Garcia A, Beekhuizen SV et al. Novel and existing flexible survival methods for network meta-analyses. J Comp Eff Res. 2022;

27. Pei R, Shi Y, Lv S et al. Nivolumab vs Pembrolizumab for Treatment of US Patients With Platinum-Refractory Recurrent or Metastatic Head and Neck Squamous Cell Carcinoma: A Network Meta-analysis and Cost-effectiveness Analysis. JAMA Netw Open. 2021;4(5):e218065

28. Wang L, Hong H, Alexander GC et al. Cost-Effectiveness of Systemic Treatments for Metastatic Castration-Sensitive Prostate Cancer: An Economic Evaluation Based on Network Meta-Analysis. Value Health. 2022;25(5):796-802

29. Gebski V, Gares V, Gibbs E, Byth K. Data maturity and follow-up in time-to-event analyses. Int J Epidemiol. 2018;47(3):850-859

# Appendix

## 6.1 JAGS codes

### 6.1.1 JAGS codes for running FP2 NMA (fixed-effect model)

model{

## Sampling model

for (i in 1:Nobs){

time1[i] <- (equals(P1,0) * log(time[i]) + (1-equals(P1,0)) * pow(time[i],P1) )

time2[i] <- ( (1-equals(P2,P1)) * ( equals(P2,0) * log(time[i]) + (1-equals(P2,0)) * pow(time[i],P2) ) +

equals(P2,P1) * ( equals(P2,0) * log(time[i])*log(time[i]) + (1-equals(P2,0)) * pow(time[i],P2) * log(time[i]) ) )

}

for (i in 1:Nobs){

# likelihood: digitized KM curves, grouped into intervals [t, t+dt]

r[i] ~ dbin(p[i], n[i])

p[i] <- 1 - exp(-h[i] * dt[i]) # cumulative hazard over interval [t,t+dt] expressed as deaths per person-month

# fractional polynomial

log(h[i]) <- Beta[s[i], a[i], 1] + Beta[s[i], a[i], 2] * time1[i] + Beta[s[i], a[i], 3] * time2[i]

}

## Arm level parameters = study effect + trt effect (consistency eq)

for (l in 1:Ns){

for (ll in 1:Na[l]){

Beta[l, ll, 1] <- mu[l, 1] + d[t[l, ll], 1] - d[t[l, 1], 1]

Beta[l, ll, 2] <- mu[l, 2] + d[t[l, ll], 2] - d[t[l, 1], 2]

Beta[l, ll, 3] <- mu[l, 3] + d[t[l, ll], 3] - d[t[l, 1], 3]

}

}

## Priors

for (j in 1:Ns){

mu[j, 1:3] ~ dmnorm(prior.mean[1:3], prior.prec[,])

}

d[1, 1] <- 0

d[1, 2] <- 0

d[1, 3] <- 0

for (k in 2:Ntrt){

d[k, 1:3] ~ dmnorm(prior.mean[1:3], prior.prec[,])

}

} # end of model

### 6.1.2 JAGS codes for running FP2 NMA (random-effects model, d0)

model{

for (i in 1:Nobs){

time_transf1[i]<-(equals(P1,0)*log(time[i]) + (1-equals(P1,0))*pow(time[i],P1))

time_transf2[i]<-((1-equals(P2,P1))*(equals(P2,0)*log(time[i]) + (1-equals(P2,0))*pow(time[i],P2)) + equals(P2,P1)*(equals(P2,0)*log(time[i])*log(time[i])+ (1-equals(P2,0))*pow(time[i],P2) *log(time[i])))

# likelihood

r[i] ~ dbin(p[i], n[i])

p[i] <- 1 - exp(-h[i] * dt[i])

log(h[i])<-Beta[i,1]+ Beta[i,2]*time_transf1[i]+ Beta[i,3]* time_transf2[i]

Beta[i,1]<-mu[s[i],1]+delta[s[i],1]*(1-equals (k[i], b[i]))

Beta[i,2]<-mu[s[i],2]+(d[ks[s[i]],2]-d[bs[s[i]],2])*(1-equals (k[i], b[i]))

Beta[i,3]<-mu[s[i],3]+(d[ks[s[i]],3]-d[bs[s[i]],3])*(1-equals (k[i], b[i]))

}

theta <- pow( sd , 2 )

for(m in 1:Ns){

delta[m,1]~dmnorm( md[ m, 1], theta )

md[m,1]<-d[ks[m],1]-d[bs[m],1]

}

# priors

d[1,1]<-0

d[1,2]<-0

d[1,3]<-0

for(j in 2:Ntrt){

d[j,1:3] ~ dmnorm(prior.mean[ 1 : 3 ], prior.prec[ , ])

}

for(k in 1:Ns){

mu[k,1:3] ~ dmnorm(prior.mean[ 1 : 3 ], prior.prec[ , ])

}

sd ~ dunif(0,2)

}

### 6.1.3 JAGS codes for running PWE with two cutpoints

model{

for (i in 1:Nobs){

# likelihood: digitized KM curves

r[i] ~ dbin(p[i], n[i])

p[i] <- 1 - exp(-h[i] * dt[i])

# piecewise constant model

log(h[i]) <- Beta[s[i], a[i], segment[i]]

}

for (i in 1:Ns){

for (j in 1:Na[i]){

Beta[i, j, 1] <- mu[i, 1] + d[t[i, j], 1] - d[t[i, 1], 1]

for (k in 2:(Ncuts + 1)){

Beta[i, j, k] <- mu[i, k] + d[t[i, j], k] - d[t[i, 1], k]-(mu[i, 1] + d[t[i, j], 1] - d[t[i, 1], 1])

}

}

}

## Priors

for (i in 1:Ns){

for (k in 1:(Ncuts + 1)){

mu[i, k] ~ dnorm(prior.mean, prior.prec)

}

}

for (k in 1:(Ncuts + 1)){

d[1, k] <- 0

for (i in 2:Ntrt){

d[i, k] ~ dnorm(prior.mean, prior.prec)

}

}

} # end of model

### 6.1.4 JAGS codes for running fixed-effect Weilbull

model{

for (i in 1:Nobs){

# likelihood

r[i] ~ dbin(p[i], n[i])

p[i] <- 1 - exp(-h[i] * dt[i])

log(h[i])<-Beta[i,1]+ Beta[i,2]*log(time[i])

Beta[i,1]<-mu[s[i],1]+md[s[i],1]*(1-equals (k[i], b[i]))

Beta[i,2]<-mu[s[i],2]+md[s[i],2]*(1-equals (k[i], b[i]))

}

for(k in 1 :Ns){

md[k,1]<-d[ks[k],1]-d[bs[k],1]

md[k,2]<-d[ks[k],2]-d[bs[k],2]

}

# priors

d[1,1]<-0

d[1,2]<-0

for(j in 2 :Ntrt){

d[j,1:2] ~ dmnorm(mean[1:2],prec2[,])

}

for(k in 1 :Ns){

mu[k,1:2] ~ dmnorm(mean[1:2],prec2[,])

}

}

### 6.1.5 JAGS codes for running fixed-effect Gompertz

model{

for (i in 1:Nobs){

# likelihood

r[i] ~ dbin(p[i], n[i])

p[i] <- 1 - exp(-h[i] * dt[i])

log(h[i])<-Beta[i,1]+ Beta[i,2]*time[i]

Beta[i,1]<-mu[s[i],1]+md[s[i],1]*(1-equals (k[i], b[i]))

Beta[i,2]<-mu[s[i],2]+md[s[i],2]*(1-equals (k[i], b[i]))

}

for(k in 1 :Ns){

md[k,1]<-d[ks[k],1]-d[bs[k],1]

md[k,2]<-d[ks[k],2]-d[bs[k],2]

}

# priors

d[1,1]<-0

d[1,2]<-0

for(j in 2 :Ntrt){

d[j,1:2] ~ dmnorm(mean[1:2],prec2[,])

}

for(k in 1 :Ns){

mu[k,1:2] ~ dmnorm(mean[1:2],prec2[,])

}

}

### 6.1.6 JAGS codes for running fixed-effect Log-normal

model{

for (i in 1:Nobs){

# likelihood

r[i] ~ dbin(p[i], n[i])

p[i]<-1-exp(-h[i]*dt[i])

h[i]<-pow(2*3.1415926,-0.5)*exp(-pow((log(time[i])-nu[i])/exp(theta[i]),2)*0.5) / (exp(theta[i])*time[i]*phi(-(log(time[i])-nu[i])/exp(theta[i])))

nu[i]<-mu[s[i],1]+md[s[i],1]*(1-equals (k[i], b[i]))

theta[i]<-mu[s[i],2]+md[s[i],2]*(1-equals (k[i], b[i]))

}

for(k in 1 :Ns){

md[k,1]<-d[ks[k],1]-d[bs[k],1]

md[k,2]<-d[ks[k],2]-d[bs[k],2]

}

# priors

d[1,1]<-0

d[1,2]<-0

for(j in 2 :Ntrt){

d[j,1:2] ~ dmnorm(mean[1:2],prec2[,])

}

for(k in 1 :Ns){

mu[k,1:2] ~ dmnorm(mean[1:2],prec2[,])

}

}

### 6.1.7 JAGS codes for running fixed-effect Log-logistic

model{

for (i in 1:Nobs){

# likelihood

r[i] ~ dbin(p[i], n[i])

p[i]<-1-exp(-h[i]*dt[i])

h[i]<-(exp(theta[i])/exp(nu[i]))*pow(time[i]/exp(nu[i]), exp(theta[i])-1)/(1+pow(time[i]/exp(nu[i]), exp(theta[i])))

nu[i]<-mu[s[i],1]+md[s[i],1]*(1-equals (k[i], b[i]))

theta[i]<-mu[s[i],2]+md[s[i],2]*(1-equals (k[i], b[i]))

}

for(k in 1 :Ns){

md[k,1]<-d[ks[k],1]-d[bs[k],1]

md[k,2]<-d[ks[k],2]-d[bs[k],2]

}

# priors

d[1,1]<-0

d[1,2]<-0

for(j in 2 :Ntrt){

d[j,1:2] ~ dmnorm(mean[1:2],prec2[,])

}

for(k in 1 :Ns){

mu[k,1:2] ~ dmnorm(mean[1:2],prec2[,])

}

}

### 6.1.8 JAGS codes for running fixed-effect COX PH NMA

model{

for(i in 1:ns2) {

y[i,2] ~ dnorm(delta[i,2],prec[i,2])

resdev[i] <- (y[i,2]-delta[i,2])*(y[i,2]-delta[i,2])*prec[i,2]

}

for(i in (ns2+1):(ns2+ns3)) {

for (k in 1:(na[i]-1)) {

for (j in 1:(na[i]-1)) {

Sigma[i,j,k] <- V[i]*(1-equals(j,k)) + var0[i,k+1]*equals(j,k)

}

}

Omega[i,1:(na[i]-1),1:(na[i]-1)] <- inverse(Sigma[i,,])

y[i,2:na[i]] ~ dmnorm(delta[i,2:na[i]],Omega[i,1:(na[i]-1),1:(na[i]-1)])

for (k in 1:(na[i]-1)){

ydiff[i,k]<- y[i,(k+1)] - delta[i,(k+1)]

z[i,k]<- inprod(Omega[i,k,1:(na[i]-1)], ydiff[i,1:(na[i]-1)])

}

resdev[i]<- inprod(ydiff[i,1:(na[i]-1)], z[i,1:(na[i]-1)])

}

for(i in 1:(ns2+ns3)){

for (k in 2:na[i]) {

var0[i,k] <- pow(se[i,k],2)

prec[i,k] <- 1/var0[i,k]

delta[i,k] <- d[t[i,k]] - d[t[i,1]]

}

}

totresdev <- sum(resdev[])

d[1]<-0

for (k in 2:nt){

d[k] ~ dnorm(0,0.0001)

}

for (k in 2:nt){

hrd[k] <- exp(d[k])

}

}

### 6.1.9 JAGS codes for running fixed-effect Generalized Gamma Model NMA

model {

#Define Prior Distributions

beta[1] <- 0

for (tt in 2:nTx){

beta[tt]~dnorm(0,1.0E-4)

}

for(ss in 1:nStudies){

alpha[ss] ~ dnorm(0,1.0E-4)

}

for(ii in 1:LnObs ){

Lmu[ii] <- alpha[Lstudy[ii]]*multi[ii] + beta[Ltx[ii]] - beta[Lbase[ii]]

Lprec[ii] <- 1/pow(Lse[ii],2)

Lmean[ii] ~ dnorm(Lmu[ii],Lprec[ii])

}

# Calculate AFT

for (hh in 1:nTx) {

aft[hh] <- exp(beta[hh])

}

}

## 6.2 Output report example for NSCLC-ALK network

**FP1;Power=1;Frequentist;Fixed-effect**

HR Plot


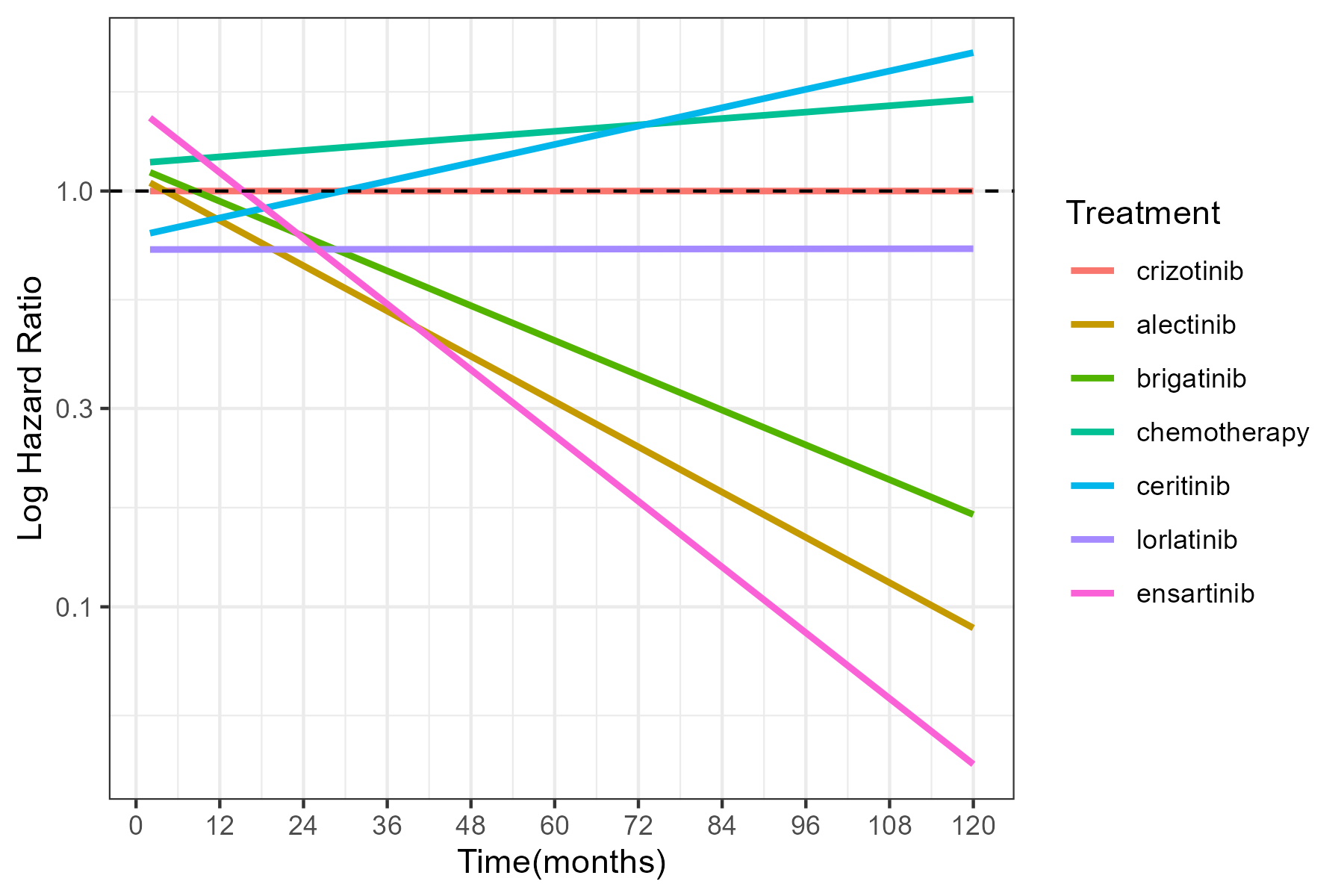


Survival Plot


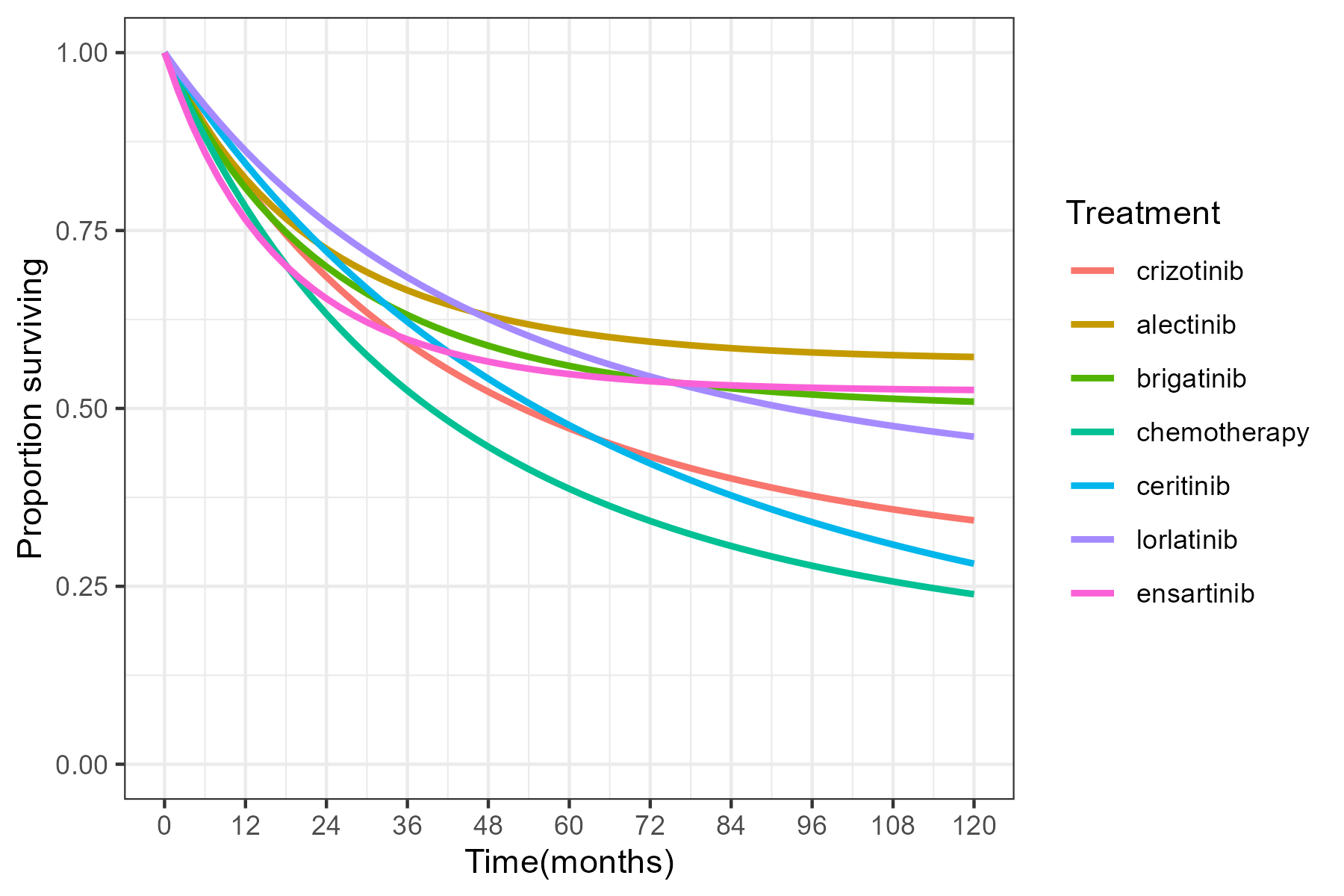


Hazard Plot


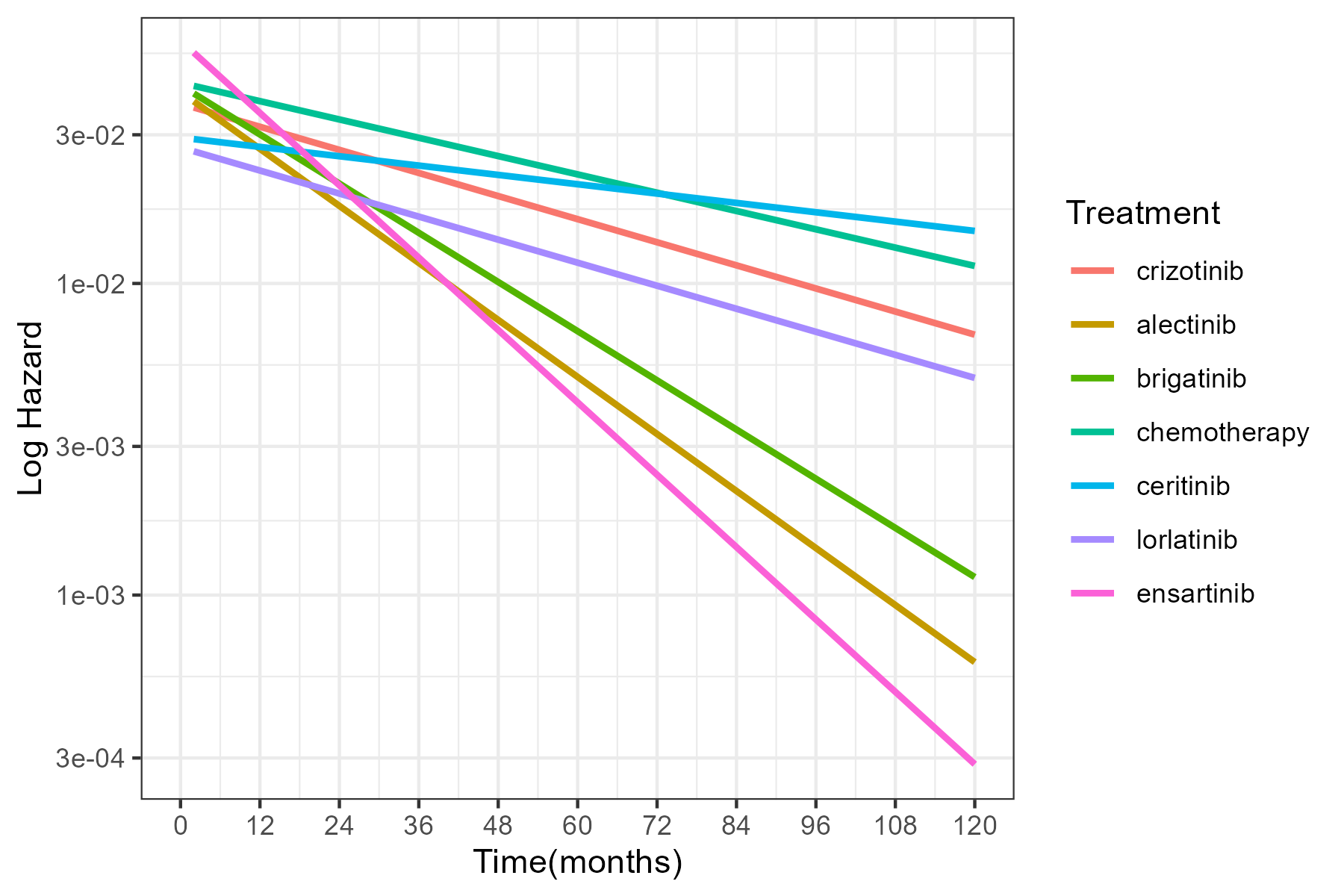


Coefficient Table (Treatment effects)

| Treatment | est | std.err | conf.int.lower | conf.int.upper |
| --- | --- | --- | --- | --- |
| alectinib | 0.088 | 0.330 | -0.560 | 0.735 |
| brigatinib | 0.136 | 0.388 | -0.625 | 0.897 |
| chemotherapy | 0.154 | 0.284 | -0.403 | 0.711 |
| ceritinib | -0.250 | 0.515 | -1.260 | 0.759 |
| lorlatinib | -0.324 | 0.588 | -1.476 | 0.828 |
| ensartinib | 0.466 | 0.552 | -0.616 | 1.549 |
| alectinib:f1 | -0.021 | 0.013 | -0.047 | 0.005 |
| brigatinib:f1 | -0.016 | 0.016 | -0.048 | 0.016 |
| chemotherapy:f1 | 0.003 | 0.012 | -0.020 | 0.026 |
| ceritinib:f1 | 0.008 | 0.030 | -0.051 | 0.068 |
| lorlatinib:f1 | 0.000 | 0.039 | -0.075 | 0.075 |
| ensartinib:f1 | -0.030 | 0.029 | -0.086 | 0.026 |

**Cox-PH Model;Bayesian;Fixed-effect**

Coefficient Table (HR)

| Treatment | comparison | median | ci | Rhat |
| --- | --- | --- | --- | --- |
| hrd[2] | alectinib vs crizotinib | 0.704 | (0.485,1.008) | 1.001 |
| hrd[3] | brigatinib vs crizotinib | 0.830 | (0.559,1.251) | 1.001 |
| hrd[4] | chemotherapy vs crizotinib | 1.244 | (0.911,1.723) | 1.001 |
| hrd[5] | ceritinib vs crizotinib | 0.891 | (0.544,1.462) | 1.001 |
| hrd[6] | lorlatinib vs crizotinib | 0.713 | (0.413,1.24) | 1.001 |
| hrd[7] | ensartinib vs crizotinib | 0.931 | (0.564,1.521) | 1.001 |
