# Supplementary material for: NMAsurv: An R Shiny application for network meta-analysis based on survival data
Source: Res Synth Methods. 2025 Jul 10;16(6):1042–56. doi: 10.1017/rsm.2025.10020 (PMC12657653; doi:10.1017/rsm.2025.10020)
Supplement: Shao et al. supplementary material [file S1759287925100203sup001.zip › supporting information/supporting information 2.docx]

Supporting information 2

**Title:**

NMAsurv: An R shiny application for network meta-analysis based on survival data

**Author Information:**

Taihang Shao*^[1][2]^, Mingye Zhao*^[1]^, Fenghao Shi^[3]^, Mingjun Rui^[1][4]^, Wenxi Tang^#[1]^

[1] Center for Pharmacoeconomics and Outcome Research, China Pharmaceutical University, No. 639 Longmian Avenue, Nanjing 211198, China
[2] JC School of Public Health and Primary Care, Faculty of Medicine, The Chinese University of Hong Kong, Shatin, NT, Hong Kong SAR, China
[3] International Research Center for Medicinal Administration, Peking University, No.5 Yiheyuan Road, Haidian District, Beijing 100871, China
[4] School of Pharmacy, Faculty of Medicine, The Chinese University of Hong Kong, Shatin, NT, Hong Kong SAR, China

* These authors contributed equally to this work.

# Corresponding Author:

Wenxi Tang; Center for Pharmacoeconomics and Outcome Research, China Pharmaceutical University, No. 639 Longmian Avenue, Nanjing 211198, China; [tokammy@cpu.edu.cn](mailto:tokammy@cpu.edu.cn)

# Search strategy for PubMed

| **No** | **Items** | **Results** |
| --- | --- | --- |
| #1 | proportional hazard or PH-assumption or proportionality assumption or constant or var or non-proportional or nonproportional or time-varying or time-dependent or over-time or hazard or hazard-ratio or HR or time-ratio | [1,269,830](https://pubmed.ncbi.nlm.nih.gov/?term=(proportional+hazard%5bTitle/Abstract%5d+OR+PH-assumption%5bTitle/Abstract%5d+OR+proportionality+assumption%5bTitle/Abstract%5d+OR+constant%5bTitle/Abstract%5d+OR+var%5bTitle/Abstract%5d+OR+non-proportional%5bTitle/Abstract%5d+OR+nonproportional%5bTitle/Abstract%5d+OR+time-varying%5bTitle/Abstract%5d+OR+time-dependent%5bTitle/Abstract%5d+OR+over-time%5bTitle/Abstract%5d+OR+hazard%5bTitle/Abstract%5d+OR+hazard-ratio%5bTitle/Abstract%5d+OR+HR%5bTitle/Abstract%5d+OR+time-ratio%5bTitle/Abstract%5d)&ac=no&sort=relevance) |
| #2 | restricted mean or RMST or parametric or semiparametric or fractional polynomial or FP or accelerated failure time or AFT or piecewise or piece-wise or non-linear or nonlinear or flexible-regression or spline or b-spline or cox or Royston-Parmar or time-varying covariate or time-varying co-variate or mixture-cure or nonmixture cure or dynamic-survival or response-based or cure-model | [496,687](https://pubmed.ncbi.nlm.nih.gov/?term=(restricted+mean%5bTitle/Abstract%5d+OR+RMST%5bTitle/Abstract%5d+OR+parametric%5bTitle/Abstract%5d+OR+semiparametric%5bTitle/Abstract%5d+OR+fractional+polynomial%5bTitle/Abstract%5d+OR+FP%5bTitle/Abstract%5d+OR+accelerated+failure+time%5bTitle/Abstract%5d+OR+AFT%5bTitle/Abstract%5d+OR+piecewise%5bTitle/Abstract%5d+OR+piece-wise%5bTitle/Abstract%5d+OR+non-linear%5bTitle/Abstract%5d+OR+nonlinear%5bTitle/Abstract%5d+OR+flexible-regression%5bTitle/Abstract%5d+OR+spline%5bTitle/Abstract%5d+OR+b-spline%5bTitle/Abstract%5d+OR+cox%5bTitle/Abstract%5d+OR+Royston-Parmar%5bTitle/Abstract%5d+OR+time-varying+covariate%5bTitle/Abstract%5d+OR+time-varying+co-variate%5bTitle/Abstract%5d+OR+mixture-cure%5bTitle/Abstract%5d+OR+nonmixture+cure%5bTitle/Abstract%5d+OR+dynamic-survival%5bTitle/Abstract%5d+OR+response-based%5bTitle/Abstract%5d+OR+cure-model%5bTitle/Abstract%5d)&ac=no&sort=relevance) |
| #3 | (log-normal or log-logistic or gamma or gengamma or generalized or weibull or gompertz or exponential) | [723,513](https://pubmed.ncbi.nlm.nih.gov/?term=(log-normal%5bTitle/Abstract%5d+OR+log-logistic%5bTitle/Abstract%5d+OR+gamma%5bTitle/Abstract%5d+OR+gengamma%5bTitle/Abstract%5d+OR+generalized%5bTitle/Abstract%5d+OR+weibull%5bTitle/Abstract%5d+OR+gompertz%5bTitle/Abstract%5d+OR+exponential%5bTitle/Abstract%5d)&ac=no&sort=relevance) |
| #4 | #1 OR #2 OR #3 | 2,273,218 |
| #5 | network meta-analysis or network or indirect comparison or mixed-treatment or multiple treatment or evidence-based or MAIC or STC or ITC or MTC or NMA | 520,124 |
| #6 | time-to-event or survival or OS or PFS or duration-of-response or DOR or time-to-progression or TTP or time-to-failure or TTF or time-to-response or KM or Kaplan-Meier | 1,388,106 |
| #7 | y_10[Filter] | / |
| #8 | english [LA] | / |
| #9 | #4 AND #5 AND #6 AND #7 AND #8 | 7821 |

# Log hazard function formulas for four distributions

**Weibull:**

$$f(t)=a+b*\log(t)$$

**Gompertz:**

$$f(t)=a+b*t$$

**Log-Logistic:**

$$f(t)=\log(\frac{\frac{e^{b}}{e^{a}}*(\frac{t}{e^{a}})^{e^{b}-1}}{1+(\frac{t}{e^{a}})^{e^{b}}})$$

**Log-Normal:**

$$f(t)=\frac{(2\pi)^{-0.5}*e^{-\frac{(\frac{\log(t)-a}{e^{b}})^{2}}{2}}}{e^{b}*t*pnorm(-\frac{\log(t)-a}{e^{b}})}$$

# Probability density function for Generalized gamma model

Formula of Generalized Gamma Model (Q ≠ 0) can be found here.

$$PDF=f(\left. x \right|\mu,\sigma,Q)=\frac{\left| Q \right|(Q^{-2})^{Q^{-2}}}{\sigma t\Gamma(Q^{-2})}\exp[Q^{-2}(Qw-e^{Qw})]$$

In this formula, $\gamma\sim G\text{amma}(Q^{\text{-2}}\text{,1})$, $\omega=\log(Q^{2}\gamma)/Q$, $x=\exp(\mu+\sigma\omega)$, *t* is survival time, *μ* is the location parameter, *σ* is the scale parameter and *Q* is the shape parameter^46^. Please note that in this model, the treatment effect was dependent on the location parameter only. In addition, in this model,

$$\log(t_{ij})=x_{ij}\alpha$$

*x_ij_* is the treatment indicator variable for patient *i* from trial *j*, taking the value 0 if patient i receives the baseline treatment *a* and the value 1 if patient i receives treatment *b*. *μ* is the regression coefficient representing the treatment effect for treatment b compared to the baseline treatment a.

# R package used to construct this tool

| **Package** | **Verison** | **Package** | **Verison** | **Package** | **Verison** |
| --- | --- | --- | --- | --- | --- |
| broom | 1.0.7 | markdown | 1.13 | shinyBS | 0.61.1 |
| clipr | 0.8.0 | markdownInput | 0.1.2 | shinycssloaders | 1.1.0 |
| discSurv | 2.0.0 | MatrixModels | 0.5-3 | shinydashboard | 0.7.2 |
| doBy | 4.6.24 | metafor | 4.6-0 | shinydashboardPlus | 2.0.5 |
| dplyr | 1.1.4 | netmeta | 2.9-0 | shinyhelper | 0.3.2 |
| DT | 0.33 | officer | 0.6.7 | shinyjs | 2.1.0 |
| flexsurv | 2.3.2 | R.utils | 2.12.3 | shinymanager | 1.0.410 |
| flextable | 0.9.7 | R2jags | 0.8-9 | shinytitle | 0.1.0 |
| ggmcmc | 1.5.1.1 | rclipboard | 0.2.1 | shinyWidgets | 0.8.7 |
| ggplot2 | 3.5.1 | readxl | 1.4.3 | slickR | 0.6.0 |
| grid | 4.4.0 | rhandsontable | 0.3.8 | survHE | 2.0.2 |
| gridExtra | 2.3 | rmarkdown | 2.29 | survival | 3.8-3 |
| heemod | 1.0.2 | scales | 1.3.0 | survminer | 0.5.0 |
| knitr | 1.49 | shiny | 1.10.0 | tidyverse | 2.0.0 |
| lme4 | 1.1-36 | shinyalert | 3.1.0 | writexl | 1.5.1 |
| magrittr | 2.0.3 |  |  |  |  |

# PH assumption test results

**Schoenfeld residual plot**

| 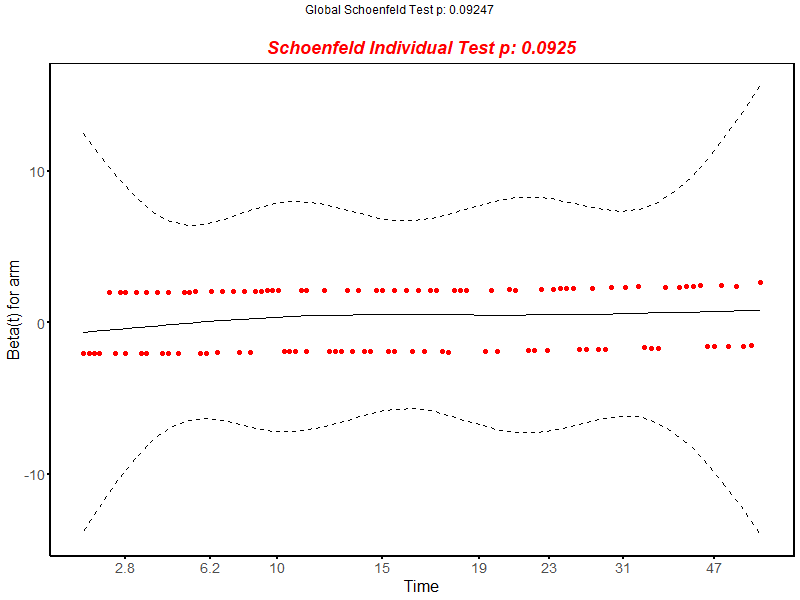  ALEX | 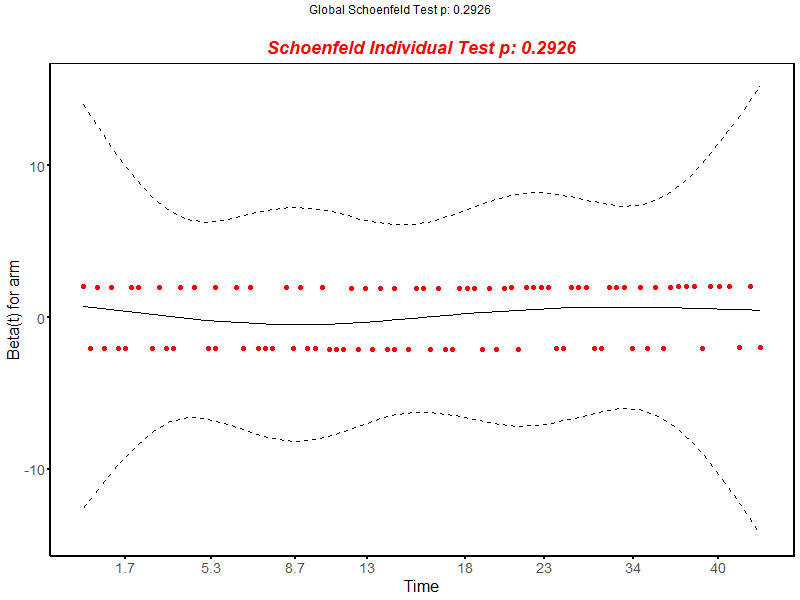  ALTA-1L |
| --- | --- |
| 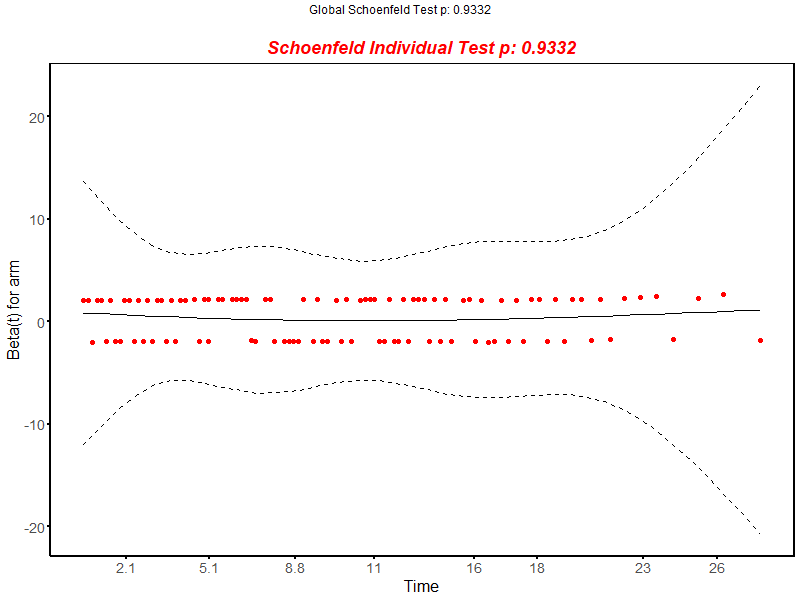  ASCEND-4 | 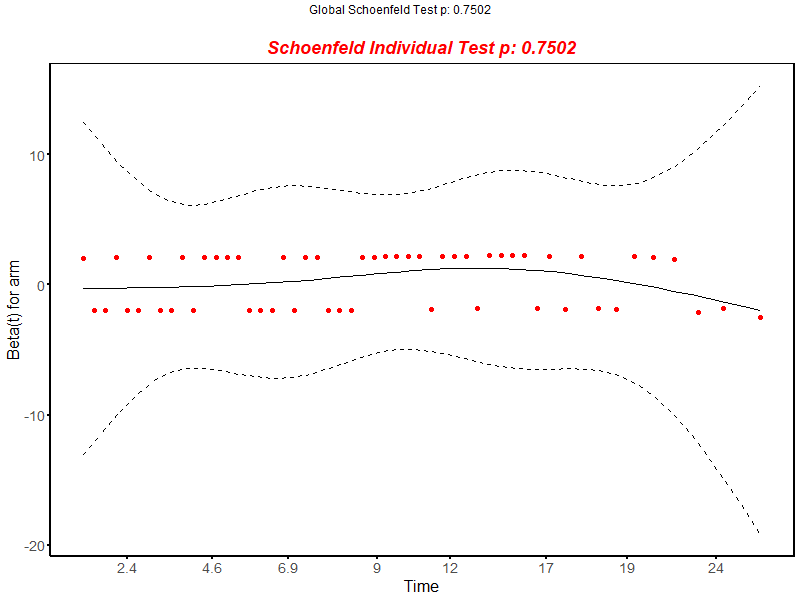  CROWN |
| 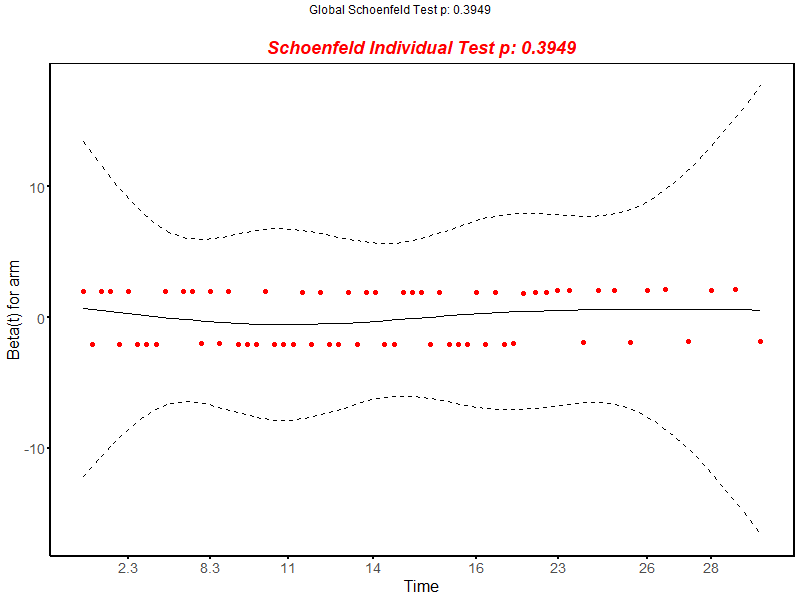  eXalt3 | 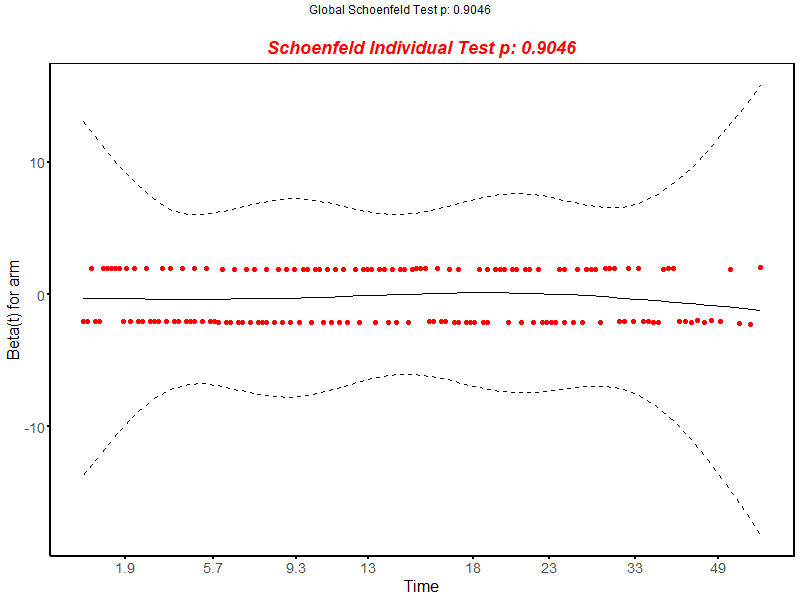  PROFILE 1014 |

**Log-Log plot**

| 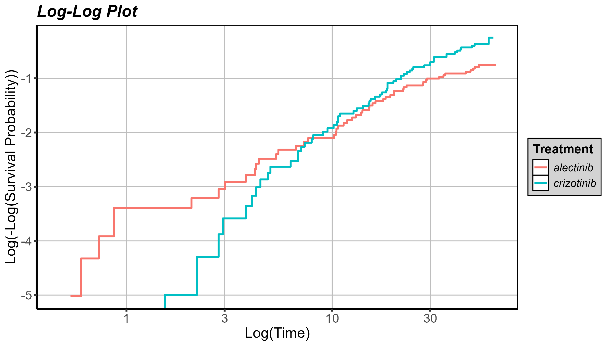  ALEX | 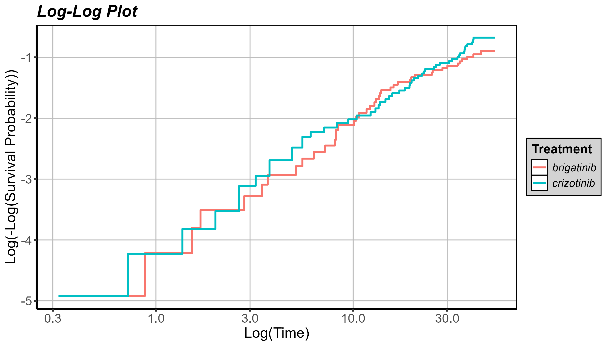  ALTA-1L |
| --- | --- |
| 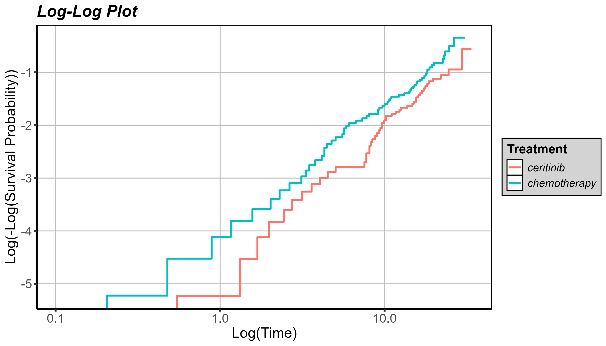  ASCEND-4 | 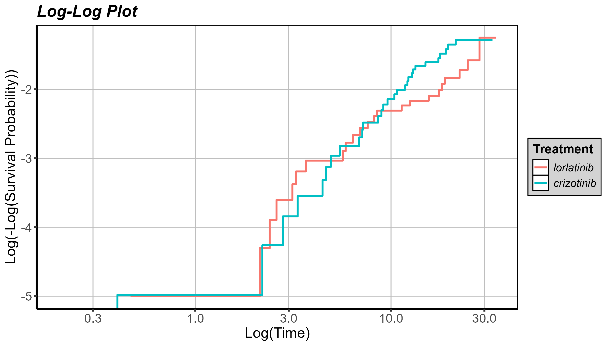  CROWN |
| 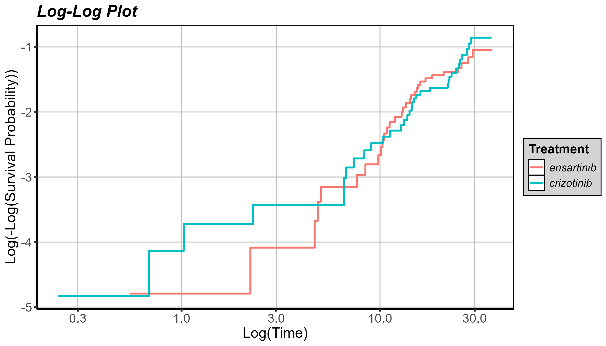  eXalt3 | 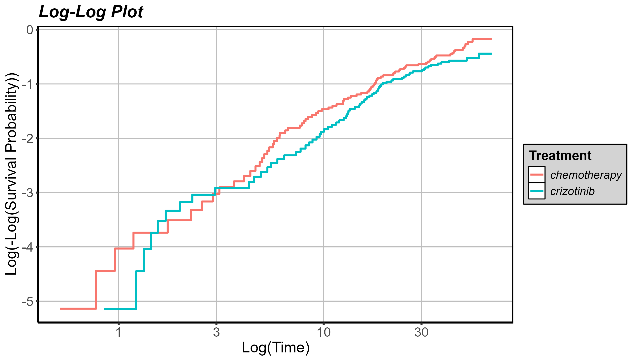  PROFILE 1014 |

**Grambsch-Therneau test**

| ALEX |  |  |  |
| --- | --- | --- | --- |
|  | chisq | df | p |
| arm | 2.83 | 1 | 0.092 |
| GLOBAL | 2.83 | 1 | 0.092 |
| ALTA-1L |  |  |  |
|  | chisq | df | p |
| arm | 1.11 | 1 | 0.29 |
| GLOBAL | 1.11 | 1 | 0.29 |
| ASCEND-4 |  |  |  |
|  | chisq | df | p |
| arm | 0.00703 | 1 | 0.93 |
| GLOBAL | 0.00703 | 1 | 0.93 |
| CROWN |  |  |  |
|  | chisq | df | p |
| arm | 0.101 | 1 | 0.75 |
| GLOBAL | 0.101 | 1 | 0.75 |
| eXalt3 |  |  |  |
|  | chisq | df | p |
| arm | 0.724 | 1 | 0.39 |
| GLOBAL | 0.724 | 1 | 0.39 |
| PROFILE 1014 |  |  |  |
|  | chisq | df | p |
| arm | 0.0144 | 1 | 0.9 |
| GLOBAL | 0.0144 | 1 | 0.9 |

# Results for FP model

**Relative treatment effects based on fixed effect model (Frequentist analysis)**

|  | est | std.err | conf.int.lower | conf.int.upper |
| --- | --- | --- | --- | --- |
| trtfalectinib | 0.088 | 0.33 | -0.56 | 0.735 |
| trtfbrigatinib | 0.136 | 0.388 | -0.625 | 0.897 |
| trtfchemotherapy | 0.154 | 0.284 | -0.403 | 0.711 |
| trtfceritinib | -0.25 | 0.515 | -1.26 | 0.759 |
| trtflorlatinib | -0.324 | 0.588 | -1.476 | 0.828 |
| trtfensartinib | 0.466 | 0.552 | -0.616 | 1.549 |
| trtfalectinib:f1 | -0.021 | 0.013 | -0.047 | 0.005 |
| trtfbrigatinib:f1 | -0.016 | 0.016 | -0.048 | 0.016 |
| trtfchemotherapy:f1 | 0.003 | 0.012 | -0.02 | 0.026 |
| trtfceritinib:f1 | 0.008 | 0.03 | -0.051 | 0.068 |
| trtflorlatinib:f1 | 0 | 0.039 | -0.075 | 0.075 |
| trtfensartinib:f1 | -0.03 | 0.029 | -0.086 | 0.026 |

**Results of mu based on fixed effect model (Bayesian analysis)**

|  | median | ci | Rhat |
| --- | --- | --- | --- |
| mu[1,1] | -3.931 | (-4.388,-3.517) | 1.264 |
| mu[2,1] | -4.175 | (-4.664,-3.635) | 1.02 |
| mu[3,1] | -3.914 | (-4.534,-3.403) | 1.007 |
| mu[4,1] | -4.177 | (-4.924,-3.467) | 1.003 |
| mu[5,1] | -4.911 | (-5.651,-4.125) | 1.025 |
| mu[6,1] | -3.703 | (-4.141,-3.349) | 1.048 |
| mu[1,2] | -0.016 | (-0.033,0.001) | 1.166 |
| mu[2,2] | -0.017 | (-0.039,0.001) | 1.016 |
| mu[3,2] | -0.006 | (-0.042,0.032) | 1.004 |
| mu[4,2] | -0.032 | (-0.084,0.016) | 1.003 |
| mu[5,2] | 0.018 | (-0.024,0.054) | 1.016 |
| mu[6,2] | -0.031 | (-0.048,-0.013) | 1.022 |

**Results of d based on fixed effect model (Bayesian analysis)**

|  | comparison | median | ci | Rhat |
| --- | --- | --- | --- | --- |
| d[1,1] | crizotinib vs crizotinib | 0 | (0,0) | 1 |
| d[2,1] | alectinib vs crizotinib | 0.043 | (-0.594,0.669) | 1.136 |
| d[3,1] | brigatinib vs crizotinib | 0.151 | (-0.63,0.858) | 1.008 |
| d[4,1] | chemotherapy vs crizotinib | 0.106 | (-0.417,0.675) | 1.038 |
| d[5,1] | ceritinib vs crizotinib | -0.299 | (-1.275,0.733) | 1.013 |
| d[6,1] | lorlatinib vs crizotinib | -0.336 | (-1.521,0.802) | 1.004 |
| d[7,1] | ensartinib vs crizotinib | 0.547 | (-0.594,1.612) | 1.02 |
| d[1,2] | crizotinib vs crizotinib | 0 | (0,0) | 1 |
| d[2,2] | alectinib vs crizotinib | -0.02 | (-0.046,0.006) | 1.082 |
| d[3,2] | brigatinib vs crizotinib | -0.017 | (-0.048,0.014) | 1.007 |
| d[4,2] | chemotherapy vs crizotinib | 0.005 | (-0.018,0.028) | 1.019 |
| d[5,2] | ceritinib vs crizotinib | 0.01 | (-0.05,0.067) | 1.005 |
| d[6,2] | lorlatinib vs crizotinib | 0 | (-0.077,0.078) | 1.003 |
| d[7,2] | ensartinib vs crizotinib | -0.034 | (-0.09,0.025) | 1.011 |

**Hazard plot for fixed effect model (Frequentist analysis)**


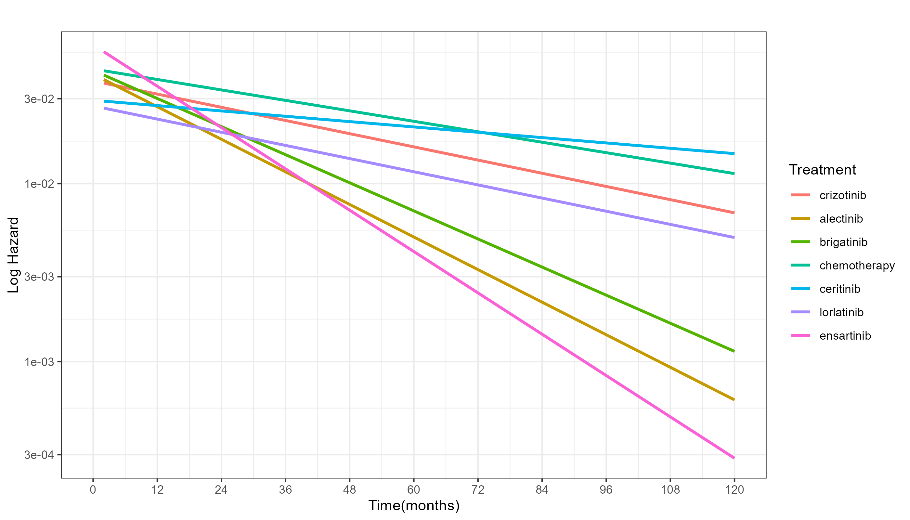


**Survival plot for fixed effect model (Frequentist analysis)**


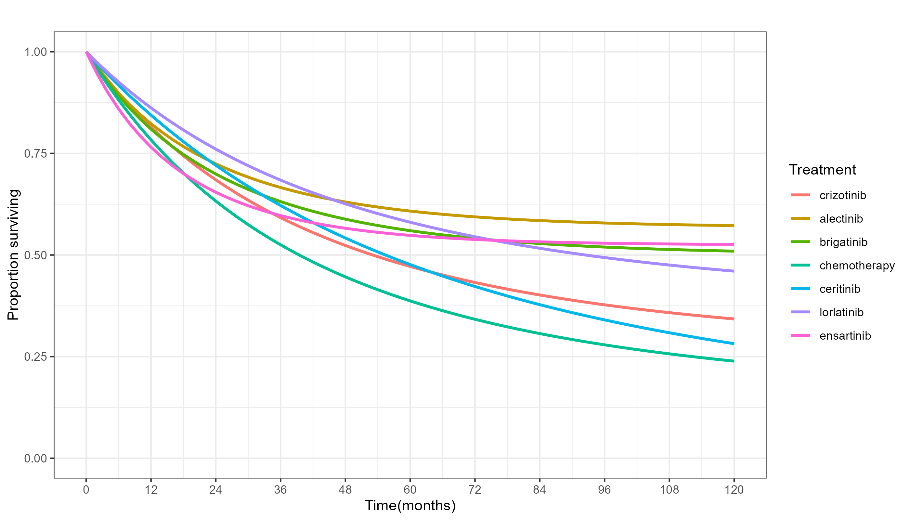


**HR plot for fixed effect model (Frequentist analysis)**


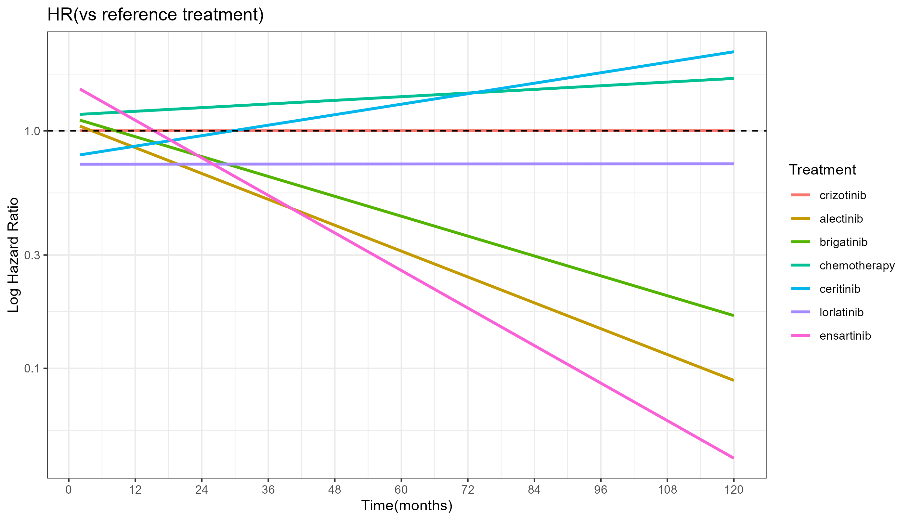


**Hazard plot for fixed effect model (Bayesian analysis)**


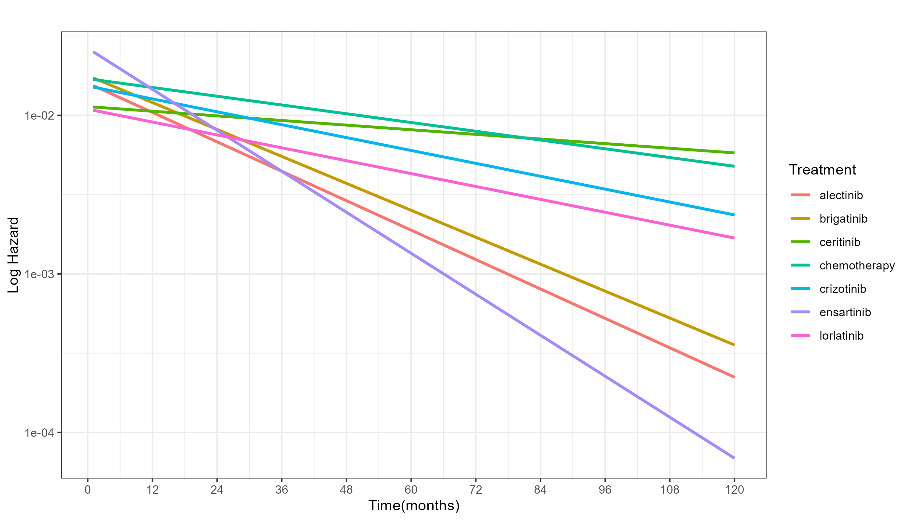


**Survival plot for fixed effect model (Bayesian analysis)**


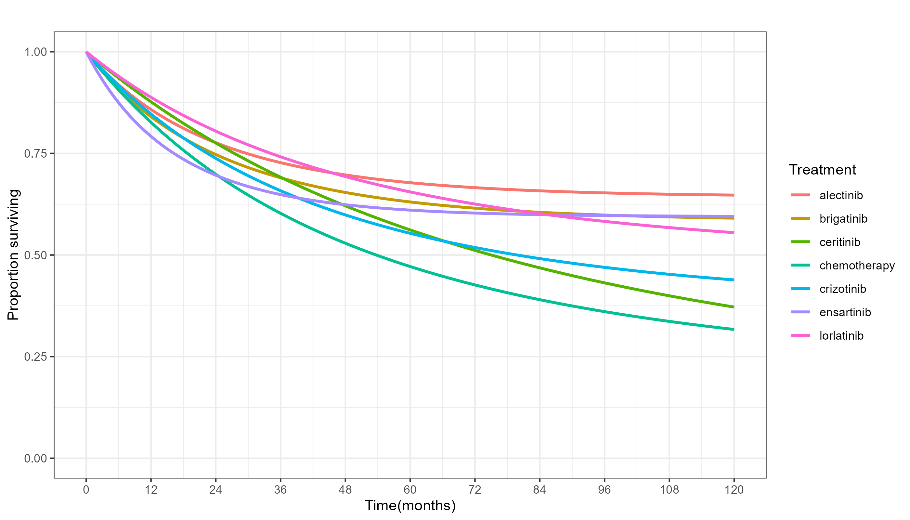


**HR plot for fixed effect model (Bayesian analysis)**


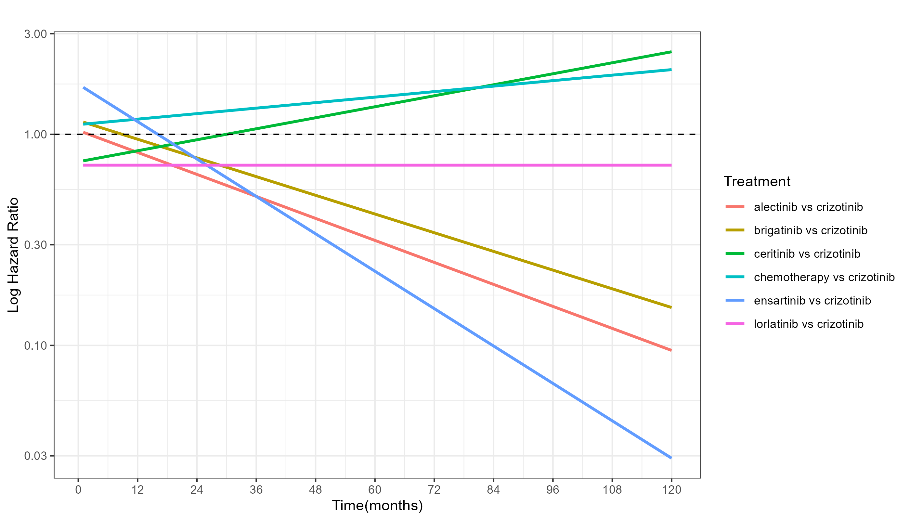


# Results for Cox-PH model

|  | comparison | median | ci | Rhat |
| --- | --- | --- | --- | --- |
| hrd[2] | alectinib vs crizotinib | 0.706 | (0.487,1.011) | 1.001 |
| hrd[3] | brigatinib vs crizotinib | 0.824 | (0.553,1.245) | 1.001 |
| hrd[4] | chemotherapy vs crizotinib | 1.245 | (0.915,1.719) | 1.002 |
| hrd[5] | ceritinib vs crizotinib | 0.886 | (0.538,1.46) | 1.002 |
| hrd[6] | lorlatinib vs crizotinib | 0.721 | (0.421,1.246) | 1.002 |
| hrd[7] | ensartinib vs crizotinib | 0.932 | (0.566,1.543) | 1.001 |
